# Sustainable Liquid Metal Composites for Soft Electronics and E‐Waste Reduction

**DOI:** 10.1002/adma.202512968

**Published:** 2025-11-19

**Authors:** Abdollah Hajalilou, Elahe Parvini, Carmel Majidi

**Affiliations:** ^1^ CENIMAT|i3N Department of Materials Science School of Science and Technology NOVA University Lisbon and CEMOP/UNINOVA Campus de Caparica Caparica 2829‐516 Portugal; ^2^ Institute of Systems and Robotics Department of Electrical Engineering University of Coimbra Coimbra 3030‐290 Portugal; ^3^ Integrated Soft Materials Lab Department of Mechanical Engineering Carnegie Mellon University Pittsburgh PA 15213 USA

**Keywords:** eco‐friendly recycling, E‐waste reduction, liquid metal composites, soft electronics, sustainable and self‐healable materials

## Abstract

There has been tremendous progress in soft printable electronics with liquid metal (LM) for creating elastic, stretchable, and thin‐film circuitry. However, such innovation risks outpacing sustainability. As the world moves toward softer, thinner, and more integrated electronics, these advancements may accelerate the exponential rise of electronic waste—projected to surpass 74 million tons by 2030 and reach ≈120 million tons by 2050. Therefore, in developing LM‐based electronics, it is critical to address the urgent need for eco‐friendly recycling strategies. This review proposes a paradigm shift toward recyclable, repairable, renewable, and resilient (“4R”) soft electronics enabled by LM composites. Gallium‐based alloys and their eutectics, such as Galinstan and EGaIn, offer a rare convergence of high conductivity, fluidic deformability, and recyclability—ideally suited for stretchable circuits, soft robotics, and wearables. Yet, their promise remains incomplete without scalable, eco‐friendly recovery methods. Advanced extraction and recycling approaches are examined, including mechanical–chemical and physicochemical methods using deep eutectic solvents (DESs) and ionic liquids for selective recovery with minimal environmental impact. Emphasis is placed on materials selection, substrate compatibility, and integration strategies that enable circular lifecycles. LM composites offer a path to redefine electronics—not only for performance but also for durability, self‐healing, and sustainability.

## Graphical abstract

Liquid metal (LM) composites embody recyclability, repairability, renewability, and resilience (4R), offering a sustainable route for soft electronics. With exceptional conductivity, stretchability, and eco‐friendly recovery, these systems enable closed‐loop lifecycles and reduce e‐waste. Their integration paves the way for wearable, flexible, and recyclable devices that support a circular electronics economy.

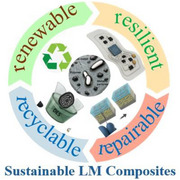

## 1 Introduction

The rapid proliferation of electronic devices — ranging from smartphones to smart textiles — has led to an unprecedented rise in electronic waste (e‐waste). Global e‐waste is projected to reach 74.7 million metric tons by 2030 and exceed 110 million metric tons by 2050.^[^ ^1^, ^2^, ^3^, ^4^ ^]^ In 2021 alone, over 52 million metric tons of e‐waste were generated globally, yet less than 20% was properly recycled, leading to significant environmental and health concerns about accumulated heavy metals and persistent pollutants.^[^ ^5^, ^6^, ^7^ ^]^ The increasing reliance on electronics for communication, healthcare, wellness, and at‐home monitoring, entertainment, and productivity has fueled their mass production and consumption, contributing significantly to the e‐waste crisis.^[^ ^8^ ^]^ On average, e‐waste production has reached about 7 kg per person per year, but only ≈20% is recycled. Recovery of precious metals, particularly gold, remains minimal.^[^ ^9^, ^10^, ^11^ ^]^ The emergence of smart packaging, printed electronics, non‐clinical, at‐home disposable health‐monitoring patches, sensorized diapers, and e‐textiles is expected to add further to this problem, potentially introducing billions of short‐lifecycle electronic devices in the coming decades.^[^ ^11^ ^]^

Because of the mounting challenges with e‐waste, it is increasingly important that new material technologies be designed with sustainability, recyclability, and circularity in mind, rather than focusing solely on performance metrics. In this review, we therefore frame fabrication and materials discussions explicitly through their implications for end‐of‐life recovery and the 4R strategy. This is particularly relevant for soft and flexible devices intended for consumer or at‐home use that ultimately enter household e‐waste streams. Indeed, the increasing demand for adaptable and multifunctional materials has driven innovation in soft and stretchable systems.^[^ ^12^, ^13^, ^14^, ^15^ ^]^ While traditional engineered materials, such as metals and ceramics, offer strength and durability, they are inherently rigid and brittle, making them unsuitable for applications requiring flexibility and adaptability.^[^ ^16^, ^17^ ^]^ In contrast, soft electronics— inspired by the properties of biological tissues—offer a compelling alternative to conventional rigid devices. Their ability to conform to irregular surfaces and endure repeated mechanical deformation makes them ideal for integration with the human body and other soft, dynamic environments.^[^ ^18^ ^]^ Biological systems are naturally soft, resilient, and multifunctional, motivating the development of materials that mimic these characteristics for technological applications. As a result, soft machines and stretchable electronics have emerged as transformative platforms, enabling innovation in fields such as wearable health monitoring, soft robotics, electronic skin (e‐skin), flexible displays, e‐textiles, and energy storage or harvesting systems.^[^ ^19^, ^20^, ^21^, ^22^, ^23^, ^24^, ^25^, ^26^ ^]^ The market potential of these technologies is significant. For instance, the global wearable technology market, valued at $40 billion in 2020, is expected to grow to $300 billion by 2050, driven by increasing demand in healthcare and consumer electronics. Similarly, the e‐skin market, worth $19 billion in 2020, is projected to exceed $50 billion by 2050, fueled by advances in human–machine interfaces and prosthetic devices. In parallel, supercapacitors—vital for stretchable energy storage—are forecasted to grow from $3.1 billion in 2021 to over $30 billion by 2050, reflecting the rising demand for sustainable, high‐performance power systems. However, the emerging and accelerating adoption of soft electronics introduces an important environmental concern: their contribution to the growing electronic waste (e‐waste) stream. With billions of soft and wearable systems projected in the near future—often embedded with miniaturized circuits, sensors, and rare metals—proactive strategies are needed to address long‐term ecological and resource‐recovery challenges. Although soft and wearable electronics remain a relatively small fraction of the overall electronics market, their accelerating growth trajectory makes it essential to integrate sustainability early. For instance, the wearable technology market was valued at approximately USD 84.2 billion in 2024 and is projected to grow to USD 186.1 billion by 2030.^[^ ^27^, ^28^ ^]^ In contrast, the smartphone market, estimated at USD 520.45 billion in 2024, continues to dominate globally.^[^ ^29^ ^]^ This disparity indicates that soft electronics are not yet as widespread as rigid and flexible devices. Nonetheless, their rapid adoption in consumer and healthcare contexts—many with short lifecycles—underscores the importance of designing with recyclability and the 4R principles in mind.^[^ ^11^, ^30^, ^31^ ^]^ Therefore, while soft electronics are currently a niche compared to rigid systems, their projected growth trajectory justifies proactive consideration of recyclability and the 4R framework at this stage. In parallel, we emphasize hybrid devices with rigid “islands” (integrated circuits (ICs) and power management) interconnected by soft LM‐based conductors and sensors, where materials choice can directly impact disassembly and recovery. Because soft devices are not separately tracked in most e‐waste statistics and are typically grouped into “small equipment/IT”, we highlight design labels, take‐back readiness, and LM–polymer formulations that enable practical small‐device recovery as complementary levers to conventional (rigid‐centric) recycling.^[^ ^32^ ^]^

Concerns about environmental sustainability are especially acute for soft devices designed for frequent replacement in non‐clinical settings—such as at‐home monitoring patches, fitness wearables, and consumer e‐skin products.^[^ ^33^, ^34^, ^35^, ^36^ ^]^ These items are more likely to enter household e‐waste streams where capture rates are low, heightening the need for materials and architectures that enable recovery and recycling. Conventional recycling strategies for rigid electronics—including mechanical shredding, pyrometallurgical smelting, and hydrometallurgical leaching—enable only partial recovery of valuable materials and are hindered by low selectivity, high energy cost, and secondary pollution.^[^ ^37^, ^38^, ^39^ ^]^ While such approaches provide conceptual starting points, their direct transfer to LM‐based soft systems is limited by the heterogeneous nature of these composites, where fluidic alloys are embedded within elastomers or gels. Recent work highlights emerging solutions tailored to this challenge, including reconfigurable LM–polymer composites,^[^ ^40^ ^]^ vitrimer–LM systems enabling closed‐loop recovery,^[^ ^41^ ^]^ and dissolvable LM‐based circuits with near‐complete recovery of both metal and polymer phases.^[^ ^42^ ^]^ Therefore, the potential growth in disposable electronics highlights the urgent need for soft and flexible materials that can balance functionality, adaptability, and sustainability.

Managing the disposal of soft electronic waste (SEW) can be especially challenging due to the possible chemical complexity and toxicity of the constituent materials. These, in turn, can result in laborious, expensive, and resource‐intensive processes. Broadly speaking, only a small portion of e‐waste—6.5 million metric tons out of 41.8 million—was recycled in 2014.^[^ ^43^ ^]^ As electronics become more integrated into everyday materials, a new strategy is needed—one that considers recyclability, repairability, renewability, and resilience (the 4R principle) from the outset of device design. The traditional “3R” strategies (reduce, reuse, recycle), though widely promoted, are insufficient for SEW (soft electronic waste) due to hygienic limitations, contamination, and the difficulty of disassembling soft hybrid structures.^[^ ^11^ ^]^ Moreover, most current systems still rely on conventional solid‐state conductive fillers—such as silver flakes, carbon nanotubes, or graphene—which suffer from poor stretchability, irreversible conductivity loss under strain, mechanical fatigue, agglomeration, and limited recyclability.^[^ ^44^, ^45^, ^46^, ^47^ ^]^ Their high percolation thresholds require large filler loadings, compromising flexibility, while poor interfacial bonding and brittleness hinder long‐term performance. To address these limitations, liquid metals (LMs)—notably gallium and its eutectic alloys—are being explored as next‐generation fillers for soft electronics. LM–polymer composites offer exceptional electrical and thermal conductivity, ultra‐high deformability, and self‐healing capabilities that restore conductivity via LM droplet flow.^[^ ^20^, ^21^, ^48^, ^49^, ^50^, ^51^ ^]^ They are particularly attractive for consumer and at‐home wearable applications where mechanical adaptability is critical. Additionally, LM inclusion enhances thermal management, a key requirement for densely packed, flexible electronic systems. Recent processing strategies—including ultrasonication, microfluidics, and nebulization—have significantly improved LM dispersion within elastomer matrices, resulting in enhanced mechanical and functional performance.^[^ ^21^, ^22^, ^52^ ^]^ Despite these advantages, challenges remain. LM composites often require mechanical or thermal sintering, exhibit poor adhesion to substrates, and suffer from limited patterning resolution—obstacles that hinder industrial scalability.^[^ ^53^, ^54^, ^55^ ^]^ In parallel, recyclable and biodegradable materials have been proposed for transient electronics, yet these often lack the durability needed for real‐world deployment.^[^ ^56^, ^57^ ^]^

This review article focuses on methods to engineer soft electronics with liquid metal that can be environmentally sustainable. In particular, we present fabrication and materials choices specifically through their consequences for end‐of‐life recovery (4R), highlighting LM systems and composite architectures that enable practical disassembly and recycling. These included so‐called LM‐embedded elastomer (LMEE) composites composed of LM microdroplets suspended in a soft rubbery matrix, as well as “biphasic” LM composites, which included the addition of silver flakes or other rigid filler particles and typically utilized either chemically cross‐linked elastomers (e.g., polydimethylsiloxane (PDMS), polyurethane (PU)) or thermoplastic block copolymers (e.g., styrene‐isoprene‐styrene (SIS), styrene‐ethylene‐butylene‐styrene (SEBS), thermoplastic polyurethane (TPU)) as the polymer matrix material. Throughout, examples drawn from clinical/sterile single‐use contexts are cited only to illuminate materials behavior; they are not proposed targets for recycling unless decontaminated and routed through e‐waste channels. Importantly, we distinguish two matrix classes from the outset: i) chemically cross‐linkedcross‐linked elastomers (e.g., PDMS/Ecoflex), which are insoluble networks and poorly suited to solvent reclamation,^[^ ^58^ ^]^ requiring alternative recovery strategies such as alkali/acid‐assisted LM liberation or depolymerization; and ii) physically cross‐linked/thermoplastic elastomers (e.g., SIS/SEBS/TPU), which are solvent‐ and/or melt‐processable and therefore compatible with solvent‐assisted or thermal reprocessing.^[^ ^59^ ^]^ This classification is critical, since solvent reclamation routes apply primarily to thermoplastic systems, while chemically cross‐linked systems face the same end‐of‐life challenges as bulk rubbers (e.g., tires). More recently, a third emerging class is vitrimer networks, which feature dynamic covalent bonds that combine the durability of thermosets with the reprocessability of thermoplastics. Vitrimer–LM composites have been shown to enable recyclability, thermal healing, and closed‐loop reprocessing, providing a promising pathway for sustainable soft electronics.^[^ ^41^, ^60^, ^61^ ^]^ By analyzing these material systems through the 4R lens, we highlight not only their performance attributes but also practical e‐waste reduction via design‐for‐recovery. This review therefore bridges conventional fabrication knowledge with sustainability outcomes, making explicit how processing choices dictate recycling feasibility. These systems enable sinter‐free, nonsmearing interconnects with improved printability, flexibility, and recyclability.^[^ ^21^, ^22^, ^62^ ^]^ Advances in high‐resolution laser patterning and digital printing techniques further enhance recyclability, multilayer integration, and fabrication scalability—bringing soft electronics closer to industrial standards in terms of performance and manufacturability.^[^ ^11^, ^19^, ^53^ ^]^ Because recyclability is determined at the stage of materials selection and composite design, this review begins by outlining LM material systems and fabrication strategies that inherently influence end‐of‐life pathways. Here, fabrication is not presented for performance alone, but rather through the lens of the 4R principle. This framing establishes materials choice as the fundamental step toward reducing e‐waste before discussing dedicated recycling technologies.

Despite notable progress, e‐waste recycling of LM‐based composites remains critically underdeveloped. It is important to note, however, that recycling of conventional rigid electronics is also notoriously inefficient, with global capture and recovery rates still very low despite decades of optimization. Current infrastructures are primarily designed around rigid device architectures and rely on mechanical shredding, pyrometallurgical smelting, and hydrometallurgical leaching. Certain elements of these strategies—such as solvent‐assisted delamination, electrochemical metal recovery, and selective wet etching—may provide conceptual starting points for LM‐based devices. Yet, their direct transfer is limited by the heterogeneous and dynamic nature of soft systems, where fluidic alloys are intimately embedded within elastomers, gels, or dynamic covalent networks. Conventional high‐temperature or mechanical approaches risk destroying both the recoverable LM phase and the surrounding polymer substrate, thereby undermining recyclability. These unique challenges highlight the need for tailored approaches—such as oxide‐aware release chemistries (i.e., strategies that dissolve or reduce the native Ga_2_O_3_ skin to enable LM reflow and recovery), deep eutectic solvents, vitrimer–LM systems, and dissolvable LM circuits—that enable the recovery of multiple components without excessive energy input or material loss. Efficient, scalable SEW recycling methods—particularly for LM‐based systems—are urgently needed to reduce environmental pollution, conserve finite resources, and promote a circular electronics economy. Designing materials and devices with recyclability and disassembly in mind will be essential to ensure that the rapid evolution of soft electronics aligns with environmental and societal sustainability. Ultimately, bridging the gap between innovation and responsible end‐of‐life strategies is crucial to enabling the next generation of sustainable, high‐performance electronics.

## 2 Industrialization, Economics, and Sustainability Outlook for LM Composites

The sustainability discussion around soft and stretchable electronics must be grounded in both the current industrial landscape and realistic projections of their contribution to electronic waste. At present, rigid devices based on hard substrates (e.g., Flame Retardant‐4 (FR‐4) epoxy/glass fiber–reinforced printed circuit boards (PCBs), epoxy resin encapsulants, and rigid polymer housings) dominate global e‐waste streams by weight.^[^ ^63^ ^]^ Soft electronics, including LM–based systems, remain a relatively small fraction in terms of mass. Nevertheless, their adoption is accelerating rapidly in terms of unit volume, particularly in the domains of consumer wearables, health monitoring patches, and flexible electronic skins.^[^ ^64^ ^]^ These devices typically have short lifecycles, are often discarded without entering formal recycling streams, and are widely dispersed in households. While their current contribution to e‐waste is modest, the prospective and precautionary urgency lies in preventing the mistakes of rigid electronics from being replicated as soft systems scale up.

From a cost perspective, gallium‐based alloys already offer advantages: the price of Ga–In–Sn eutectics has been reported to be lower than that of silver‐based conductive fillers, making LM inks a potentially cost‐effective substitute for silver nanoparticle inks or flake‐based conductive pastes that dominate today's printed electronics.^[^ ^65^ ^]^ Gallium pricing, however, depends strongly on purity. Commodity‐grade gallium (99.99%) averaged $240 kg^−1^ in June 2023 and $380 kg^−1^ in June 2024.^[^ ^66^ ^]^ By comparison, the silver spot price in the same period was ≈$1350 kg^−1^, making low‐purity gallium roughly one‐quarter to one‐fifth the cost of silver. At the high end, 6N (99.9999%) gallium used in semiconductors sells at ≈$1200 kg^−1^, approaching parity with silver prices. This distinction underscores the importance of defining grade and application when assessing economic competitiveness.

From an industrial perspective, LM‐based printed electronics (LMPE) illustrate the transition from fundamental science to commercialization. Over the past two decades, LMPE has progressed from laboratory demonstrations to prototype machines and then to market‐ready products. Early milestones included direct‐writing systems and LM‐based circuit printers capable of producing functional circuits without complicated post‐processing.^[^ ^67^, ^68^ ^]^ Today, a portfolio of LM‐related products is available, such as LM electronic inks, printing substrates, packaging materials, writing pens, and automated printers.^[^ ^69^, ^70^ ^]^ By avoiding traditional, multi‐step PCB manufacturing, LMPE offers a shorter design–production cycle, reduced chemical waste, and opportunities for personalized or small‐batch fabrication.

Industrialization of LM composites is advancing rapidly. Galinstan was industrialized as early as the 1990s, while the experimental introduction of EGaIn dates back to the early 2000s. A key milestone was the 2008 publication demonstrating the use of eutectic gallium–indium (EGaIn) in microchannels at room temperature.^[^ ^71^ ^]^ Commercial LM pens, inks, and printers are already available, and large‐scale adoption has been demonstrated in mass‐market devices such as the PlayStation 5, which uses a gallium‐based liquid‐metal thermal interface material.^[^ ^30^ ^]^ Reports also suggest that PS5 Pro maintains liquid‐metal cooling in its updated thermal management system.^[^ ^72^ ^]^ In Europe, Metlen Energy & Metals announced an investment of about €296 million to establish the EU's first commercial gallium production project since 2016, aiming to reach ≈50 t per year capacity by 2028.^[^ ^73^ ^]^ In North America, Rio Tinto, in collaboration with Indium Corporation, reported in 2025 that they had extracted primary gallium at a pilot facility in New York, with plans to scale up to ≈40 t per year from a refinery in Quebec.^[^ ^74^ ^]^ Industrial suppliers such as Indium Corporation also actively market gallium alloys for semiconductors and thermal management,^[^ ^75^ ^]^ while Aster Materials supplies Galinstan for industrial thermal conductive layers.^[^ ^76^ ^]^ The industrial roadmap also highlights the importance of enabling infrastructure: the approval of a national standard for gallium‐based LMs in China (GB/T 39859‐2021) was a milestone, providing consistency and regulatory support for product development.^[^ ^77^ ^]^ Industrialization has shown the typical trajectory of emerging technologies—initial excitement and challenges in scaling and cost reduction, followed by steady growth as more mature products and killer applications emerge.^[^ ^78^, ^79^ ^]^ These developments indicate that gallium is no longer confined to laboratory‐scale use but is entering the commodity production pipeline, providing a stronger raw‐material base for LM inks, composites, and device manufacturing at an industrial scale.

**Figure 1:**
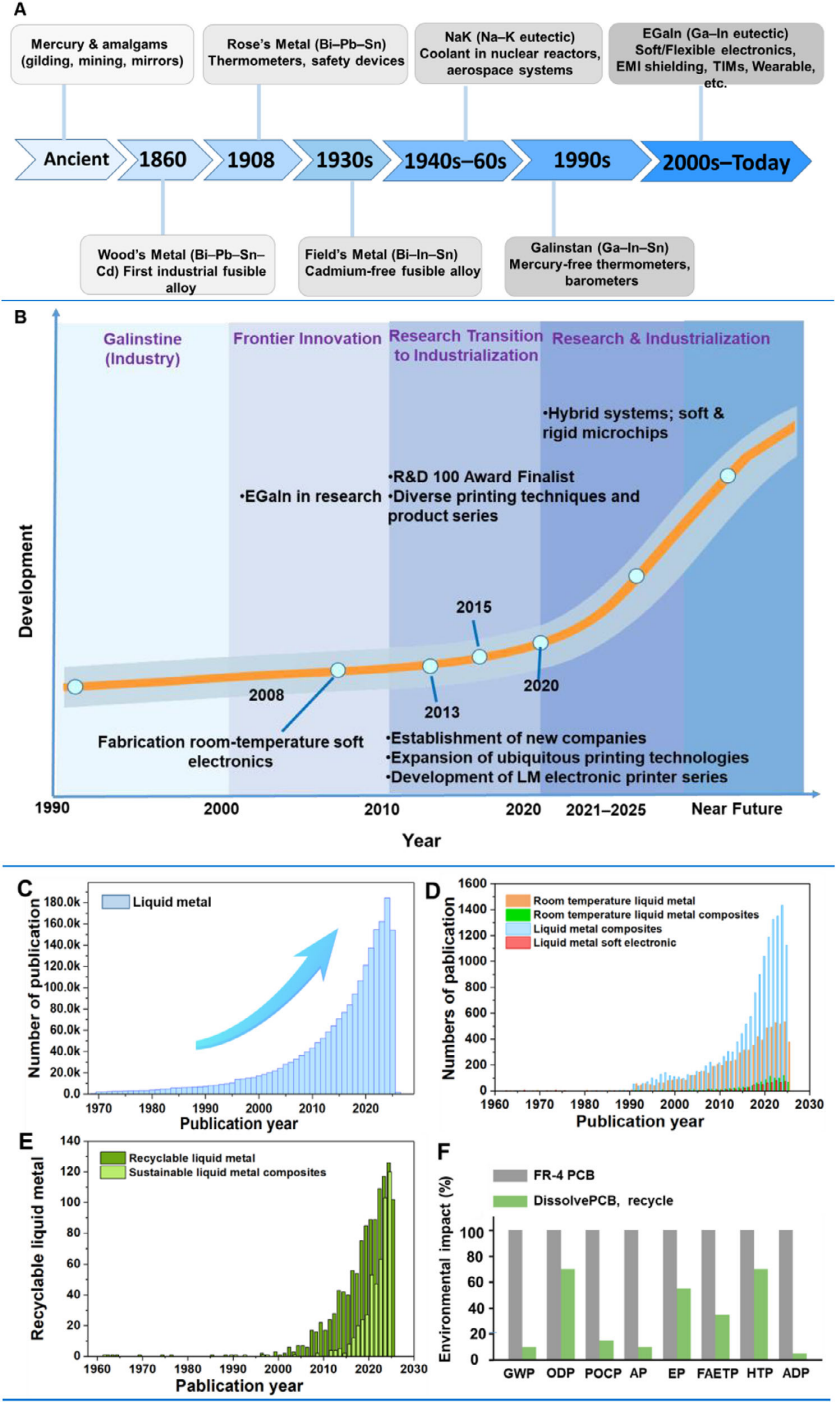
A) Historical timeline of liquid metal systems and fusible alloys, from ancient mercury amalgams to modern gallium‐based alloys such as Galinstan (1990s) and EGaIn (2000s), highlighting representative compositions and their applications in electronics, robotics, thermal management, and wearable devices. B) Research and industrial roadmap of liquid metals (1990–near future), illustrating the transition through four phases: early industrial use of Galinstine, frontier innovation in the 2000s, research transition to industrialization during the 2010s, and accelerated research & industrialization in the 2020s. Key milestones include the fabrication of room‐temperature soft electronics (2008), the establishment of new companies and LM printing technologies (2013), R&D 100 Award recognition and product diversification (2015), and the development of hybrid systems and soft/rigid microchips (2021–2025). C–E) Publication trends in liquid metal research based on keyword searches in Web of Science: C) “Liquid metal”; D) “Room temperature liquid metal”, “Room temperature liquid metal composites”, “Liquid metal composites”, and “Liquid metal soft electronics”; E) “Recyclable liquid metal” and “Sustainable liquid metal composites”. F) Life cycle assessment (LCA) comparison of conventional FR‐4 PCB and DissolvPCB, showing standardized LCIA results with recycling benefits. Reproduced with permission,^[^ ^42^ ^]^ Copyright 2025, ACM UIST Proceedings. GWP, global warming potential; ODP, ozone depletion potential; POCP, photochemical ozone creation potential; AP, acidification potential; EP, eutrophication potential; FAETP, freshwater aquatic ecotoxicity potential; HTP, human toxicity potential; ADP, abiotic depletion potential.

Taken together, these industrial advances can be viewed as part of a much longer historical trajectory of liquid metals. From ancient mercury amalgams to 19th–20th century fusible alloys, liquid metals steadily progressed as functional and industrial materials, culminating in modern gallium‐based alloys such as Galinstan (1990s) and EGaIn (2000s). Since then, research milestones such as LM–polymer composites and self‐healing systems have driven the field into a new phase. Today, industrialization (post‐2020) is accelerating toward wearables, EMI shielding, batteries, and robotics, marking the transition from historical alloys to modern large‐scale applications (**Figure** **1**A,B). The growing scientific interest in liquid metals is evident from publication trends retrieved from Web of Science (Figure 1C–E). A sharp rise in studies on “liquid metal” has been observed over the past two decades, reflecting their expanding role in electronics, energy, and materials science. More specific keyword searches, including “room temperature liquid metal”, “liquid metal composites”, and “liquid metal soft electronics”, highlight the rapid increase in research on functional materials and device applications. In addition, recent years have shown a surge in publications using terms such as “recyclable liquid metal” and “sustainable liquid metal composites”, emphasizing the growing importance of sustainability in this field.

While these trajectories highlight the rapid industrial and research expansion of LM composites, it is equally critical to evaluate their environmental implications. As shown in Figure 1D, a cradle‐to‐grave life cycle assessment (LCA) comparing conventional FR‐4 PCBs with DissolvPCB quantifies how design‐for‐recovery in LM systems translates into tangible sustainability gains.^[^ ^42^ ^]^ Significant reductions are observed across global warming potential, acidification potential, ozone depletion, human toxicity, eutrophication, and ecotoxicity, underscoring the environmental advantages of recyclable LM‐based systems over conventional rigid substrates.

In terms of the printed electronic waste viewpoint, while silver is the incumbent conductor in printed and flexible electronics, the broader e‐waste economy is driven primarily by copper (by mass) and gold (by economic value). According to the Global E‐waste Monitor 2024, of the 62 million tonnes of e‐waste generated in 2022, an estimated 8–9 million tonnes were recoverable metals. Copper represented the bulk share by mass—around 3–4 million tonnes—while precious metals such as gold (≈300 t) and silver (≈4000–5000 t) were present in much smaller amounts. However, these precious metals contributed disproportionately to recycling revenues: gold alone accounted for more than half of the total economic value of recoverable metals, despite being less than 0.01% of e‐waste by weight. This contrast underscores why copper drives recycling by volume, gold by value, and silver by its unique role in printed and flexible electronics. LM composites thus need to be framed within this broader landscape: while they may primarily displace silver‐based conductors in flexible systems, their recyclability contributes to overall recovery efficiency and could indirectly reduce reliance on both copper‐ and gold‐intensive circuits by enabling new hybrid architectures.

Economic and sustainability impacts must also be considered in the context of regulatory frameworks that increasingly emphasize recyclability and circularity. For example, in the European Union, the WEEE Directive (2012/19/EU) sets collection targets of 65% of average EEE placed on the market or 85% of WEEE generated; however, the actual EU collection rate in 2022 was 40.6%, underscoring the gap to the target and the need for design‐for‐recovery solutions.^[^ ^80^ ^]^ The companion RoHS (2011/65/EU) framework restricts hazardous substances, steering materials choices toward safer, more recyclable conductors. In the United States, the EPA promotes sustainable management of electronics and batteries^[^ ^81^ ^]^ and encourages safer material selection through programs such as Safer Choice.^[^ ^82^ ^]^

To quantify this sustainability outlook, we introduce a simple model to estimate the additional mass of electronic materials that could be recovered if Ga‐based LM systems are adopted and effectively recycled. The recoverable mass can be expressed as:

(1) $$\Delta M\approx E\times \left(1-r\right)\times f_{\mathrm{LM}}\times \alpha \times R_{\mathrm{LM}}$$

where E is the global annual e‐waste generation, r is the current formal recycling rate, *f*_LM_ is the fraction of electronic devices in which LM composites could realistically replace conventional conductors, α is the share of conductor mass within those devices, and *R*_LM_ is the achievable recovery yield for LM alloys. Using today's global baseline (E = 62 million t yr^−1^; *r* = 20%), and assuming LM adoption in just 2% of the relevant device segment with a conductor mass fraction of 5% and LM recovery efficiency of 80%,^[^ ^11^, ^19^, ^21^, ^83^ ^]^ the model yields:

(2) $$\Delta M\approx 62\times 10^{6}t\times 0.80\times 0.02\times 0.05\times 0.80\approx 39\;000\;t\;\mathrm{yea}r^{-1}$$

Even under these modest assumptions, nearly 40 kt yr^−1^ could be diverted from landfill or informal disposal. Scaling adoption to 5% of devices raises the estimate to ≈100 kt yr^−1^, highlighting the outsized effect of early design‐for‐recycling choices in fast‐growing segments such as wearables, flexible patches, and smart packaging. These modeled gains are consistent with our empirical LCA results (Figure 1D), which demonstrate category‐wide reductions when LM composites are designed for recovery from the outset. This simplified model is not a precise prediction; it is a transparent, parameterized framework that connects materials and process choices to system‐level outcomes. It shows how even limited penetration of recyclable LM composites can materially improve recovery in small, distributed devices that currently suffer from low capture and minimal resource recovery.

Finally, opportunities exist to adapt strategies from conventional e‐waste recycling to LM‐based soft systems, while recognizing their unique challenges. Conventional methods such as staged disassembly, selective leaching, and density separation remain useful starting points. However, LM‐soft systems present new complexities: the presence of liquid phases embedded in polymers, the stabilizing oxide skin on LM surfaces, intermetallic formation in biphasic inks, and the distinction between thermoplastic and cross‐linked elastomer matrices. Thermoplastic elastomers (e.g., SIS, SEBS, TPU) offer solvent‐ or melt‐based reclamation routes with relatively low cost, whereas cross‐linked systems (e.g., PDMS, Ecoflex) may require chemical depolymerization or tailored solvents. Recent studies demonstrate recovery of Ga and In from LM‐composites using deep eutectic solvents and selective separation methods.^[^ ^20^, ^83^ ^]^ Addressing these unique features early on will improve compatibility with evolving recycling infrastructures. Taken together, these industrial, economic, and recycling perspectives position the 4R framework not as a reaction to an already massive e‐waste problem from soft electronics, but as a proactive approach: guiding design choices today to avoid the environmental pitfalls observed with rigid devices, while aligning with the evolving industrialization roadmap of LM printed electronics.

## 3 State of the Art in Soft Electronics and Sustainable E‐Waste Management

Unlike earlier reviews that focus primarily on electrical and mechanical performance, this section emphasizes how fabrication strategies influence the sustainability of LM–based systems, particularly with respect to recyclability, repairability, and end‐of‐life management. At present, global electronic waste (e‐waste) streams are dominated by devices built on rigid substrates and glassy polymers, most notably Flame Retardant‐4 (FR‐4) epoxy/glass fiber–reinforced printed circuit boards (PCBs) and hard polymer housings. By comparison, soft and stretchable devices contribute only a small fraction of total e‐waste by mass; however, they represent one of the fastest‐growing categories in terms of unit numbers, driven largely by consumer wearables, disposable health‐monitoring patches, and emerging smart packaging.^[^ ^63^ ^]^ Their small size, short life cycles, and wide dispersal often lead to improper disposal, underscoring the need for design‐for‐recovery strategies. Approaches such as clear labeling, take‐back readiness, and LM–polymer formulations compatible with low‐footprint separation can substantially improve recovery rates, even if their mass contribution remains modest. Within this framework, soft electronic systems are most realistically positioned not as replacements for conventional PCBs, but as complementary components in hybrid architectures—where rigid “islands” for chips and power modules are interconnected by LM‐based conductors and sensors.

At the same time, e‐waste management as a whole is undergoing a paradigm shift toward sustainability, driven by the limitations of traditional recycling methods such as mechanical shredding, hydrometallurgical processes, and pyrometallurgical methods. These processes remain energy‐intensive, pollutive, and poorly suited for recovering value from emerging composite systems.^[^ ^84^ ^]^ Soft electronics offer an alternative design trajectory: by leveraging stretchability, conformability, and mechanical resilience, they enable applications ranging from wearable sensors and soft robotics to electronic skin and transient biomedical devices.^[^ ^18^, ^85^ ^]^ Their sustainability contribution, however, depends not only on enabling new functionalities but also on aligning material and structural choices with the 4R principle: Recyclable, Repairable, Renewable, and Resilient.

Soft electronics for highly flexible and stretchable circuitry represent a broad class of innovative solutions that, if designed appropriately, have the potential to reduce the impact of e‐waste. The emergence of soft electronics represents a transformative leap in how electronic systems are designed, manufactured, and deployed. Unlike traditional rigid electronics, soft electronics are characterized by their inherent stretchability, conformability, and mechanical resilience, making them ideally suited for next‐generation applications such as wearable sensors, soft robotics, electronic skin, and transient, non‐clinical at‐home health‐monitoring devices. These technologies are not only enabling new functionalities but are also reconfiguring user‐device interactions by integrating electronics into the human body and everyday objects. Crucially, the sustainability impact of these systems depends not only on their functionality but also on whether their materials and architectures can meet the requirements of the 4R principle (Recyclable, Repairable, Renewable, Resilient).

Despite these promising developments, the shift toward soft and flexible systems has not been accompanied by a proportional advancement in end‐of‐life management strategies. The prevailing approach in electronics—focused on performance optimization and miniaturization—continues to rely on complex material systems, often composed of multiple, inseparable layers of metals, polymers, and semiconductors. While some flexible electronics adopt low‐temperature processing and alternative substrates to enable new form factors, their material choices are rarely guided by principles of environmental sustainability or circular economy design. Moreover, the explosive growth in disposable and short‐lifecycle electronics—such as sensorized wearables, disposable electronic patches, and smart packaging—has significantly exacerbated the e‐waste challenge. These devices are often designed without consideration for disassembly, component recovery, or biodegradability, complicating their recycling and contributing to the already overwhelming burden of electronic waste. Conventional recycling techniques, such as pyrometallurgy and hydrometallurgy, are energy‐intensive, pollutive, and largely incompatible with emerging soft systems. As such, these methods fall short in recovering materials from soft and heterogeneous composites, particularly when rare or non‐precious metals such as gallium or indium are involved. Furthermore, “soft electronics” are not separately tracked in most public e‐waste statistics and are typically embedded within “small equipment/IT”; this under‐reporting reinforces the need for device‐level design cues (standardized labels) and product‐level take‐back programs to improve capture.

**Figure 2:**
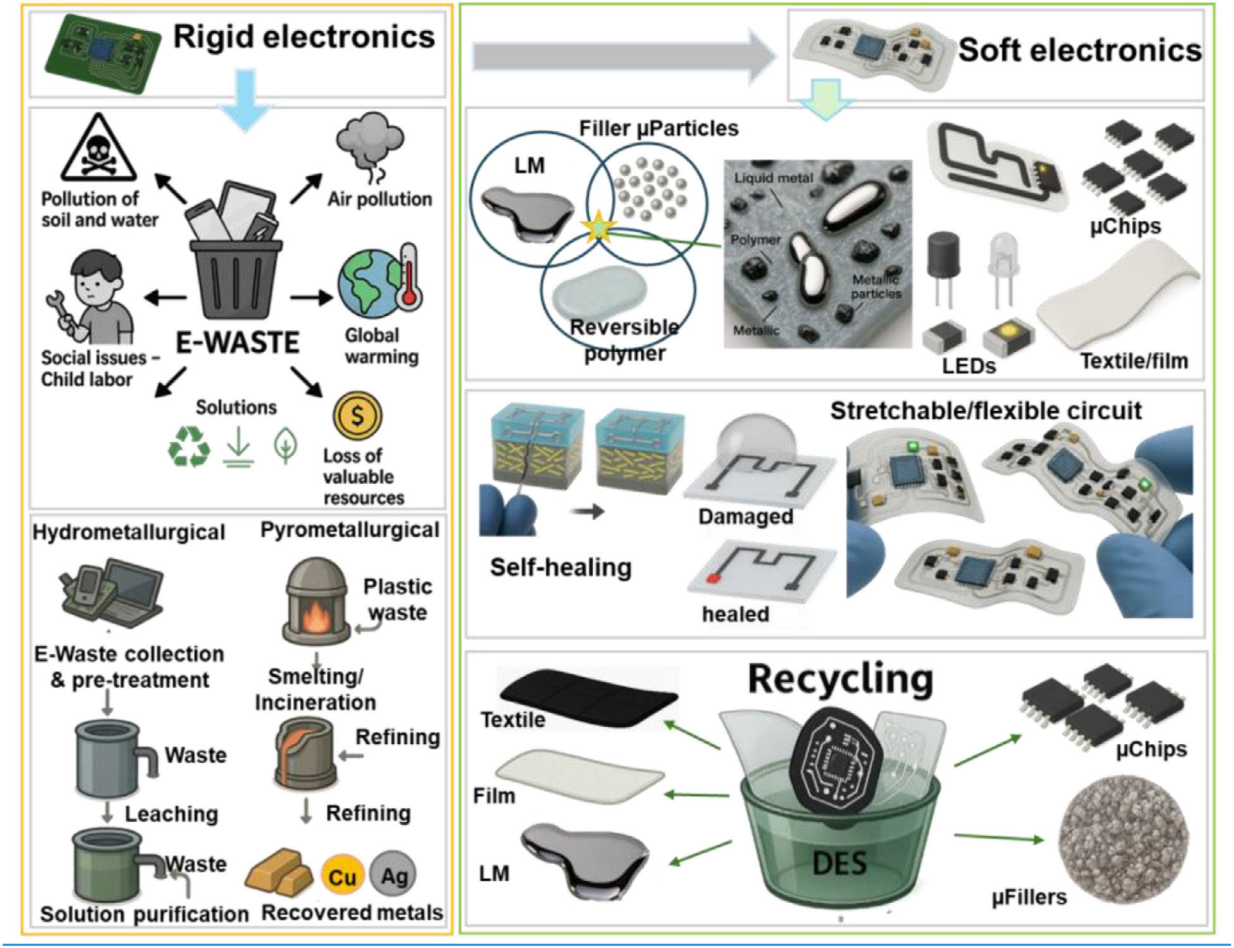
Schematic representation illustrating the transition from rigid to soft electronics by developing resilient, repairable, renewable, and recyclable (4R) liquid metal (LM) composites as a core strategy for E‐waste reduction. Here, “µ” denotes the micron scale—referring to particle size in µFillers and µParticles, and to device dimensions in µChips.

Amid this backdrop, LM‐based composites have gained increasing attention for their unique convergence of electrical conductivity, mechanical compliance, and opportunities for environmentally responsible recovery. Gallium‐based alloys, such as eutectic gallium–indium (EGaIn), offer a rare combination of fluidic deformability and metallic functionality, making them ideal candidates for stretchable circuitry, reconfigurable antennas, and self‐healing sensors (**Figure** **2**). Unlike rigid interconnects, LM traces can undergo extreme deformation without loss of conductivity, enabling truly stretchable and reconfigurable electronics. Importantly, LM composites also offer a pathway toward closed‐loop sustainability—a critical advancement over current soft electronic materials. Gallium and its alloys are not only nontoxic and low‐melting but can also be reclaimed using non‐destructive and environmentally friendly methods, including solvent‐based extraction (e.g., DESs, electrochemical recovery, and selective wet etching. These techniques enable the recovery of LM without destroying the polymeric or elastic substrates, allowing for the reuse of multiple components and materials. However, realizing this potential requires integrating recyclability into the design phase of LM‐based devices—selecting compatible substrates, optimizing composite formulations, and developing scalable, low‐impact recovery strategies. Hybrid devices, in particular, can benefit from LM–polymer systems that avoid intermetallic‐forming fillers and support oxide‐aware release chemistries, thereby improving disassembly and recovery at end‐of‐life.

Yet, material recyclability alone is insufficient. Because most electronic products will continue to combine rigid and soft components, sustainability efforts must also address the mechanical mismatch inherent to hybrid systems. Three complementary strategies can support this transition: device‐level strain management through neutral‐plane layouts and chip thinning;^[^ ^64^, ^86^ ^]^ interface‐level compliance using soft underfills, graded‐modulus interlayers, and debond‐on‐demand adhesives;^[^ ^87^, ^88^, ^89^ ^]^ and interconnect‐level resilience via LM traces that accommodate strain through fluidic flow. Together, these approaches not only enhance mechanical robustness but also improve prospects for disassembly and reuse. Thus, addressing mechanical mismatch is not limited to soft substrates and interconnects, but also requires adapting rigid components themselves—for instance, through chip thinning, neutral‐plane placement, or compliant interlayers—to enable robust and sustainable hybrid integration.

Building on these integration strategies, recent advances in LM‐based ink formulations, surface‐engineered particles, and LM droplet networks embedded in soft polymers have further expanded the design space. Innovations such as thermoplastic elastomers for recyclable substrates, bioresorbable polymers for transient electronics, and self‐healing polymers for extended device lifespans are now being explored to align material performance with sustainability. These developments are reshaping how soft electronics are conceived—not just to function, but to last, heal, and return to the material cycle as part of a circular economy. Taken together, these insights point to an urgent and compelling need to reframe soft electronic systems through the lens of sustainability. Taken together, these developments highlight the need to evaluate LM‐based composites through the lens of sustainability. This review builds on recent progress to assess material choices, integration strategies, recovery methods, and application contexts that define the state of the art. By embedding the 4R principle into design and deployment, LM‐based soft electronics can contribute to a new generation of eco‐conscious devices that reduce e‐waste and promote circularity in the materials economy.

## 4 Significant Impact of Material Selection and Composite Fabrication in Soft Circuits on LM Extraction

The rapid development of electronic devices has contributed to a substantial increase in electronic waste, much of which stems from the use of rigid, non‐degradable, and non‐recyclable materials. The emergence of soft and stretchable electronics presents an opportunity to address this challenge by moving away from conventional materials to materials that are more eco‐friendly and sustainable. One promising approach involves the use of stretchable polymer composites, where an insulating or flexible polymer matrix (X‐Polymer) is integrated with a conductive filler (X) to achieve both electrical functionality and mechanical compliance. To make these soft composites more sustainable, the polymer matrix can be selected from biodegradable or bioderived materials, and the conductive filler can be limited to materials that can be easily recycled.

Stretchable electronics have traditionally been fabricated by mixing or embedding conductive rigid particles, such as carbon allotropes, silver, copper, and others, within a polymer matrix. Conductivity is achieved through either the interconnection of filler particles or by using a conductive polymer matrix.^[^ ^90^, ^91^, ^92^, ^93^ ^]^ Despite their wide range of applications in diverse fields, due to their stretchability and conductivity,^[^ ^92^, ^94^, ^95^, ^96^ ^]^ they face several limitations. Flexible and stretchable power sources for wearable electronics face notable challenges due to material limitations. The elastic moduli of traditional rigid fillers are generally incompatible with those of flexible polymer matrices, significantly impacting the processability and mechanical flexibility of polymer composites (PCs).^[^ ^97^ ^]^ Additionally, these approaches often exhibit critical drawbacks, such as low electrical conductivity—typically three orders of magnitude lower than that of metals—or poor electromechanical properties. A key issue is the increase in electrical resistance under mechanical strain, resulting from the loss of interconnections between rigid particles, which reduces conductivity.^[^ ^98^, ^99^, ^100^ ^]^ In fact, the conductive networks of rigid fillers degrade under strain, leading to significant increases in electrical resistance (*R*) beyond the initial value (*R*_0_). This behavior often exceeds the *R*/*R*_0_ = λ^2^ scaling predicted by Pouillet's Law, where λ represents the stretch ratio. Additionally, rigid fillers increase stiffness and reduce strain limits as their concentration increases, highlighting the incompatibility of the elastic responses between rigid fillers and the soft polymer matrix under mechanical loading.^[^ ^101^ ^]^ An alternative approach employs micro‐ and nanoscale conductive elements with thin geometries, such as serpentine and wavy patterns, which enable stretchable functionality by flexing or twisting on prestrained elastomer substrates.^[^ ^102^, ^103^, ^104^ ^]^ Although these deterministic architectures provide a certain level of stretchability, they are constrained by specific geometric patterns, such as prebuckled waves or planar serpentines, and their deformability is limited to predefined directions.

Another increasingly popular strategy for achieving stretchable circuit functionality is the use of LM alloys. Bulk LM and LM‐based composite inks, formulated from room‐temperature metals such as Ga, EGaIn, and EGaInSn, combine excellent electrical, mechanical, and thermal properties with low toxicity, making them strong candidates for flexible and stretchable electronics.^[^ ^19^, ^75^, ^105^, ^106^ ^]^ Beyond performance, LM alloys also provide sustainability advantages compared to conventional or toxic metallic alloys: their low vapor pressure and chemical stability enable safer handling, their liquid state allows straightforward recovery and recycling, and their intrinsic self‐healing ability minimizes material loss during operation.^[^ ^67^, ^107^, ^108^ ^]^

**Figure 3:**
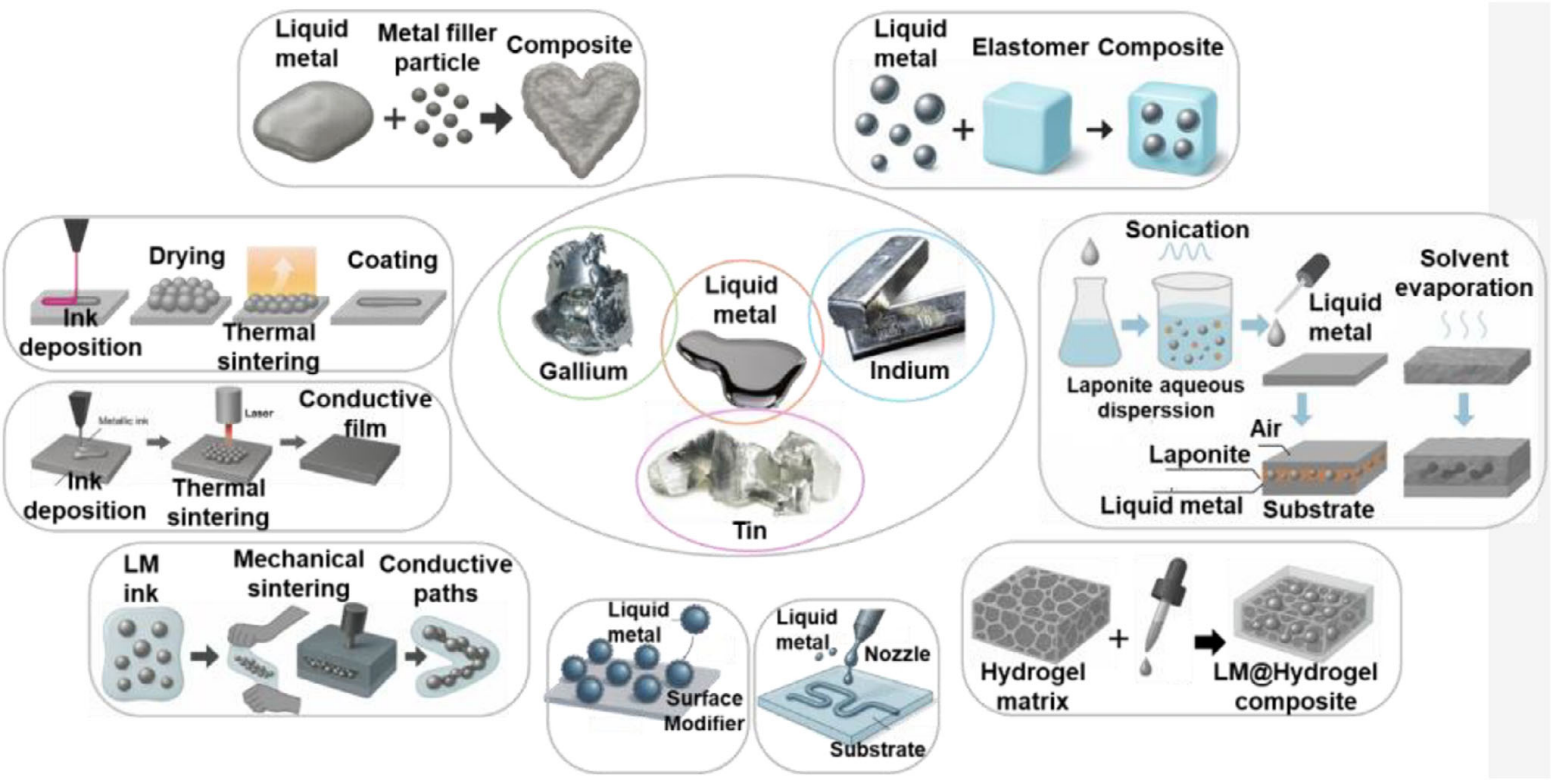
LM‐based ink families and processing routes; 4R mapping is provided in Section 3.

Material and fabrication choices should therefore be evaluated not only for performance but also through the lens of sustainability. In the following subsections, each LM composite family is considered within the 4R framework: recyclability through mild chemistries, repairability and reconfigurability to extend lifetime, incorporation of renewable or eco‐friendly components, and resilience under repeated cycling without loss of recoverability. This perspective ensures that fabrication details are directly linked to sustainability outcomes. Accordingly, we introduce various LM‐based composite inks (**Figure** **3**) as promising pathways for next‐generation soft electronic applications. Their formulations, descriptions, and potential uses in sustainable, biocompatible, and recyclable soft electronics are summarized in **Table** **1**.

**Table 1:** Eutectic Gallium‐Indium (EGaIn) LM and LM‐based composite inks with compositions, key properties, and applications in soft electronics.

Type	Examples	Purpose/Advantage	Applications	Refs.
Eutectic Gallium‐Indium (EGaIn) LM	EGaIn, Galinstan	Non‐toxic, Being liquid at room temperature, High thermal and electrical conductivity, Low vapor pressure	Thermometers, Cooling systems, Soft robotics	[67, 108, 109, 110]
Biphasic LM–X, (X: Cu, Ag, Ni, Fe,	LM + conductive fillers (e.g., Ag, Cu, CNTs, graphene)	Enhances conductivity without needing sintering	Flexible sensors, EMI shielding	[111, 112, 113, 114, 115, 116, 117, 118, 119, 120]
LM–Elastomer Composites	LM droplets + stretchable polymer matrix (e.g., PDMS, Ecoflex, SIS)	Stretchability, softness, and conformability	Wearables sensors, Soft robotics, etc.	[40, 121]
Mechanically Sintered LM Inks	LM droplets in a soft matrix, requiring strain‐induced rupture	Rapid conductivity upon deformation	Strain sensors and stretchable conductors	[122]
Thermally Sintered LM NP Inks	LM nanoparticles (NPs) sintered via heat/laser	Coalescence of small NPs, High‐resolution patterning	Printed electronics, and flexible PCBs	[54, 55]
Polymer‐Functionalized LM NPs	LM NPs with surface ligands (e.g., 11‐phosphonoundecyl acrylate, PEG)	Stability in solvents, Printable nanoparticle inks	Flexible circuits, and electronic textiles	[123, 124, 125]
Recyclable / Transient LM Inks	LM + water‐soluble matrix (e.g., PVA, PEG)	Enables metal recovery via water/NaOH	Sustainable electronics, Transient circuits	[126]
Biphasic LM–X–Polymer Inks	LM + conductive fillers (e.g., Ag, Cu, CNTs, graphene) + polymer matrix	Enhances conductivity without needing sintering	Flexible sensors, EMI shielding	[62, 127, 128]
Self‐Sintering LM Inks	LM + nano/micro additives (e.g., Laponite, fumed silica)	Capillary‐induced fusion, No external sintering is needed	Substrate‐friendly printed circuits	[129, 130]
LM–Hydrogel Inks	LM + hydrogel matrix (e.g., alginate, PAAm)	Biocompatibility, Dual‐conductivity (ionic/electronic)	Bioelectronic and implantable circuits	[131, 132, 133]
LM–Foam Composites	LM impregnated in porous scaffolds (e.g., sugar‐templated structures)	High conductivity + mechanical damping	Stretchable energy storage systems, Sensors, e.g., pressure, temperature, etc.	[134, 135, 136]
Magnetic LM composite ink	Magnetic Filler + LM	High conductivity, Magnetic responsiveness, Reconfigurable circuits, Self‐healing, Recyclability, Multi‐functionality, Remote control, Rewritability, Soft substrate compatibility	Magnetic switcher, EMI shielding, Magnetic hyperthermia, Self‐healing electronics	[19, 137, 138, 139]
Core–Shell LM Microcapsule Inks	LM droplets coated with a shell (e.g., SiO_2_, oxide, polymer)	Stability, Controlled release via rupture	Self‐healing electronics, Reactive printing	[140, 141]
LM–Solvent‐Free Inks	Pure LM or LM + micro/nanofillers without solvent	Eco‐friendly, Residue‐free conductive patterns, Easily recyclable	Eco‐electronics, Thermal interface materials (TIMs)	[142, 143, 144, 145]
LM–Thermochromic Composite Inks	LM + thermochromic pigments or responsive polymers	Color change with temperature for sensing	Thermal sensors, Smart wearables	[146]

**Note**: Recyclability notes related to Table 1: entries 2 and 8 (biphasic LM–X) can face intermetallic‐related barriers (Ag/Cu); entries 3 and 10 underscore that matrix class controls recovery (thermoplastics → solvent/melt reprocessing; cross‐linked elastomers → chemical liberation; hydrogels/water‐soluble → aqueous/alkali release); entries 9 and 14 highlight low‐energy or solvent‐free processing that supports renewable fabrication; entry 7 explicitly targets transient/recyclable systems with benign LM separation.

Following Figure 3, we clarify the recyclability and 4R implications of each depicted route. For LM–polymer emulsions (LMEEs), thermoplastic matrices (SIS/SEBS/TPU) enable solvent wash or melt reprocessing for LM retrieval and polymer reuse, whereas cross‐linked PDMS/Ecoflex typically require chemical LM liberation (alkali/acid or selective etchants). For biphasic LM–X pastes (Ag/Cu/Ni/CNT/graphene), conductivity gains are balanced against intermetallic formation (Ag/Cu) that can hinder LM reclamation, motivating selective etching or DES/ionic‐liquid extraction. Self‐sintering/clay‐assisted routes (e.g., laponite) reduce energy input during fabrication and can be redispersed in water/alkali to facilitate LM release. Digital/laser/thermal printing benefits from sinter‐free/low‐temperature processing, preserving LM fluidity for later rework and disassembly. Bulk LM in microchannels/hydrogels supports mechanical extrusion or aqueous swelling/release from water‐soluble hosts. Finally, stencil/transfer/contact deposition favors non‐reactive interfaces to ease removability, while reactive Ag/Au seed layers improve wetting but may increase recycling complexity due to alloying.

### 4.1 Gallium‐Based LM Alloys (EGaIn and Galinstan) for Sustainable Soft Electronics

Gallium‐based LM alloys, such as eutectic Ga–In (EGaIn; 75% Ga, 25% In) and Ga–In–Sn (Galinstan; 68% Ga, 22% In, 10% Sn), combine high electrical conductivity (3.4 × 10⁶ S·m^−1^), low melting points (15 °C for EGaIn, −19 °C for Galinstan), low viscosity (≈2 mPa·s), high thermal conductivity (20–30 W⋅m^−1^⋅K^−1^), and negligible vapor pressure with relatively low toxicity.^[^ ^147^, ^148^ ^]^ Their metallic conductivity and fluidic nature make them inherently stretchable, enabling soft circuits with performance far beyond conductive polymers or rigid fillers. From a sustainability perspective, Ga‐based LMs align naturally with the 4R framework. Their liquid phase supports low‐energy fabrication, reconfiguration, and end‐of‐life recovery. Native oxide skins (≈1–3 nm) aid patterning and shape retention, yet can be removed using mild chemistries to enable recyclability without high‐temperature processing. Self‐healing and reconfigurability extend device lifetimes, while resilience under repeated cycling ensures durability.^[^ ^83^ ^]^ Compared to toxic LMs such as mercury, gallium alloys offer a far safer, nonvolatile alternative with lower environmental risk.

**Figure 4:**
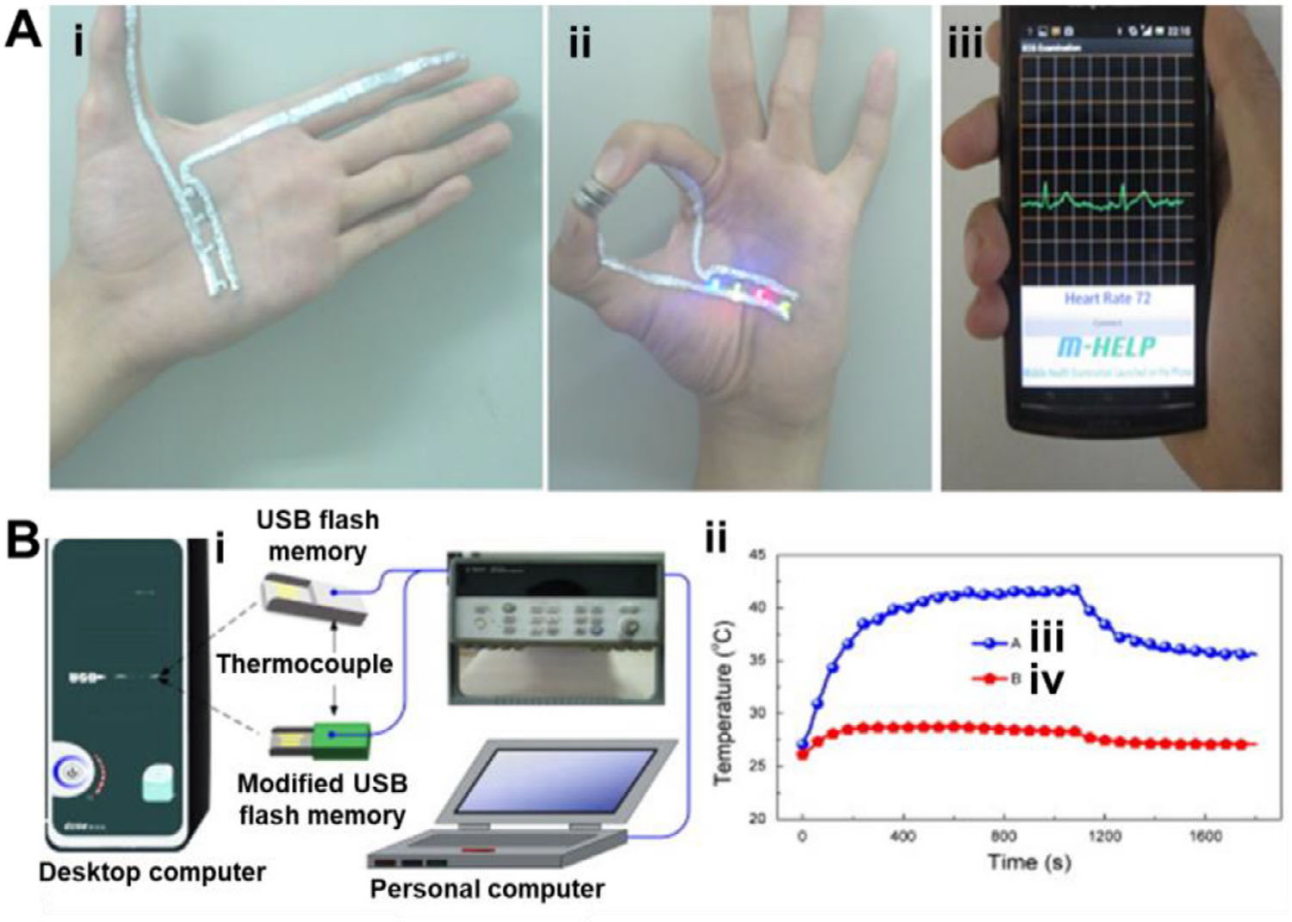
A) The skin circuit is drawn in the palm. i) The basic circuit drawn in the palm; ii) The circuit works when the batteries are gripped and the hand forms a gesture of “OK”, iii) The ECG monitoring displayed on the mobile phone. Reproduced with permission,^[^ ^108^ ^]^ Copyright 2013, PLOS ONE. B) Gallium PCM‐based thermal management of USB flash memory: i) Schematic of the experimental setup. ii) Test results show Curve, iii) for the original USB and Curve, iv) for the USB with gallium PCM, which improved thermal regulation during a 3.6 GB data transfer at ambient temperature. Reproduced with permission,^[^ ^150^ ^]^ Copyright 2013, Elsevier.

Applications already demonstrate these advantages. **Table** **2** highlights LM‐based inks across various fields, mapping their contributions to the 4R framework. For instance, Ga‐based LM inks have been printed as bioelectrodes for ECG monitoring, achieving conductivity of 44.1 µΩ·cm and recording high‐quality signals in both rabbits and humans, while also enabling on‐skin circuit drawing for powering LEDs (**Figure** **4**A).^[^ ^108^ ^]^ As phase‐change materials, gallium effectively dissipates heat in USB devices, maintaining temperatures below 29 °C for over 18 min (Figure 4B). LM marbles and printing methods further showcase recyclability and repairability, as LM droplets can be split, merged, and repurposed for new designs.^[^ ^149^ ^]^ Despite these advantages, and the relatively simple recovery of Ga‐based alloys from waste composites or printed circuits—achieved by removing their oxide layer in mild acidic or basic media (as discussed in Section 7)—several challenges remain in fully realizing 4R goals. Poor adhesion to substrates often causes delamination, while the intrinsic smearing of liquid metals necessitates encapsulation, complicating both device stability and end‐of‐life recovery. To overcome these issues, approaches such as alloying with other metals or embedding LM within polymer matrices have been developed, as will be discussed in the following sections.

**Table 2:** Ga‐based liquid metal systems (EGaIn, Galinstan) and their potential contributions to the 4R principle.

Material (alloy composition)	Application	Results	Sustainability	Refs.
EGaIn (Ga–In eutectic, ≈75.5% Ga, 24.5% In)	Wearable bioelectronic interfaces.	Stable, skin‐safe, and reusable LM ECG electrodes.	Binder‐free, recyclable LM; eliminates conductive gels; reduces disposable waste from single‐use electrodes; however, skin contact requires safety considerations and LM oxidation control	[108]
GaIn_10_ alloy (Ga 90 wt%, In 10 wt%)	Flexible circuits.	Conductive LM lines with stable performance on flexible substrates.	Room‐temperature process; avoids sintering; broad substrate compatibility; simpler tools (pens/brushes); oxidation control needed; durability & swelling under bending/cover films required	[67]
Galinstan (Ga–In–Sn; composition not specified)	Demo ultra‐high frequency (UHF) RFID tag.	200 µm lines / 100 µm gaps; near‐linear resistance to 60% strain; LED unchanged after 1000 cycles (0–60%); RFID read range ≈14 m.	Hg‐free Ga‐based LM; parallel printing (lower labor than injection)	[107]
Galinstan (Ga–In–Sn 67.5:22.5:10 wt%)	Pressure/touch sensor electrode	Capacitance increased ≈5% at 0.07 MPa and ≈15% at 0.18 MPa; proximity detection ≈10 cm; reversible response.	Ga‐based, non‐toxic; reusable operation; PET/PDMS not biodegradable → end‐of‐life separation needed.	[151]
Galinstan (Ga–In–Sn 68.5:21.5:10 wt%)	Actuator / microdevice	Poor MEMS wetting from high surface tension; EWOD improves spreading; requires ultra‐low O_2_.	Non‐toxic Hg substitute; inert/sealed handling needed to limit oxidation (process overhead).	[152]
EGaIn (Ga–In eutectic) and Galinstan (Ga–In–Sn)	Stretchable & reconfigurable electronics.	Printed LM circuits remained conductive under stretch; Galinstan transfer limited by oxide skin.	Hg‐free LMs; µCP reduces molds/masks; PDMS/Ecoflex not biodegradable → EoL separation needed.	[153]
Galinstan (Ga–In–Sn 68.5:21.5:10 wt%)	Triboelectric nanogenerator (TENG) electrode.	20–50 µm features; large‐area transfer; conductivity ≈1.93×10⁶ S·m^−1^; TENG ≈75–100 V.	Maskless/simple transfer (less process chemistry); PDMS not biodegradable → EoL separation required.	[154]
EGaIn (Ga–In 75.5:24.5 wt%)	Stretchable printed circuits.	≈60% stretchability; no leakage after 100 strain cycles; LED integration functional.	Hg‐free; uses less LM than filled channels; PDMS not biodegradable → LM–polymer separation at EoL.	[155]
EGaIn (Ga–In 75.5:24.5 wt%) and Galinstan (Ga–In–Sn 68.5:21.5:10 wt%)	Stretchable conductors, sensors, antennas, and microfluidics.	Near‐RT liquids; oxide skin aids patterning; micro/nanoscale features achievable by multiple routes.	Hg‐free; oxide management adds steps; EoL requires LM–polymer separation.	[156]
Galinstan (Ga–In–Sn; composition not stated)	Capacitor & resistive strain sensor.	High‐resolution LM features enabled capacitors and reversible strain sensors.	Reusable electroplated stencils (lower waste); Ga‐based; PDMS/Ecoflex EoL separation needed.	[157]
Galinstan (Ga–In–Sn 68.5:21.5:10 wt%) and EGaIn (Ga–In 75.5:24.5 wt%)	Solar‐blind photodetector.	≈20 µm features; Galinstan remains conductive to ≈130% strain at −10 °C; EGaIn loses conductivity above ≈110% at −10 °C; UV response present.	Ga‐based, Hg‐free; sub‐zero operability extends lifetime; PDMS not biodegradable → EoL separation needed.	[158]
EGaIn (Ga–In 75:25 wt%)	Stretchable LM‐based sensor circuits.	Low‐resistance LM lines maintained conductivity under high stretch and cycling.	Hg‐free; Cu interfaces scalable; requires NaOH/HCl steps; PDMS end‐of‐life separation needed.	[159]
EGaIn (Ga–In 75:25 wt%) and Galinstan (Ga–In–Sn)	Review—microfluidics and stretchable electronics	Oxide skin enables 3D shaping, adhesion, and reconfigurable LM circuits via direct writing and printing.	Low‐toxicity alternative to Hg; recyclable and reconfigurable; compatible with soft electronics. oxide complicates processing but also enables unique functionalities; recycling requires oxide/LM separation	[160]

^*)^Micro‐Electro‐Mechanical Systems (MEMS); Electrowetting‐on‐Dielectric (EWOD); Electrocardiogram (ECG); Ultra‐High Frequency Radio‐Frequency Identification (UHF RFID) tag; Polyethylene Terephthalate (PET); microcontact printing (µCP); end‐of‐life (EoL); light‐emitting diode (LED).

### 4.2 LM‐Solid Particle Composite Ink/Paste: Toward 4R Soft Electronics

Liquid metal–solid particle (LM–SP) composites/pastes have emerged as a solvent‐free and scalable approach to overcome the processing and stability challenges of pure LMs. While gallium‐based alloys such as EGaIn or Galinstan provide excellent conductivity and fluidity, their poor adhesion, leakage, and patterning difficulties hinder integration into practical devices. By dispersing solid fillers such as carbon black, silver flakes, graphene, or metallic microparticles directly into the LM, these composites gain tunable rheology, enhanced adhesion, and improved printability while maintaining high electrical and thermal performance. From a sustainability perspective, LM–SP composites align with the 4R framework: their recyclability depends on the filler choice, since inert particles enable LM recovery, whereas intermetallic‐forming fillers (e.g., Cu, Ag, Au, Fe, Ni, etc.) complicate separation. Their paste‐like nature also allows repair by refilling or re‐patterning, renewable fillers can reduce environmental footprint, and their resilience under strain ensures long‐term device operation. This positions LM–SP pastes as an attractive platform for sustainable soft electronics.

**Figure 5:**
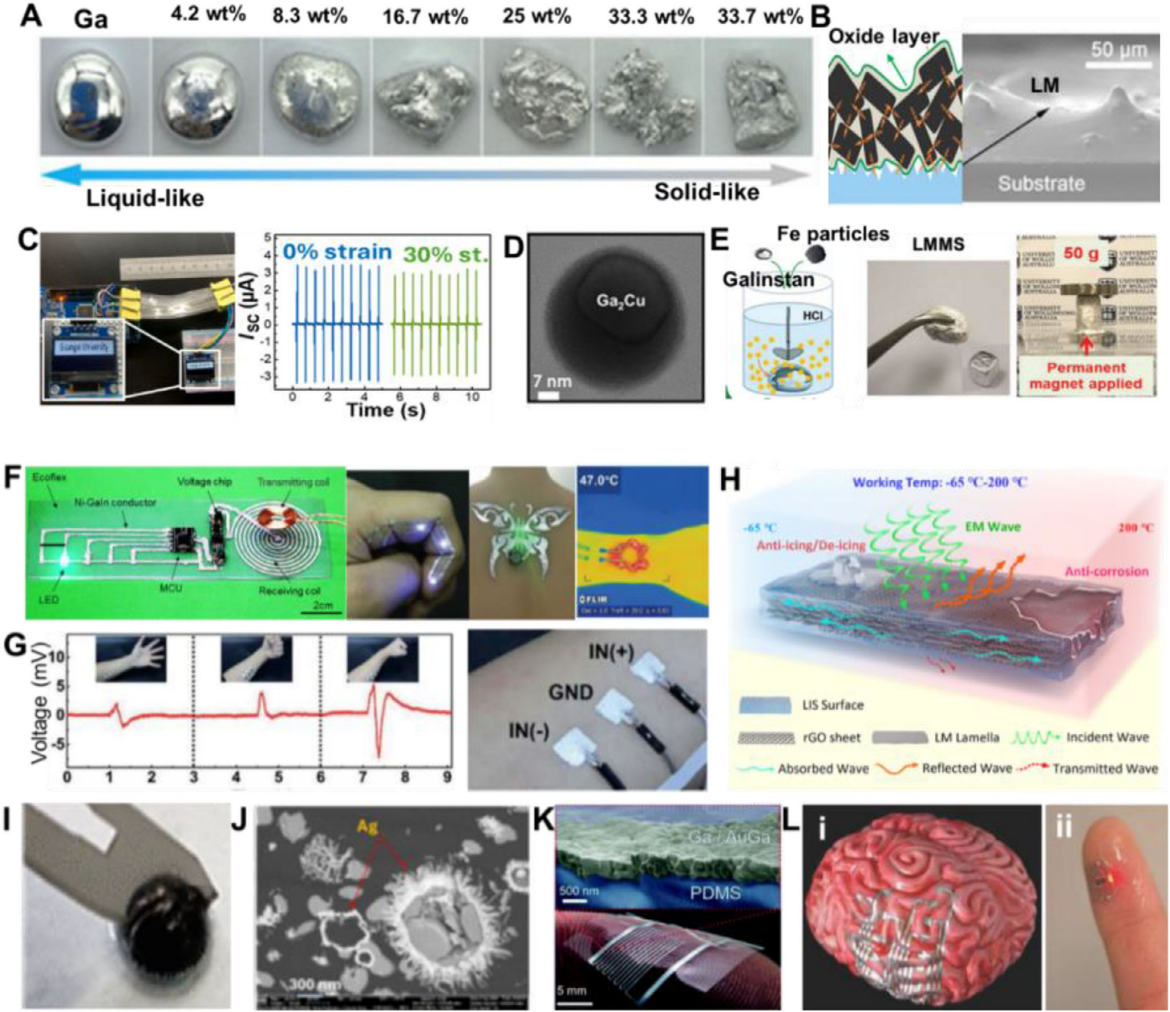
A) The change of LM phase from liquid to slurry, putty, deformable, and powder form by increasing the concentration of metal filler into the LM. Reproduced with permission,^[^ ^175^ ^]^ Copyright 2022, Elsevier. B) A schematic cross‐sectional view of the liquid metal bridges formed between solid particles. Reproduced with permission,^[^ ^117^ ^]^ Copyright 2017, ACS Publications. C) Cu‐EGaIn electrodes ensure stable performance of the TENG under mechanical deformation. Reproduced with permission,^[^ ^119^ ^]^ Copyright 2022, Elsevier. D) TEM images of the LM‐Cu intermetallic compounds. Reproduced with permission,^[^ ^176^ ^]^ Copyright 2024, Wiley. E) Galinstan mixed with Fe particles in HCl forms a muddy slurry (i). The stiffness of the LMMS is enhanced under a magnetic field, supporting a 50 g load without deformation. Upon magnet removal, the tall cylinder collapses (ii). Reproduced with permission,^[^ ^116^ ^]^ Copyright 2018, Wiley. F and G) Ni‐GaIn amalgams are used as a stretchable wireless power transfer device, tattoo, LED, Joule‐heat, temperature, and motional sensor. Reproduced with permission,^[^ ^112^, ^113^ ^]^ Copyright 2018,2019, Wiley. H) Schematic Representation of EMI Shielding Mechanism,^[^ ^177^ ^]^ Copyright 2023, ACS Publications. I) LM particle is coated with Au NPs layer. Reproduced with permission,^[^ ^168^ ^]^ Copyright 2017, ACS. J) SEM image showing the Surface reactivity of the LM with Ag, and making GaAg intermetallic compound over the Ga LM surface. Reproduced with permission,^[^ ^21^ ^]^ Copyright 2024, ACS. K) Sputtered gold (Au) thin films serve as templates to promote alloying and wetting, enabling uniform liquid gallium (Ga) deposition on elastomer substrates. Reproduced with permission,^[^ ^171^ ^]^ Copyright 2016, Wiley. L) Soft, stretchable electronic tattoos made by EGaIn‐assisted sintering of silver nanoparticles. Top: transferred to a fingertip. Bottom: adhered to a textured toy brain. Reproduced with permission,^[^ ^172^ ^]^ Copyright 2018, Wiley‐VCH.

These pastes are prepared by dispersing fillers into the LM via mechanical stirring and surface oxidation, yielding tunable, printable, and conductive inks. As filler content increases, the composite evolves from a slurry to a deformable paste and eventually a powder‐like form (**Figure** **5**A), with higher oxidation enhancing adhesion but reducing fluidity and conductivity due to oxide buildup.^[^ ^117^, ^161^ ^]^ Therefore, maintaining a balance between adhesion and electrical performance requires careful control of the doping ratio.^[^ ^113^ ^]^ One approach to improving performance involves the development of Cu‐LM composite inks, which are created by dispersing copper particles into EGaIn.^[^ ^117^ ^]^ Figure 5B presents a schematic cross‐sectional view of the liquid metal bridges formed between solid particles. These semiliquid/semisolid mixtures exhibit significantly enhanced electrical conductivity (6 × 10⁶ S m^−1^) and thermal conductivity (50 W m^−1^ K^−1^) due to the formation of CuGa_2_ intermetallics. While CuGa_2_ compounds improve thermal conductivity, these composites also display excellent adhesion, self‐healing, and printability, making them suitable for stretchable and reconfigurable electronic systems. Moreover, their rheological properties make them ideal for printing fine electrical circuits, ensuring strong adhesion to the substrates.^[^ ^117^ ^]^ Another study developed a multifunctional, self‐powered system using copper‐integrated EGaIn (Cu‐EGaIn) as stretchable and flexible electrodes. This system includes a triboelectric nanogenerator (TENG) for energy harvesting, a power management module (PMM), micro‐supercapacitors (MSCs) for energy storage, and application circuits, creating a compact and efficient platform for wearable electronics. The Cu‐EGaIn electrodes ensure stable performance of the TENG under mechanical deformation, including stretching and bending (Figure 5C).^[^ ^119^ ^]^

To further enhance the stability of EGaIn‐based LM mixtures, a process was developed that combines the alloy with copper particles in a sodium hydroxide (NaOH) solution under a 5V electric field. This method promotes the internalization of particles and removes surface oxides. However, the introduction of water during this process can cause corrosion, making the intermediate product unstable. To stabilize the mixture, vacuum drying at 60–80 °C for 3 h is required to eliminate absorbed water and yield a stable semi‐liquid or semi‐solid material.^[^ ^117^, ^118^ ^]^ While stable, high thermal conductivity materials are preferable, the presence of intermetallic compounds such as CuGa_2_ (Figure 5D)—consistent with the Cu–Ga binary phase diagram—poses challenges for recycling at room temperature.^[^ ^117^ ^]^ Furthermore, the formation of CuGa_2_ intermetallic alloys can stiffen LM‐Cu mixtures, resulting in the formation of large tetragonal blocks,^[^ ^117^ ^]^ which may hinder their performance in specific applications.^[^ ^115^, ^118^ ^]^

An alternative approach involves using non‐alloying LM‐particle mixtures with tungsten microparticles, which enhance thermal conductivity without forming intermetallic compounds or embrittling the LM.^[^ ^162^, ^163^ ^]^ Tungsten, with a thermal conductivity of 173 W m^−1^ K^−1^, improves both thermal conductivity and rheological properties while remaining chemically inert toward LMs.^[^ ^114^ ^]^ However, the recyclability and recovery of the LM in such mixtures have not yet been reported. Moreover, the incorporation of magnetic particles into LM systems has significantly expanded their potential for magneto‐responsive applications. For instance, dissolving gadolinium (Gd) into Galinstan resulted in the in‐situ precipitation of Gd nanoparticles (≈2%), imparting bulk magnetic responsiveness. This property is particularly useful for applications like magnetocaloric refrigeration.^[^ ^111^ ^]^ In a separate study, Fe microparticles were mixed with bulk EGaIn in acidic media, resulting in a foam‐like composite that exhibited a strong response to external magnetic fields.^[^ ^164^ ^]^

These Galinstan‐Fe systems allowed stiffness modulation across three orders of magnitude (ranging from kPa to MPa), which is advantageous for developing reconfigurable soft bioelectrodes suitable for implantable or adaptive interfaces (Figure 5E).^[^ ^116^ ^]^ Similarly, incorporating nickel (Ni) microparticles into EGaIn to form biphasic composites (EGaIn–Ni) improved wetting behavior and adhesion to porous substrates like paper.^[^ ^165^ ^]^ This property enabled the direct writing of flexible electronic circuits. Additionally, a technique using magnetically guided direct writing of LM droplets containing Ni particles was developed. In this process, a magnetic field is applied beneath the substrate to aggregate the Ni particles and guide their flow, enabling precise, contactless patterning of conductive tracks on various substrates.^[^ ^166^ ^]^

When embedded in elastomeric matrices like Ecoflex, EGaIn–Ni composites maintained high conductivity (≈1.6 × 10⁶ S·m^−1^) and stretchability, preserving their electrical performance even under mechanical deformation. Furthermore, conductive patterns could be directly transferred onto the human skin, making the composites suitable for epidermal devices such as stretchable wireless power transfer devices, Joule‐heat and temperature sensors, tattoos, leads, and motional sensors (Figures 5F and 5G).^[^ ^112^, ^113^ ^]^ Furthermore, the Ni–EGaIn composite also shows great potential for applications in electromagnetic interference (EMI) shielding and flexible electronics. It is created by simply stirring EGaIn with Ni powder in varying mass ratios (1:1 to 3.5:1), which allows tunable viscosity and magnetic permeability. The resulting non‐Newtonian mixture of EGaIn, Ni particles, gallium oxide, and internal pores demonstrates shear‐thinning behavior, excellent substrate adhesion, and consistent structural uniformity when applied with a brush or roller. The composite exhibited impressive EMI shielding effectiveness, achieving over 75 dB on rigid PVC, and 45–60 dB even under 300% strain on stretchable Ecoflex.^[^ ^120^ ^]^ Figure 5H illustrates the possible EMI shielding mechanisms, namely reflection at the material surface due to impedance mismatch, absorption through dielectric and magnetic losses within the material, and multiple internal reflections facilitated by the interconnected conductive pathways. These properties make the composite a highly promising multifunctional ink for next‐generation wearable and flexible electronics.

Noble metals have also been strategically employed to overcome the high surface tension of LM and improve pattern fidelity in printed conductors.^[^ ^167^, ^168^, ^169^ ^]^ Gallium (Ga) exhibits strong reactivity with metals such as gold (Au) (Figure 5I),^[^ ^168^ ^]^ silver (Ag) (Figure 5J),^[^ ^21^ ^]^ and platinum, facilitating the diffusion of these metals and the formation of intermetallic compounds through reactive wetting.^[^ ^170^ ^]^ Metallic nanoparticles facilitate electron transfer, enhancing catalytic activity.^[^ ^167^ ^]^ This property allows metallic particles to be incorporated into EGaIn, transforming it from a fully liquid state into a semi‐solid or semi‐liquid phase that maintains excellent electrical conductivity and mechanical ductility. This intermediate state enhances the material's patternability, making it suitable for flexible electronic applications. One approach involved using sputtered Au thin films as templates for alloying and wetting, facilitating the deposition of liquid Ga onto elastomer substrates (Figure 5K).^[^ ^171^ ^]^ The Au/Ga interface promoted the formation of an alloy, enabling the creation of a continuous conductive network that would otherwise be unattainable on bare elastomers (Figure 5K –top). This method was particularly useful in the fabrication of flexible devices such as wearable flexion sensors and microheaters (Figure 5K –bottom). Another effective strategy utilized inkjet‐printed Ag nanoparticles as patterned templates for alloying, followed by the casting of eutectic gallium–indium (EGaIn) to form what is known as “electronic tattoos”.^[^ ^172^, ^173^ ^]^ These tattoos are ultrathin, stretchable conductors capable of conforming to skin and irregular 3D surfaces. The resulting LM–Ag composites demonstrated impressive properties, including high conductivity (≈4.8 × 10⁶ S·m^−1^) and large stretchability (up to 120%), alongside a scalable and cost‐effective fabrication process, making them highly suitable for wearable electronics (Figure 5L). These techniques not only improve the material properties of LM composites but also enable the development of flexible and stretchable electronic systems with applications in healthcare, wearables, and skin‐conformable electronics.^[^ ^174^ ^]^

In conclusion, while mixing LM with solid conductive particles improves electromechanical, thermal, and adhesion properties, it also raises significant sustainability challenges. The formation of intermetallic phases like CuGa_2_, InAg_2_ enhances conductivity but hinders recyclability,^[^ ^11^ ^]^ as these compounds are difficult to separate through mild or low‐energy processes. From a 4R perspective, designing LM–particle systems with non‐alloying or inert fillers (e.g., W, BN, CNTs) can preserve recyclability, while functional additives such as Ni or Fe introduce reconfigurability and magnetic responsiveness that extend device lifetimes. Balancing these trade‐offs is critical to ensure that LM–solid composites contribute not only to performance but also to long‐term repairability, resilience, and circular recovery pathways.

### 4.3 LM–Polymer Composites for 4R‐Oriented Soft Electronics

LM–polymer composites integrate the conductivity and fluidity of LMs with the flexibility and resilience of polymers, enabling applications in wearable sensors, soft robotics, and thermal management. Their sustainability depends not only on LM properties but also on polymer choice and composite architecture, which govern recyclability and repairability. While several reviews have summarized LM–polymer composites with emphasis on fabrication strategies and electromechanical performance^[^ ^178^, ^179^ ^]^ or specific applications such as healthcare and microfluidics,^[^ ^180^, ^181^ ^]^ end‐of‐life management remains largely overlooked. Although LM‐based soft electronics are often described as recyclable and reconfigurable, a review dedicated to their role within the 4R framework is still lacking; in the following, we address how recycling and circularity strategies can position LM–polymer composites as a cornerstone for sustainable and next‐generation soft electronics.

#### 4.3.1 Early Developments and Structural Configurations

**Figure 6:**
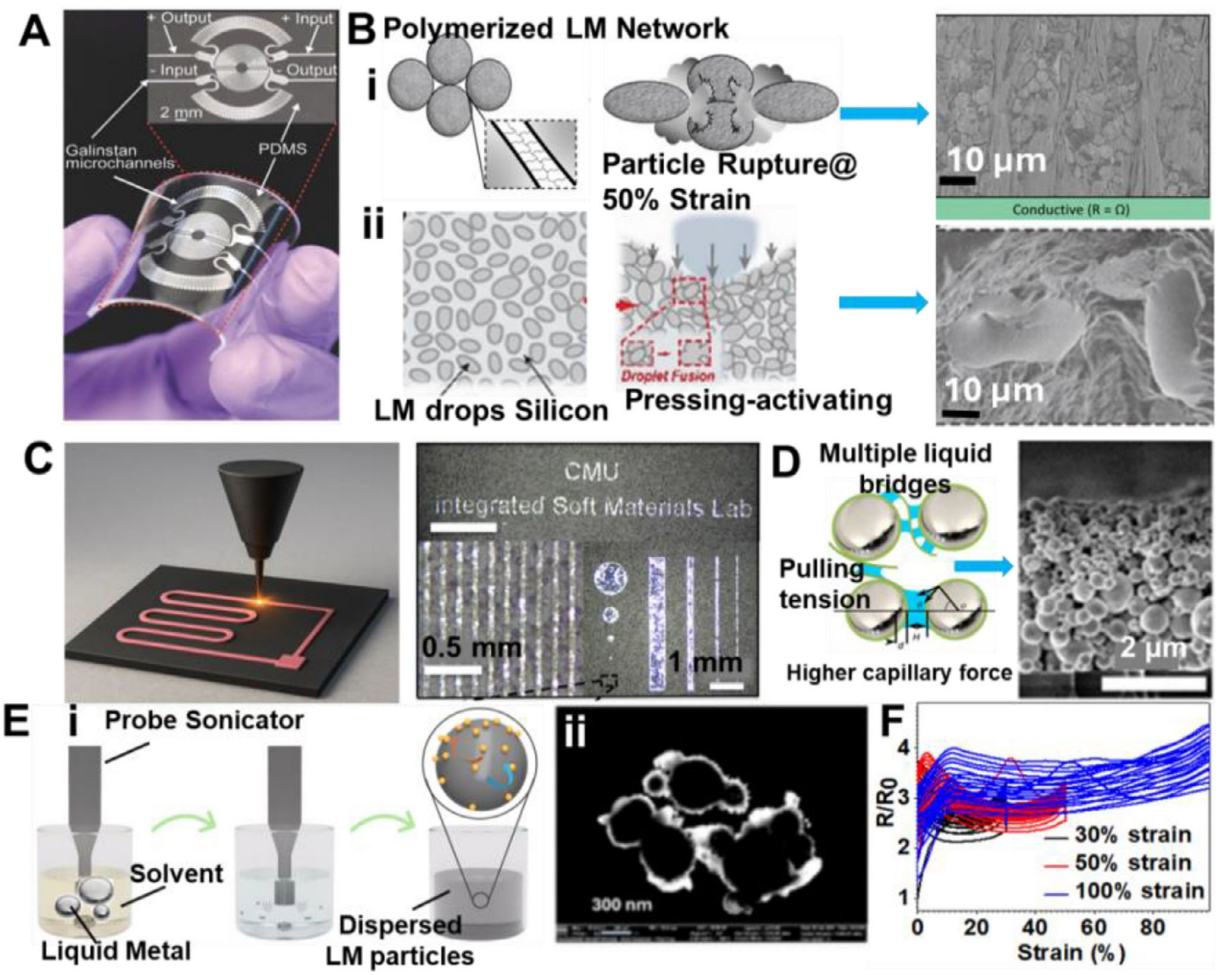
A) LM‐filled microchannels serve as highly stretchable conductive wires and passive circuit elements, enabling flexible and deformable electronic applications. Reproduced with permission,^[^ ^208^ ^]^ Copyright 2017, Wiley. B) LM‐Polymer composite, where its conductivity is obtained by “mechanical sintering”, i) the composite subjected to i) stretch, and ii) pressure. SEM images showing stages of network formation: core–shell LM particles with acrylate‐functionalized oxide shells (left inset), polymerized particles forming cross‐linked networks (inner left), strain‐induced rupture enabling conductivity (inner right), and relaxed networks after compression (outer right). Reproduced with permission,^[^ ^209^ ^]^ Copyright 2019, Wiley. C) LM‐Polymer composite, where its conductivity is obtained by “thermal sintering”, i) the composite subjected to laser sintering and patterning. Reproduced with permission,^[^ ^210^, ^211^ ^]^ Copyright 2017, ACS., Nature. D) Schematic representation of evaporation‐induced sintering of LM droplets with biological nanofibrils. The process involves capillary forces enhanced by biological nanofibers (NFs), localized pulling tension generated by the NFs, and the splitting of droplets into multiple liquid bridges. Deposition of an NF suspension onto a layer of EGaIn droplets (prepared without NFs) initiates evaporation‐driven sintering. Reproduced with permission,^[^ ^212^ ^]^ Copyright 2019, Nature Communications. E) Surface engineering of LM particles via i) probe sonication and galvanic replacement. The resulting Ag@LM particles were incorporated into an SIS polymer matrix, and ii: SEM images of the composite, and F) evaluated through electromechanical measurements. Reproduced with permission,^[^ ^21^ ^]^ Copyright 2024, ACS.

The earliest applications of LMs in elastomeric systems leveraged their inherent conductivity and fluidity to create stretchable electronic components. A foundational strategy involved injecting bulk EGaIn into microfluidic channels embedded within soft elastomers, most commonly polydimethylsiloxane (PDMS). These LM‐filled microchannels functioned as highly stretchable wires and passive circuit elements, enabling the development of deformable electronics (**Figure** **6**A)^[^ ^153^, ^182^ ^]^ such as soft interconnects, artificial skin for tactile sensing,^[^ ^183^ ^]^ deformation sensors, and reconfigurable electronic components.^[^ ^153^ ^]^ Over time, EGaIn‐based microchannels were integrated into diodes, memristors,^[^ ^184^, ^185^ ^]^ and tunable antennas responsive to mechanical deformation or electrochemical modulation.^[^ ^186^, ^187^ ^]^ These early innovations highlighted the potential of bulk LMs systems to combine compliance with robust electrical functionality, laying a foundation for advanced LM–polymer composite designs.

From a sustainability standpoint, however, such early configurations present limitations. PDMS‐based LM microchannels, while foundational for stretchable electronics, pose clear sustainability and recyclability challenges. PDMS is a permanently cross‐linked thermoset, meaning that once cured, it cannot be remelted or dissolved, which makes LM channels embedded in PDMS nearly impossible to separate or recycle at end‐of‐life.^[^ ^147^ ^]^ In addition, the native Ga_2_O_3_ skin that forms on eutectic Ga–In stabilizes the LM within the channels and aids pattern retention, but this same oxide layer can restrict drainage and recovery of the metal when reuse is desired.^[^ ^160^ ^]^ Hybrid LM–PDMS devices often co‐integrate rigid islands such as ICs or LEDs directly into the elastomer, which further complicates separation and disassembly at end‐of‐life.^[^ ^188^ ^]^ Finally, PDMS microchannel fabrication itself—whether by soft lithography or laser micromachining—produces silicone scrap, and LM can be lost during the injection or flushing steps.^[^ ^25^, ^189^ ^]^ Together, these features highlight that while LM–PDMS systems established the field, their closed‐network architecture and oxide‐stabilized channels present barriers to recycling and reuse, making them less compatible with 4R‐oriented design.

#### 4.3.2 Liquid Metal–Polymer Composite (LMPC) Inks

As a response to the sustainability and recyclability limitations of LM–PDMS microchannels, research attention shifted toward LMPC inks. These materials embed LM droplets—such as Ga, EGaIn, or Galinstan—into elastomeric polymer matrices, including PDMS, Ecoflex, TPU, SEBS, SIS, and, more recently, other reprocessable or biodegradable polymers.^[^ ^62^, ^190^ ^]^ The resulting LM‐embedded elastomers (LMEEs) combine metallic conductivity with the inherent fluidity of the LM, both retained at room temperature.^[^ ^62^ ^]^ Unlike closed PDMS channel systems, LMPC inks can be patterned by scalable techniques such as screen printing, inkjet printing, or extrusion,^[^ ^191^ ^]^ which minimizes material waste and facilitates recovery of LM at end‐of‐life. This approach not only enhances structural stability and functional performance but also improves alignment with the 4R framework by enabling designs that are more recyclable, repairable, and reconfigurable for soft and stretchable electronics applications.^[^ ^75^, ^192^ ^]^

Early studies frequently used PDMS and Ecoflex as host matrices, embedding LM droplets to create stretchable and conductive composites. This embedding strategy addressed the long‐standing challenge of balancing functionality and flexibility: micrometer‐sized LM droplets boosted fracture energy by up to 50 times compared to unfilled polymers,^[^ ^193^ ^]^ enabling improved crack resistance. LM–Ecoflex composites, for instance, have been applied in strain sensors and e‐skin, maintaining conductivity under large deformations.^[^ ^194^ ^]^ Despite these advances, LM recovery remains a major limitation, hindering full alignment with the 4R framework. In LM–PDMS composites, recovery is restricted by the non‐recyclable nature of PDMS with permanent covalent crosslinks.^[^ ^195^ ^]^ To overcome this, functionalization strategies have been introduced to impart recyclability and self‐healing through the use of dynamic bonds. For example, PDMS elastomers cross‐linked via dynamic transesterification can be reshaped and recycled at 180 °C while retaining stability; however, challenges include high processing temperatures, thermal aging, and catalyst dependence.^[^ ^58^ ^]^ Similarly, incorporation of aromatic disulfide and multiple hydrogen bonds produced tough PDMS elastomers (tensile strength ≈1.9 MPa, elongation ≈340%), with ≈96% room‐temperature self‐healing and recyclability, but issues of long‐term stability and device reliability persist.^[^ ^196^ ^]^

Thermoplastic elastomers such as TPU, SEBS, and SIS have since been explored as LMPC matrices to enable solvent‐ or melt‐assisted recovery. While more reprocessable than PDMS or Ecoflex, their recyclability often depends on toxic solvents (e.g., toluene, DMF),^[^ ^21^ ^]^ and greener alternatives remain largely experimental.^[^ ^19^ ^]^ Moreover, recovery of LM droplets frequently requires chemical treatments to remove the Ga_2_O_3_ skin, raising concerns over scalability and environmental impact. Thus, balancing durability, functional performance, and end‐of‐life recovery remains a central challenge in advancing LMPC inks toward circular electronics.

The development of LM–polymer composites has advanced from non‐recyclable thermosets (e.g., PDMS), to partially reprocessable thermoplastics (e.g., TPU, SEBS, SIS), and now toward vitrimers that uniquely combine durability with closed‐loop recyclability. Vitrimer‐based LM composites merge the strengths of thermosets and thermoplastics by relying on dynamic covalent networks that undergo bond exchange, thereby providing thermoset‐like strength while enabling thermoplastic‐like repair, reprocessing, and healing.^[^ ^41^ ^]^ When functionalized with LM fillers, these systems yield conductive, multifunctional materials that directly address the 4R goals for sustainable soft electronics. For instance, an EGaIn–vitrimer composite embeds LM microdroplets in a reprocessable network that delivers high conductivity, self‐healing, shape‐memory, and load‐bearing capacity, while its ester‐linked matrix can be depolymerized under alkaline conditions to recover both LM and polymer components. Unlike epoxy–LM systems, this enables reconfigurable circuit boards designed for closed‐loop electronics,^[^ ^41^ ^]^ and subsequent advances with chain‐extended vitrimers have introduced tunable flexibility and toughness, as demonstrated by USB cables that maintained charging and data transfer capabilities while remaining healable and solvent‐recyclable.^[^ ^60^ ^]^

These principles extend beyond mechanical performance to thermal management, where epoxy vitrimers incorporating poly(lipoic acid)‐modified LM droplets function simultaneously as crosslinkers and thermally conductive fillers. Improved dispersion and bonding enhanced both strength and thermal conductivity by over fivefold compared with neat epoxy, and these properties were preserved after reprocessing, with the composites degrading under mild reducing conditions to support sustainable recycling.^[^ ^61^ ^]^ Other strategies focus on processing advantages, such as lignin‐based vitrimers that stabilized LM nano‐dispersions, enabling high‐resolution circuit printing, rapid photothermal repair, and nearly complete recovery of both LM and matrix (≈98%), thereby moving closer to practical closed‐loop reuse.^[^ ^197^ ^]^ At the molecular scale, such progress is grounded in dynamic covalent chemistry (DCC), where dissociative bonds (e.g., Diels–Alder, imine) permit recycling under mild conditions while associative exchanges (e.g., transesterification, disulfide) preserve crosslink density to maintain vitrimer‐like resilience. Hybrid and multi‐bond strategies further balance toughness, conductivity, and degradability, underscoring the importance of renewable feedstocks, catalyst‐free chemistries, and multifunctionality in the design of circular thermosets.^[^ ^198^, ^199^, ^200^ ^]^ The sustainability potential of these systems was demonstrated in a soybean‐oil‐based vitrimer coating with carbon nanofillers, which combined high conductivity, stretchability, sensing, and room‐temperature healing with solvent recyclability and seawater degradability, highlighting opportunities for e‐skins and wearable sensors.^[^ ^201^ ^]^ Collectively, such advances establish LM–vitrimers as a 4R pathway that integrate conductivity, robustness, and multifunctionality with designed disassembly and recovery, transforming soft electronics from potential e‐waste into contributors to circular manufacturing.

#### 4.3.3 Structure–Property–Sustainability Relationships in LM–Polymer Composites

The sustainability of LM–polymer composites (LMPCs) is closely linked to their microstructure, where droplet size, connectivity, interfacial chemistry, and matrix selection simultaneously dictate performance and end‐of‐life recovery. Small LM droplets improve conductivity at low percolation thresholds, but their oxide shells complicate recycling, whereas larger droplets offer easier recollection at the expense of electrical efficiency—illustrating the trade‐off between performance and recyclability.^[^ ^166^ ^]^ Similarly, continuous LM networks provide metal‐like conductivity and resilience under strain, yet their strong percolation hinders disassembly; strategies that promote reversible connectivity or self‐healing can mitigate this limitation by coupling durability with reusability.^[^ ^41^, ^202^ ^]^ Interfacial chemistry further governs sustainability outcomes: while strong bonding between LM droplets and polymers enhances toughness by orders of magnitude,^[^ ^193^ ^]^ it also prevents filler release at end‐of‐life (EoL). By contrast, dynamic or weaker interfaces enable LM recovery but may compromise mechanical robustness, emphasizing the need for balance.^[^ ^203^ ^]^ The host matrix ultimately sets the recyclability ceiling—irreversible cross‐linked elastomers such as PDMS trap LM permanently, reprocessable thermoplastics (TPU, SEBS, SIS) enable partial recovery, and vitrimers uniquely merge thermoset‐like stability with chemical recyclability via bond‐exchange reactions, allowing nearly closed‐loop reuse of both LM and matrix.^[^ ^41^ ^]^ Equally important, solvent selection reshapes composite microstructure by controlling droplet dispersion and interfacial interactions. While conventional solvents such as toluene ensure basic processability, greener solvent systems improve uniformity, conductivity, and mechanical stability while also enabling reversible dissolution and recovery, thereby directly linking processing chemistry to recyclability and the broader 4R framework.^[^ ^19^ ^]^

Recent studies highlight these relationships by demonstrating ≈98% LM recovery from droplet–elastomer composites,^[^ ^41^ ^]^ Diels–Alder‐based polyurethane–LM systems capable of reversible reprocessing and repair,^[^ ^121^ ^]^ and vitrimer‐based LMPCs that combine conductivity, toughness, and healing with closed‐loop recyclability.^[^ ^41^ ^]^ Together, these findings underscore a central tension: microstructural choices that maximize conductivity and toughness—fine droplets, strong interfaces, continuous networks—tend to obstruct disassembly and reuse, while design strategies that enable recovery often reduce resilience or performance. By framing these trade‐offs through a structure–property–sustainability perspective, the field is moving from non‐recyclable thermosets to reprocessable thermoplastics and, most recently, to vitrimers that uniquely align durability with closed‐loop recovery. Establishing this framework will enable systematic optimization of LMPCs, ensuring that materials are not only high‐performing but also consistent with the 4R principles.

Researchers have also explored innovative strategies for developing recyclable LM‐polymer composite inks. One promising approach involves incorporating LM particles into water‐soluble polymers to enhance recyclability. For example, a conductive ink was formulated using poly(vinyl alcohol) (PVA) and LM, enabling the separation and recovery of the metal using a NaOH solution.^[^ ^126^, ^204^ ^]^ Furthermore, researchers have successfully integrated LM with chip components to develop recyclable electronic devices, recovering the metal through chemical treatments.^[^ ^205^ ^]^ Despite these advances, effectively separating and recovering soft LMs from substrates or encapsulated structures remains challenging. This often requires complex dissolution processes and chemical treatments.^[^ ^206^ ^]^ Therefore, to fully realize the potential of flexible and recyclable electronics, it is essential to further improve both the performance and recyclability of LM‐polymer composite inks. While recent developments have demonstrated significant promise, several key challenges still hinder broader application. Based on our experience, three primary issues remain unresolved. First, LM droplets dispersed within a polymer matrix are electrically insulated from each other and require an additional mechanical or thermal sintering process to achieve connectivity and bulk‐level electrical conductivity. Second, the resolution of printed LM‐based circuits is still inadequate for many advanced electronic applications. Third, similar to conventional polymer composites, these materials can suffer from a decline in electrical conductivity under mechanical strain. An alternative solution is the fabrication of surface‐functionalized LM particles and biphasic LM composites, which will be discussed in the following section.

In addition to these performance‐related challenges, recycling itself poses distinct hurdles that must be addressed to enable a circular life cycle for LM–polymer composites. First, efficient separation of LM from the polymer matrix remains difficult, as strong interfacial interactions or encapsulation can limit recovery yield. Second, repeated recycling cycles may alter LM droplet size distribution or surface chemistry, leading to degraded conductivity and processability. Third, scalable and environmentally benign recycling methods are still lacking, as most current approaches rely on mechanical fragmentation or solvent treatment, which may not be practical for large‐scale applications. A succinct understanding of these bottlenecks is essential for developing next‐generation recycling strategies.

#### 4.3.4 Achieving Conductivity in Liquid Metal–Polymer Composites: Toward Sustainable Sintering

Achieving stable conductivity in LM–polymer systems requires rupturing the insulating oxide shells of LM droplets so they can coalesce into continuous networks. A variety of sintering methods have been reported—including mechanical deformation (Figure 6B), thermal or laser activation (Figure 6C), and evaporation‐assisted assembly—and their technical mechanisms are comprehensively reviewed elsewhere.^[^ ^147^, ^207^ ^]^ Here, the focus is instead on their broader implications for sustainability and circular design. Conventional approaches activate LM networks but often compromise long‐term performance: mechanical pressing produces strain‐dependent resistance, while thermal and laser treatments risk polymer damage, reducing flexibility, recyclability, and device lifetime. For example, when SIS–LM composites were subjected to UV‐laser sintering, conductive patterns were obtained but rapidly failed by short‐circuiting after only a few sensing cycles;^[^ ^11^ ^]^ similarly, mechanically sintered SIS–LM films displayed unstable resistance under strains from 0% to 100%,^[^ ^62^ ^]^ underscoring how such methods limit durability and sustainable reuse.

Recent advances instead emphasize mild, energy‐efficient, and substrate‐compatible strategies that align with the 4R framework. Evaporation‐induced sintering leverages solvent evaporation and capillary forces to assemble conductive networks under ambient conditions, avoiding high energy input and matrix degradation (Figure 6D). The incorporation of bio‐derived nanofibrils, such as cellulose or silk, further promotes droplet fusion while introducing renewable and biodegradable components that facilitate end‐of‐life disassembly.^[^ ^55^, ^122^ ^]^ In parallel, sinter‐free approaches—using reprocessable elastomer matrices (e.g., SIS) or surface‐functionalized LM particles (e.g., Ag@LM hybrids) (Figure 6E)—achieve conductivity without post‐deposition activation, directly supporting recyclability and reuse (Figure 6F).^[^ ^83^ ^]^ Viewed through this lens, sintering becomes not just a conductivity‐enabling step but a sustainability‐driven design tool: recyclable through solvent‐assisted disassembly, repairable via rewelding or photothermal reconnection, renewable with biodegradable additives, and resilient under repeated strain. By extending device lifetimes, enabling efficient material recovery, and reducing e‐waste, low‐energy and 4R‐aligned sintering strategies reposition LM–polymer composites as recoverable and reconfigurable building blocks for next‐generation soft electronics.

### 4.4 Surface‐Modified LM Particles as a Composite Ink for Flexible/Stretchable Circuits

**Figure 7:**
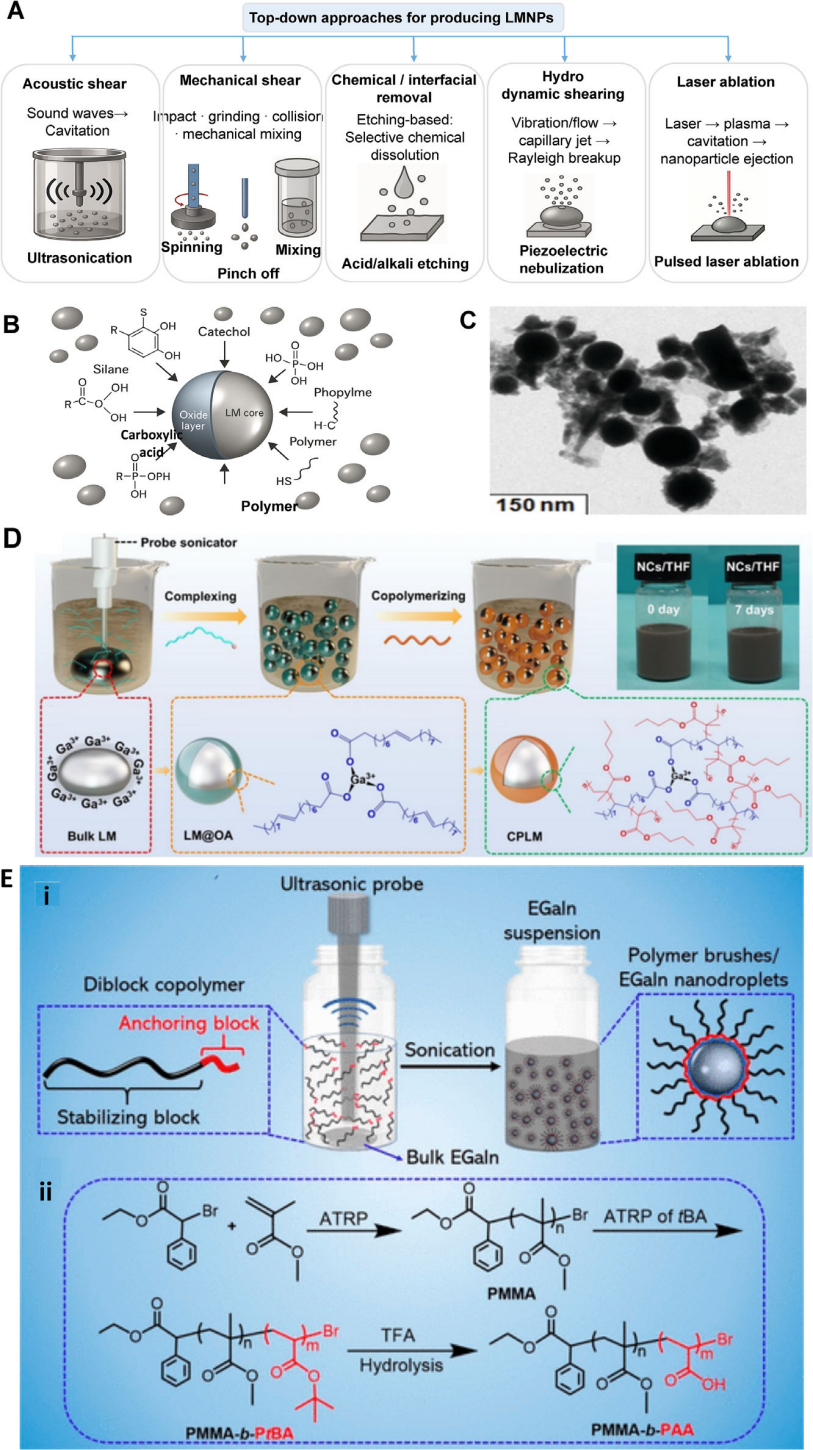
A) Methods for breaking up bulk LM into colloidal droplets. B) Schematic representation of LM surface functionalization via grafting with molecules bearing different anchoring groups. C) TEM images showing the thiolate self‐assembly at the EGaIn interface, which serves as a protective layer against oxidation. Reproduced with permission,^[^ ^226^ ^]^ Copyright 2011, ACS. D) Synthesis and fabrication route of CPLM nanocomposites (NCs). i) Schematic illustration of the synthesis process. Surface representations of ii) bulk LM, iii) LM@OA nanodroplets, and iv) core–polymer LM (CPLM) NCs. v) Dispersion stability of the NCs in THF solvent after one week. Reproduced with permission,^[^ ^229^ ^]^ Copyright 2023, Elsevier. E) Schematic illustration of i) the design of an attachable diblock copolymer and the fabrication of polymer brush‐modified EGaIn nanodroplets via ultrasonication, and ii) the synthetic pathway of the diblock copolymer using atom transfer radical polymerization (ATRP). Reproduced with permission,^[^ ^125^ ^]^ Copyright 2020, ACS Publications.

The advancement of LM‐polymer inks for flexible circuits has been significantly driven by surface modification strategies designed to enhance the stability, dispersibility, and electrical performance of these materials. One of the primary challenges associated with eutectic gallium–indium (EGaIn) alloys is their inherently high surface tension, which impedes the formation of stable and uniform dispersions required for printable inks. Furthermore, as previously discussed, LM–polymer composite inks typically require post‐processing, such as thermal or mechanical sintering, to achieve electrical conductivity. To address these limitations, surface modification of LM particles has been widely adopted with cost‐effective and scalable top‐down synthesis methods such as sonication, shearing, microfluidics, high‐speed mixing, and electrodispersion (**Figure** **7**A). Sonication techniques such as probe sonicators,^[^ ^213^, ^214^ ^]^ and ultrasonic baths^[^ ^168^, ^215^ ^]^ have been widely used to break bulk liquid metal into submicron particles.^[^ ^216^ ^]^ It should be noted, however, that if inadequate power is used, then sonication may result in heterogeneous and non‐uniform sizes of LMs due to uncontrolled standing waves.

High‐speed shear mixing and microfluidic jetting‐based methods have also been used to control the size and polydispersity of LM droplets within an emulsion. Broadly, these techniques have been used to generate LM particles ranging from 10 nm to 100 µm.^[^ ^217^, ^218^, ^219^ ^]^ Additional mechanical methods, such as spinning LM through an orifice or needle, enable further breakdown of bulk LM. Electrocapillary‐driven electrodispersion allows tuning of particle sizes between 50 and 500 µm, with Coulombic forces—regulated by the applied voltage—playing a key role in particle formation and dispersion.^[^ ^220^, ^221^, ^222^ ^]^ These approaches not only enable the breakdown of bulk LM into micro‐ and nanoscale droplets but also facilitate the formation of a native oxide layer that can be further functionalized. These surface modifications improve the chemical compatibility of LM particles with various polymer matrices, ultimately enhancing dispersion stability and enabling more effective integration into flexible electronic systems.

An important factor in the synthesis of micro‐ and nanoscale LM droplets is the formation of a self‐passivating surface oxide. When exposed to air, EGaIn LM droplets spontaneously develop a thin native oxide layer—typically only a few nanometers thick. Under ambient conditions, this Ga_2_O_3_ surface undergoes partial hydroxylation, generating ─OH groups that serve as effective anchoring sites for subsequent chemical functionalization.^[^ ^179^ ^]^ The presence of this oxide skin is particularly advantageous for high‐surface‐area LM nanoparticles (LMNPs), as it enables the attachment of a wide variety of functional groups that enhance both the stability and chemical reactivity of the particles. A range of chemical species—including silanes,^[^ ^223^ ^]^ carboxylic acids,^[^ ^147^ ^]^ catechols,^[^ ^224^ ^]^ phosphates,^[^ ^225^ ^]^ and thiols^[^ ^226^ ^]^—have been successfully grafted onto the LM surface through interactions with this oxide layer (Figure 7B). In addition to small molecules, polymers such as polyvinyl alcohol can be physically adsorbed onto the surface via non‐covalent interactions,^[^ ^227^ ^]^ while covalent grafting techniques have also been explored to further improve particle stability and tailor their surface properties.^[^ ^223^ ^]^ These surface modifications not only prevent aggregation (Figure 7C),^[^ ^226^ ^]^ and oxidation but also promote compatibility with diverse material systems, facilitating the integration of LMNPs into flexible substrates and other electronic device architectures. For example, thiol ligands have been widely used to stabilize LM nanoparticles, preventing oxidation and improving their dispersion in solvents.^[^ ^226^, ^228^ ^]^ This is particularly important for ensuring the conductivity and mechanical flexibility of the inks, which are crucial properties for printed electronics and flexible circuit fabrication. Additionally, by fine‐tuning the properties of the surface‐modified LM particles, it is possible to create inks that are compatible with a variety of substrates and manufacturing techniques.

In another approach, highly stable composite inks were fabricated by synthesizing LM nanocapsules (NCs) with a core‐shell structure through a two‐step method involving chelation and in situ free‐radical polymerization (Figure 7D).^[^ ^229^ ^]^ First, EGaIn droplets were sonicated in the air to form a thin Ga_2_O_3_ oxide layer, followed by coordination with oleic acid (OA) via strong metal–carboxylate bonds to produce LM@OA nanodroplets. These functionalized droplets served as reactive sites for subsequent polymer growth. In the second step, butyl methacrylate (BMA) was polymerized in situ, resulting in a copolymer shell surrounding the LM core. The resulting OA–BMA shell prevented aggregation, improved dispersion in both polar and nonpolar solvents, and ensured excellent colloidal stability—particularly in tetrahydrofuran (THF), where the dispersion remained stable for at least seven days. This core‐shell architecture also enhanced compatibility with various polymer matrices and served as a barrier against oxygen and moisture, preserving the LM core. Notably, the nanocapsules maintained their chemical stability and uniform dispersion even when redispersed in water, highlighting their suitability for integration into flexible, deformable polymer systems such as those used in triboelectric applications.

Another effective strategy for stabilizing EGaIn nanodroplets involves the use of diblock copolymers such as PMMA‐b‐PAA (Figure 7E).^[^ ^125^ ^]^ In this approach, the short PAA segments serve as anchoring blocks that bind to the oxide layer on the droplet surface, forming a stable interface that prevents coalescence. The longer PMMA chains act as steric stabilizers, further inhibiting aggregation and enhancing colloidal stability. This method enables the production of LM nanodroplets with high yield and narrow size distribution. The surface‐grafted diblock copolymers provide a protective coating that preserves droplet integrity, expanding their potential for diverse applications. These findings highlight the versatility of LM‐based systems in mediating surface functionalization and enabling controlled chemical interactions at the LM interface, offering new opportunities in material synthesis and functional design.

In addition to traditional chemical functionalization strategies, studies have explored the use of natural compounds like lignin for surface modification.^[^ ^230^ ^]^ Lignin has favorable chemical properties and strong surface‐binding capabilities, making it an ideal candidate for encapsulating LM particles.^[^ ^231^ ^]^ Techniques such as gallic acid self‐assembly have shown success in stabilizing LM particles, preventing oxidation, and improving the biocompatibility of the material.^[^ ^232^ ^]^ These natural encapsulants offer a sustainable alternative to synthetic surface modifiers and further enhance the performance of LM‐based inks in both electronic and biomedical applications.^[^ ^233^ ^]^

Surface‐modified LM composites are also finding applications beyond electronics. For example, in the field of drug delivery systems (DDSs), a transformable LM carrier for nanomedicine was developed, consisting of a core‐shell nanosphere with a liquid‐phase eutectic gallium‐indium core and a thiolate polymeric shell,^[^ ^234^ ^]^ and polyvinylpyrrolidone (PVP).^[^ ^235^ ^]^ This LM carrier, fabricated via sonication, demonstrated fusible and degradable properties under mildly acidic conditions, enabling the release of doxorubicin (Dox) in acidic endosomes after cellular uptake. When functionalized with hyaluronic acid, a tumor‐targeting ligand, the formulation enhanced chemotherapeutic efficacy in xenograft tumor‐bearing mice, demonstrating the versatility of surface‐modified LM particles in biomedical applications.

Hybrid LM nanoparticles and nano‐inks have also emerged as innovative platforms in magnetic hyperthermia (MH) and drug delivery, offering multifunctional solutions for cancer therapy. Since the introduction of magnetic fluids in 1965,^[^ ^236^ ^]^ MH has advanced significantly, achieving clinical approval in Europe for glioblastoma treatment in 2011, with applications extending to prostate and brain cancers.^[^ ^237^, ^238^ ^]^ Traditional MH relies on superparamagnetic nanoparticles that generate heat under alternating magnetic fields (AMF) through mechanisms such as Néel and Brownian relaxation, as well as hysteresis losses.^[^ ^239^, ^240^, ^241^, ^242^, ^243^ ^]^ However, challenges related to heating efficiency and control in biological environments persist. LMs, particularly EGaIn, present a transformative alternative due to their excellent thermal and electrical conductivity, fluidity, and biocompatibility.^[^ ^244^ ^]^ Unlike conventional magnetic nanoparticles, LMs exploit the eddy‐thermal effect for efficient heat generation under AMF, enabling rapid and localized hyperthermia.^[^ ^245^, ^246^, ^247^ ^]^ PEGylated LM platforms have been developed to facilitate drug delivery, combining doxorubicin‐loaded mesoporous silica (DOX‐MS) for dual‐responsive magnetic thermochemotherapy and CT imaging.^[^ ^248^ ^]^

Studies have demonstrated that even nonmagnetic GaIn LMs can achieve effective tumor ablation via eddy currents induced by low‐intensity AMFs, with confirmed biocompatibility and minimal toxicity.^[^ ^247^ ^]^ Further advancements include LM‐based microdevices enabling remote‐controlled drug release and enhanced tumor destruction both in vitro and in vivo.^[^ ^246^ ^]^ Despite these advantages, precise thermal regulation remains a challenge due to size‐dependent heating efficiency. However, LMs’ ability to trigger localized hyperthermia while inducing systemic antitumor immune responses has shown great promise, particularly when combined with immune checkpoint blockade (ICB) therapies.^[^ ^137^, ^249^, ^250^, ^251^, ^252^, ^253^ ^]^ For example, LM microspheres synthesized via microfluidic technology have been employed in synergistic MH and immunotherapy, as well as transarterial embolization (TAE)‐MHT treatments, demonstrating effective tumor ablation and immune activation in hepatocellular carcinoma models.^[^ ^247^ ^]^ The integration of magnetic nanoparticles (MNPs) into LMs has further expanded their therapeutic potential. Magnetic liquid metal (MLM) composites, such as Fe_3_O_4_‐loaded EGaIn, exhibit significantly enhanced heating rates under AMF, enabling faster and more effective tumor ablation compared to LMs alone.^[^ ^254^ ^]^ These MLM platforms, shaped into injectable electrodes or flexible devices with tunable stiffness, address mechanical mismatches in biological tissues while providing sustained hyperthermia, drug delivery, and even applications in neural stimulation and long‐term monitoring.^[^ ^254^ ^]^ Together, hybrid LMNPs and nano‐inks represent a versatile and powerful approach for next‐generation cancer therapies, combining thermal ablation, controlled drug release, imaging, and immunomodulation.

Furthermore, LM‐based polymer composites have emerged as advanced materials for electromagnetic interference (EMI) shielding, offering a unique balance of electrical conductivity, flexibility, and thermal management. The fluidic nature of LMs, combined with their ability to conform to rough surfaces and fill air gaps due to their surface oxide layer, significantly reduces contact thermal resistance in thermal management applications. Furthermore, interactions between Ga^3^⁺ ions and oxygen groups within the polymer matrix enhance LM droplet stability during mechanical deformation, offering superior integration compared to rigid solid fillers.^[^ ^62^, ^190^, ^255^ ^]^ A notable example is the fabrication of super‐elastic 3D conductive LM monoliths via thermal expansion, achieving an exceptional EMI shielding effectiveness (SE) of 98.7 dB and a thermal conductivity of ≈0.12 W·m^−1^·K^−1^.^[^ ^256^ ^]^ While EGaIn/PDMS composites have generally demonstrated high thermal conductivity,^[^ ^167^ ^]^ some compositions face limitations such as poor stretchability (≈100% strain), filler misalignment during deformation, and mechanical vulnerabilities like tearing and puncturing, which compromise their electrical and thermal performance.^[^ ^257^ ^]^

Achieving continuous LM networks within polymers—essential for dual‐function EMI shielding and thermal management—remains challenging. To overcome this, Hajalilou et al. used controlled oxide formation and ultrasonic‐assisted galvanic replacement to create mesoporous LM–Ag biphasic structures in SIS polymers. The resulting printable composites combined excellent electromechanical properties with EMI shielding effectiveness above 75 dB in the X‐band under 200% strain, while also functioning as thermal interface materials. Importantly, they can be recycled using biodegradable solvents, making them promising for sustainable stretchable electronics, wearables, and bioelectronics.^[^ ^21^ ^]^ Further studies showed that 3D LM network composites can double their EMI shielding under strain, reaching metallic‐level performance across 2.65–40 GHz. Strain enhances conductivity and surface‐to‐volume ratios, increasing reflection and absorption, with SEA rising from 29.7 dB at rest to 63.9 dB at 400% strain.^[^ ^258^ ^]^ Layered LM–magnetic composites enhance EMI shielding and mechanical performance, with tunable effectiveness for wearable applications.^[^ ^259^ ^]^ Together, these innovations position LM‐polymer composites as next‐generation materials for high‐performance, stretchable, and eco‐friendly EMI shielding solutions, addressing the limitations of traditional polymer composites while enabling multifunctionality in flexible electronics.

### 4.5 LM—Filler–Polymer Composite

To overcome the limitations of the above‐mentioned composite inks, the incorporation of conductive filler particles into the LM‐polymers composite has been explored. This corresponds to a new class of “biphasic” LM composites, here referred to as LM–X–Polymer systems, where “X” denotes conductive filler particles.^[^ ^19^, ^62^ ^]^ This approach not only enhances the electrical conductivity of the composite but also improves its mechanical robustness, offering a more reliable and scalable solution for next‐generation soft electronics. These composite inks allow for sinter‐free fabrication at room temperature, showcasing remarkable electromechanical behavior across various applications.^[^ ^62^ ^]^

**Figure 8:**
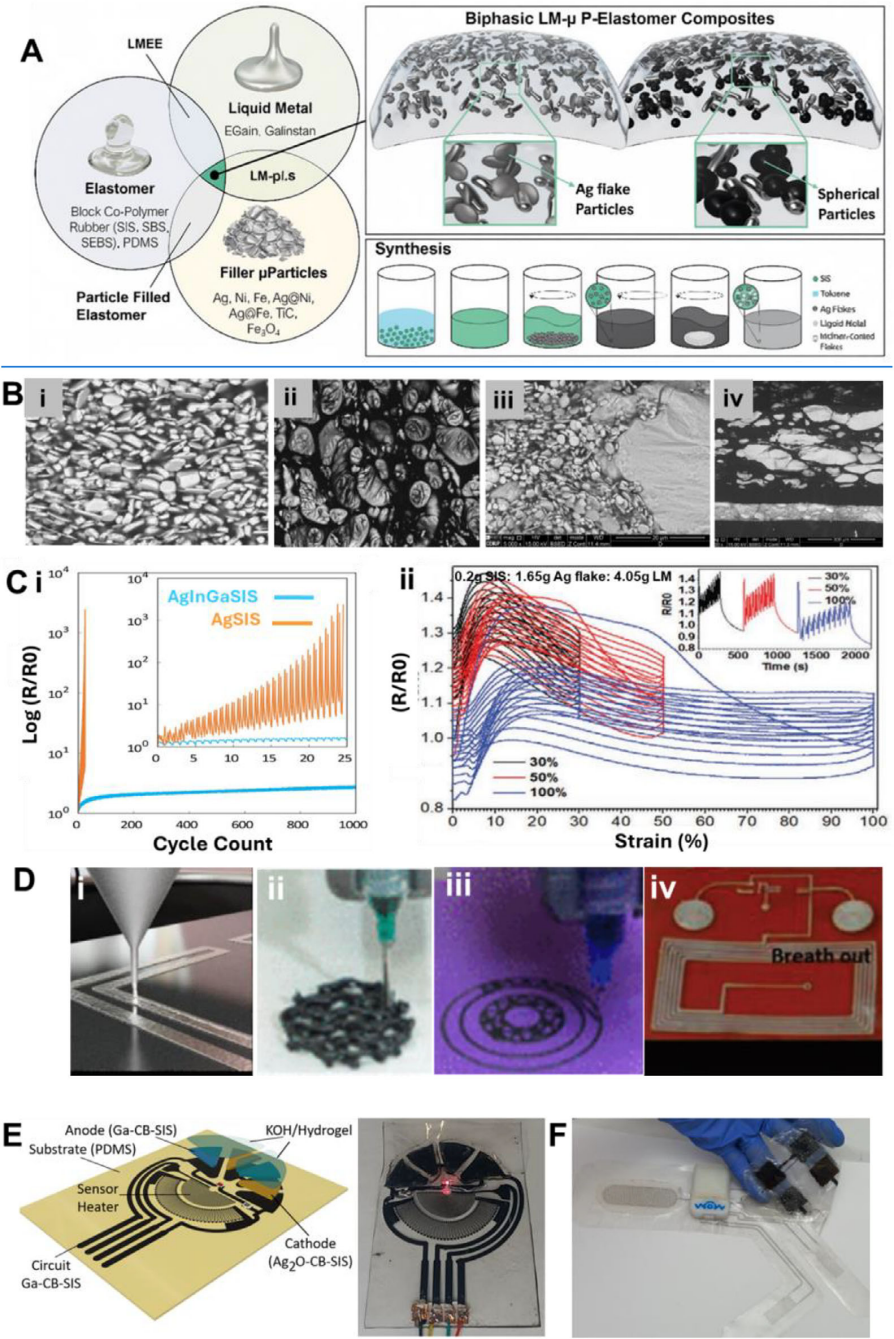
A) Schematic representation of LM‐X‐polymer composite fabrication. Reproduced with permission,^[^ ^62^ ^]^ Copyright 2022, Wiley. B) The SEM images of i) Ag flake‐SIS, ii) LM‐SIS, iii) LM‐Ag flake‐SIS, Reproduced with permission,^[^ ^62^ ^]^ Copyright 2022, Wiley, and iv) LM‐CB‐SIS composites. Reproduced with permission,^[^ ^20^ ^]^ Copyright 2024, Wiley. C) Electromechanical behvior of the Ag‐SIS compared with LM‐Ag‐SIS composite, and ii) electromechanical behaviour of LM‐Ag‐SIS at 30, 50, and 100% strain for 10 cycles. Reproduced with permission,^[^ ^62^, ^262^ ^]^ Copyright 2022, Wiley, and 2021, ACS. D) Digitally printable behavior of various biphasic LM‐Filler‐SIS polymer composites. Reproduced with permission,^[^ ^20^, ^21^, ^62^ ^]^ Copyright 2024, ACS, Copyright 2022, Wiley. E) Schematic and real image of the integrated sensor made from LM‐Ag‐SIS, and LM‐CB‐SIS composites. Reproduced with permission,^[^ ^20^ ^]^ Copyright 2024, ACS Publications. F) The composite mentioned in (E) was used to fabricate a sensor and a battery. Reproduced with permission,^[^ ^20^ ^]^ Copyright 2024, ACS Publications.

Recent efforts have shown that LM‐X‐Polymer can be designed to exhibit high conductivity, outstanding stretchability, low gauge factor, and strong adhesion to various substrates, enabling the creation of complex stretchable circuits without the need for sintering. These ternary material systems consist of binders such as block copolymer, EGaIn LM, and conductive microparticles (µP) like Ag flakes, Ag‐coated Ni, Ag‐coated Fe, Ni, Ferrite, etc. (**Figure** **8**A),^[^ ^62^ ^]^ with their corresponding SEM images in Figure 8B. They combine highly resilient and conductive (Figure 8C),^[^ ^19^ ^]^ digital printable (Figure 8D),^[^ ^20^ ^]^ repairable and self‐healing properties,^[^ ^21^ ^]^ and recyclable with sustainable approaches.^[^ ^19^ ^]^ Such characteristics make them an outstanding composite in diverse applications (Figure 8E,F). In contrast to previous LM‐polymer composites and printed EGaIn nanodroplets, these composites are inherently conductive and do not require thermal, optical, or mechanical sintering.

In one recent study, binary combinations (LM‐SIS, LM‐Ag, Ag‐SIS) are initially synthesized and characterized, followed by an evaluation of the trinary LM‐µP‐SIS composites. This includes analysis of microstructure, surface roughness, conductivity, electromechanical coupling, LM smearing/leakage under mechanical loading, and the effects of filler particle size and composition. It was found that binary combinations of Ag‐EGaIn or EGaIn‐SIS did not provide the desired properties. Only the ternary combination with conductive µP, particularly Ag, resulted in a printable, stretchable, and sinter‐free composite. A digitally printed epidermal sticker for respiration monitoring is demonstrated as a practical application.^[^ ^62^ ^]^ A Ni–EGaIn particle–carboxylated polyurethane (CPU) composite ink was printed and UV‐cured into stretchable conductors (up to 700% strain). EGaIn particles bridged Ni flakes to enhance conductivity. After blade‐cutting, the composite recovered ≈65% conductivity in 1 h and ≈97% in 24 h through dynamic hydrogen bonding, ensuring mechanical and electrical self‐healing.^[^ ^260^ ^]^

In a similar study, the LM‐Ni‐SIS composite takes advantage of a “greener” solvent composed of a mixture of methyl acetate, cyclohexane, and 4‐chlorobenzotrifluoride compared to the toluene used in block copolymers (BCPs). The composite is 3D printable, adhesive to various substrates, and can be used as a magnetic switcher.^[^ ^19^ ^]^ The LM droplets can be extracted by biocompatible materials like sugar, Epson salt, etc.^[^ ^19^ ^]^ In another study, an LM‐Ag flake‐SIS composite ink was employed as the primary current collector in LM‐based batteries due to its excellent electromechanical properties.^[^ ^20^, ^261^ ^]^ However, challenges persist in recycling LM particles due to their interaction with Ag, forming InAg_2_ intermetallic compounds.^[^ ^11^ ^]^ Depending on the application, alternative filler particles like Ni can replace Ag in LM‐Ni‐SIS composite inks, facilitating separation with external magnetic fields and employing environmentally friendly techniques for LM particle recovery.^[^ ^19^ ^]^ Moreover, the optimization of filler particle quantity is crucial, particularly when using cost‐effective materials like Ag or Au. Carbon‐based fillers present another viable option for LM‐Carbon‐Polymer composite inks, employed in anode active materials for Ga‐based LM batteries.^[^ ^20^, ^261^ ^]^ These composite inks allow for LM particle recovery using sustainable recycling methods like DES.^[^ ^20^ ^]^

The advancement of soft, stretchable, and thin‐film electronics for applications such as flexible displays, e‐textiles, robotics, sensors, and health monitoring^[^ ^22^, ^62^, ^263^, ^264^, ^265^ ^]^ is increasingly constrained by the lack of efficient, flexible energy storage solutions. Current systems, including EEG, ECG, and EMG patches, depend on rigid external power sources or bulky batteries,^[^ ^266^, ^267^, ^268^ ^]^ limiting their integration into fully flexible devices. To address this, research has focused on developing stretchable, high‐capacity, and rechargeable energy storage systems. Other review articles have examined LM‐based batteries and compared their fast charge‐transfer kinetics, resistance to electrode degradation,^[^ ^269^ ^]^ and safety with and without elevated temperatures.^[^ ^270^ ^]^

However, the rise of disposable electronic patches presents environmental concerns due to escalating e‐waste.^[^ ^11^ ^]^ This has driven interest in recyclable materials, where LM‐based polymer composites offer a promising solution. In terms of energy viewpoint, LMs, particularly gallium and its alloys like EGaIn, exhibit fluidity, high conductivity, self‐healing properties, and recyclability—making them ideal candidates for flexible and sustainable batteries.^[^ ^62^, ^271^ ^]^ Gallium's low melting point (29 °C) is close to room temperature, which facilitates ionic diffusion and minimizes volume fluctuations during cycling.^[^ ^62^, ^272^ ^]^ Its inherent self‐healing behavior further improves cycle stability and mechanical resilience under strain.^[^ ^273^ ^]^ While gallium‐based materials have long been explored in stretchable electronics, their application in energy storage has gained momentum only recently. Incorporating gallium into electrodes has been shown to enhance self‐healing and cycling stability in lithium‐ion batteries,^[^ ^274^, ^275^ ^]^ while gallium‐doped MnO_2_ has improved Zn‐ion battery performance, delivering extended cycle life and high capacity.^[^ ^276^ ^]^ Despite challenges such as low areal capacity in early designs,^[^ ^277^ ^]^ innovations like EGaIn‐MnO_2_ batteries demonstrate promising cycling stability.^[^ ^271^ ^]^

A notable breakthrough is the development of fully 3D‐printable, stretchable Ag‐Ga batteries using LM‐X‐Polymer composite inks. These systems utilize sinter‐free composites—such as EGaIn‐Ag flake‐SIS for current collectors and Ga‐CB‐SIS for anodes—processed entirely at room temperature, ensuring compatibility with flexible substrates. These LM‐polymer composites offer exceptional stretchability (up to 600%), stable conductivity, and self‐healing capabilities through both passive and active mechanisms. For instance, damage to the Ga‐CB‐SIS electrode can be autonomously repaired via Ga flow or actively restored using Toluene vapor treatment, leveraging the reversible crosslinking of the SIS polymer matrix.^[^ ^11^, ^62^ ^]^ This dual self‐healing mechanism, driven by the fluidic nature of LMs and polymer responsiveness, ensures a prolonged device lifespan even under mechanical stress. Additionally, the recyclability of LM particles using DESs further aligns these systems with sustainable electronics initiatives. In summary, LM‐X‐Polymer composite inks represent a transformative approach for next‐generation stretchable, self‐healing, and eco‐friendly energy storage devices, addressing both functional performance and environmental challenges in flexible electronics.^[^ ^62^ ^]^

## 5 Self‐Healing Behavior of LM Composites

**Figure 9:**
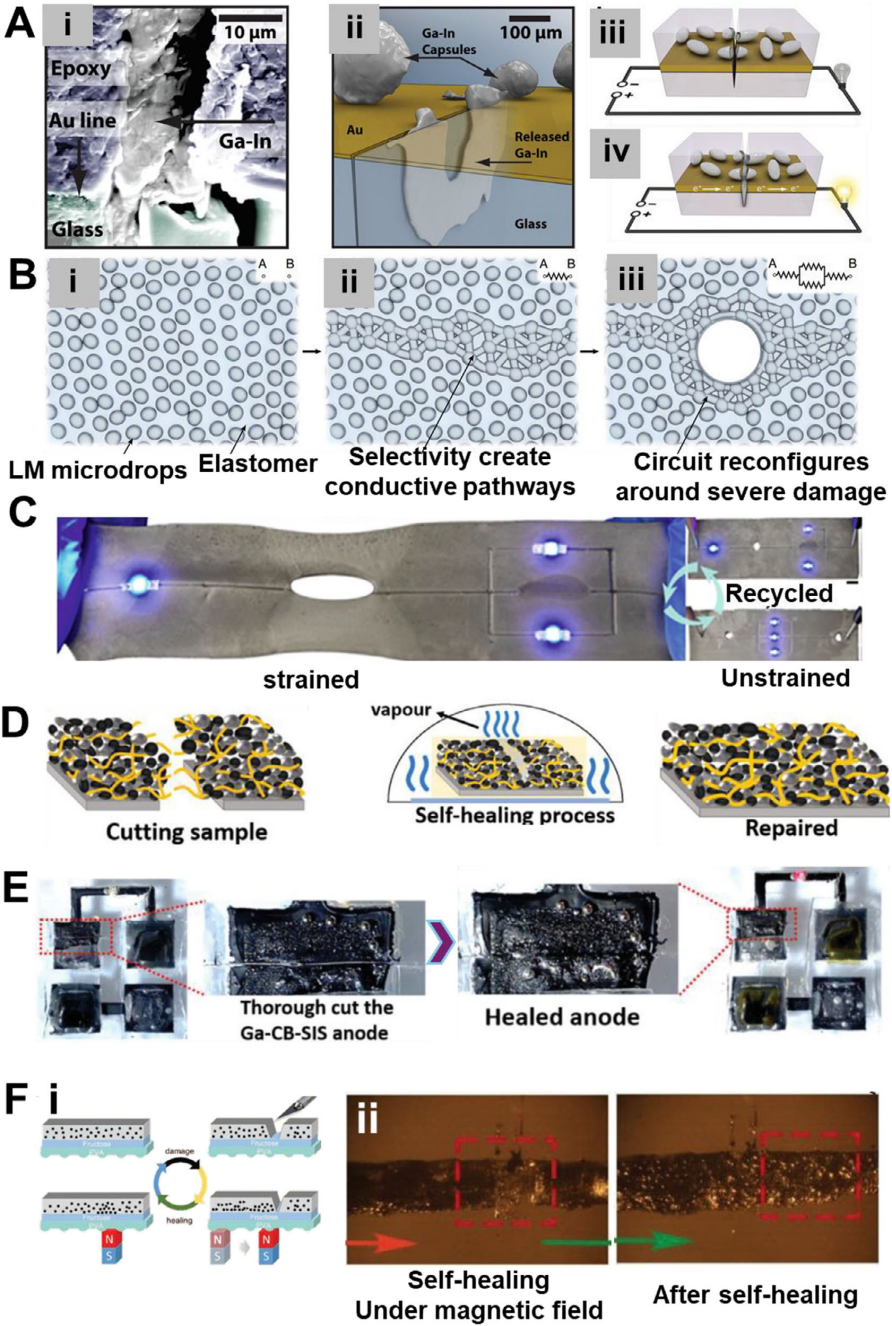
A,i) False‐colored cross‐sectional SEM image showing the damaged area and the corresponding release of liquid metal. ii) Micro‐CT image with a superimposed schematic illustrating microcapsules and released liquid metal filling the crack plane in the healed specimen. iii) Crack formation disrupts the conductive pathway and ruptures the embedded capsules. iv) Released liquid metal flows into the damaged region, reestablishing electrical conductivity. Reproduced with permission,^[^ ^278^ ^]^ Copyright 2012, Wiley. B) Schematic of the self‐healing mechanism: an initially insulating composite becomes electrically conductive through selective compression forming LM traces. Upon damage, the LM trace autonomously reconfigures to preserve conductivity. Inset: equivalent electrical circuit diagram. Reproduced with permission,^[^ ^279^ ^]^ Copyright 2018, Nature Publishing Group.Self‐healing soft matter composite. i) LM‐elastomer composite under stretch and twist, showing conductive traces. Insets: undeformed sample and optical micrograph of LM microdroplets at ϕ = 50%. ii) Schematic of the self‐healing mechanism: initially insulating composite becomes conductive under compression; after damage, LM traces autonomously reconFigure to maintain conductivity. Inset: equivalent electrical circuit. iii) Reconfigurable material (ϕ = 50%) transmitting d.c. power and digital signals to operate a counter display, which remains functional despite severe damage to all traces. Reproduced with permission,^[^ ^279^ ^]^ Copyright 2018 Nature Materials. C) The LM composite system was developed for soft circuits, featuring robust, self‐healing conductive traces with strain‐invariant resistance across different resistance levels. The material maintains LED functionality in both unstrained and recycled states, even after stretching. The composite has a composition of ϕ = 60% and δ = 20%, with a scale reference of 10 mm. Reproduced with permission,^[^ ^40^ ^]^ Copyright 2021, Nature Publishing Group. D) Vapor‐assisted healing of the SIS–LM composite, enabled by the reversible physicochemical properties of SIS. Reproduced with permission,^[^ ^20^ ^]^ Copyright 2024, Wiley. E) Practical application of this mechanism in a damaged battery, where the anode is successfully healed. Reproduced with permission,^[^ ^20^ ^]^ Copyright 2024, Wiley. F) Repairing damaged circuits via magnetic‐field‐driven liquid metal flow. Reproduced with permission,^[^ ^137^ ^]^ Copyright 2019, Wiley.

While self‐healing in composites may not directly reduce electronic waste, it plays a crucial role in prolonging device life by enabling the repair of damaged conductive paths. In LM composites, this regenerative functionality stems from LM droplets dispersed throughout a polymer host. Thanks to their fluidic properties, these droplets autonomously re‐establish electrical connectivity when structural disruption occurs. An illustrative example is shown in **Figure** **9**A, where Ga‐In‐filled microcapsules are integrated into a self‐repairing circuit. Structural and schematic analyses reveal their distribution over a gold line and the mechanism by which they facilitate healing. Upon experiencing a crack or break, the capsules rupture, allowing LM to flow into the damaged area and rapidly bridge the gap. The multilayer test configuration depicted in the schematic captures the progression of damage and subsequent restoration. Impressively, the released LM enables nearly instantaneous recovery of electrical function—exceeding 99% restoration efficiency within less than 200 microseconds. Even with a relatively low concentration of microcapsules, the system demonstrated complete circuit repair, highlighting its effectiveness.^[^ ^278^ ^]^ The LM particles within the matrix play a key role in self‐healing damaged circuits. However, in some cases, they alone cannot ensure complete healing. The binders and matrix also have fundamental roles. For example, LM droplets in rigid materials (E > 2.8 GPa) enable only limited self‐healing, as conductivity is disrupted after damage and the LM is depleted after a single repair.^[^ ^278^ ^]^ Recently, however, vitrimer‐based LM–epoxy composites have been reported to overcome several of these limitations by combining high conductivity with recyclability, reprocessability, and improved mechanical resilience.^[^ ^41^ ^]^ To overcome these challenges, incorporating Ga‐based LM alloy droplets into a silicone elastomer to fabricate a stretchable circuit is a viable solution (Figure 9B). The LM droplets ruptured and coalesced under extreme local pressure, forming locally conductive pathways with high electrical conductivity (σ = 1.37 × 10^3^ S cm^−1^ for ϕ = 50%) (Figure 9B(i,ii)). The material autonomously forms new electrical pathways after damage, allowing a four‐channel clock display to keep functioning despite cuts and tears by rerouting current through droplet–droplet connections (Figure 9B‐iii).^[^ ^279^ ^]^ In contrast, previous LMEE synthesis approaches required high volume loadings (ϕ ≥ 50%) for conductivity and lacked autonomous self‐repair.^[^ ^280^, ^281^ ^]^ In microfluidic circuits, self‐healing often temporarily interrupts conductivity and needs manual restoration. Light pressure (<100 kPa) could create conductive networks via “mechanical sintering”, but any inadvertent application of pressure could risk activating neighboring traces, causing electrical failures.^[^ ^282^ ^]^

Using thermoplastic polymers like SIS as binders in LM composites have the potential to enable both electrical and mechanical self‐healing. A reconfigurable LM–SIS composite was developed as a regenerative soft electronic platform, enabling strain‐invariant, self‐healing, and recyclable circuitry.^[^ ^20^ ^]^ Initially insulated LM droplets form conductive networks through embossing, reaching conductivities of 150 S cm^−1^ at 0% strain and 45,400 S cm^−1^ at 1200% strain. The composite withstands over 900% strain, retains conductivity after damage, and allows reprocessing for circuit reconfiguration or recycling, making it ideal for resilient soft electronics Figure 9C.^[^ ^40^ ^]^ In the anode part of the LM battery, composed of LM‐CB‐SIS, the SIS polymerundergoes a phase transition into a gel‐like state when exposed to toluene vapor, allowing LM mobility and enabling active self‐repair in cases of severe damage. This synergistic interaction between LM and SIS enhances electrical conductivity and mechanical resilience, ensuring effective restoration of the damaged electrode (Figure 9D,E).^[^ ^20^ ^]^ In another approach, magnetic particles in an LM–polymer matrix enabled self‐healing circuits. As shown in Figure 9D, Fe–EGaIn droplets guided by a NdFeB magnet bridged knife cuts under a PVA film, restoring an LED circuit. Healing completed within ≈3 s for a single cut and ≈10 s for multiple cuts, with wider lines recovering faster and current flow nearly fully restored.^[^ ^137^ ^]^

## 6 Selection of Stretchable Substrates Entangled with LM Extraction in Soft Circuits/Electronics

Stretchable substrates such as polymers, hydrogels, and elastomers are essential in soft electronics due to their mechanical flexibility, thermal stability, and compatibility with LM composite inks. Their properties directly impact circuit performance, durability, and recyclability. Crucially, the interaction between LM inks and substrates influences both conductivity and the efficiency of LM extraction, which is key for sustainable design. Surface modifications and self‐healing mechanisms can further enhance ink adhesion and circuit resilience. To advance high‐performance and eco‐friendly soft electronics, it is important to select substrates that not only support reliable LM integration but also enable effective recycling of both materials.

### 6.1 Role of Polymer Substrates in Soft Electronics

Polymer substrates play a central role in stretchable electronics by offering a tunable platform for integrating LM inks. Their interaction with LMs governs print quality, circuit integrity, and reusability. Enhancing this interface—through chemical modifications or mechanical reconfiguration—improves device reliability and supports material recovery. Therefore, optimizing polymer–LM compatibility is critical for the development of next‐generation, sustainable soft electronic systems. These substrates can be categorized as follows.

#### 6.1.1 Biodegradable and Recyclable Polymer Substrates for Soft Electronics

Biodegradable polymers are gaining prominence in the field of soft electronics, offering a sustainable and eco‐friendly alternative to traditional materials. These “green materials” are particularly valuable due to their ability to support end‐of‐life disassembly and material recovery (recyclability) while leveraging bio‐based, renewable feedstocks. As electronic devices become widespread, addressing the growing issue of electronic waste is crucial. Substrates, which make up the majority of a device's weight, significantly influence its degradation behavior and overall environmental footprint.^[^ ^283^, ^284^, ^285^ ^]^ The development of biodegradable or disintegrable polymer substrates has therefore become a key focus, with extensive research on materials that balance sustainability with required mechanical performance.^[^ ^284^, ^286^ ^]^ Among the various options, biodegradable polymers derived from natural sources, such as cellulose‐based materials, polylactic acid (PLA), polyhydroxyalkanoates (PHA), and starch‐based polymers, have shown promise (**Table** **3**). These materials naturally decompose over time, reducing pollution and resource depletion, making them ideal candidates for sustainable electronics. Notably, many of these substrates are renewable (bio‐derived), recyclable via biodegradation or green‐solvent disintegration, and can maintain device resilience during service when engineered appropriately.

**Table 3:** Biodegradable Polymer Substrates—Materials, Preparation Methods, Applications, and References.

Biodegradable polymer	Materials	Preparation method	Applications	Refs.
Polyvinyl alcohol (PVA)	PVA powder, deionized water	Solution casting	Gel‐electrolyte formation, electrode modification, functionalizing agent for biosensors, field‐effect transistors	[331, 332, 333, 334, 335]
Polyvinyl alcohol/cellulose nanocrystal (PVA/CNC)	PVA, CNC	Multilayer film formation to enhance water vapor and oxygen barrier performance	Improved water resistance, reduced permeability for flexible substrates	[334]
Polyvinyl alcohol/starch	PVA, starch, citric acid, glutaraldehyde	Crosslinking with citric acid and glutaraldehyde to reduce hydrophilicity	Enhanced water resistance, mechanical reinforcement	[336]
Chitosan (QT)	Chitosan, acetic acid	Solution casting	Solid support for electrochemical sensing, flexible bioelectronic devices	[337]
Chitosan‐based films	Chitosan, CaCl_2_, NH_4_SCN, potato starch, MXene, carbon, TiO_2_, Cu‐doped carbon dot	Solution casting	Flexible circuits, pressure sensors, colorimetric pH sensors, energy storage, triboelectric nanogenerators	[338, 339, 340, 341, 342, 343]
Starch	Amylose, amylopectin	Solution casting	Binder, diluent, disintegrating agent, polymeric film development	[344, 345, 346]

**Figure 10:**
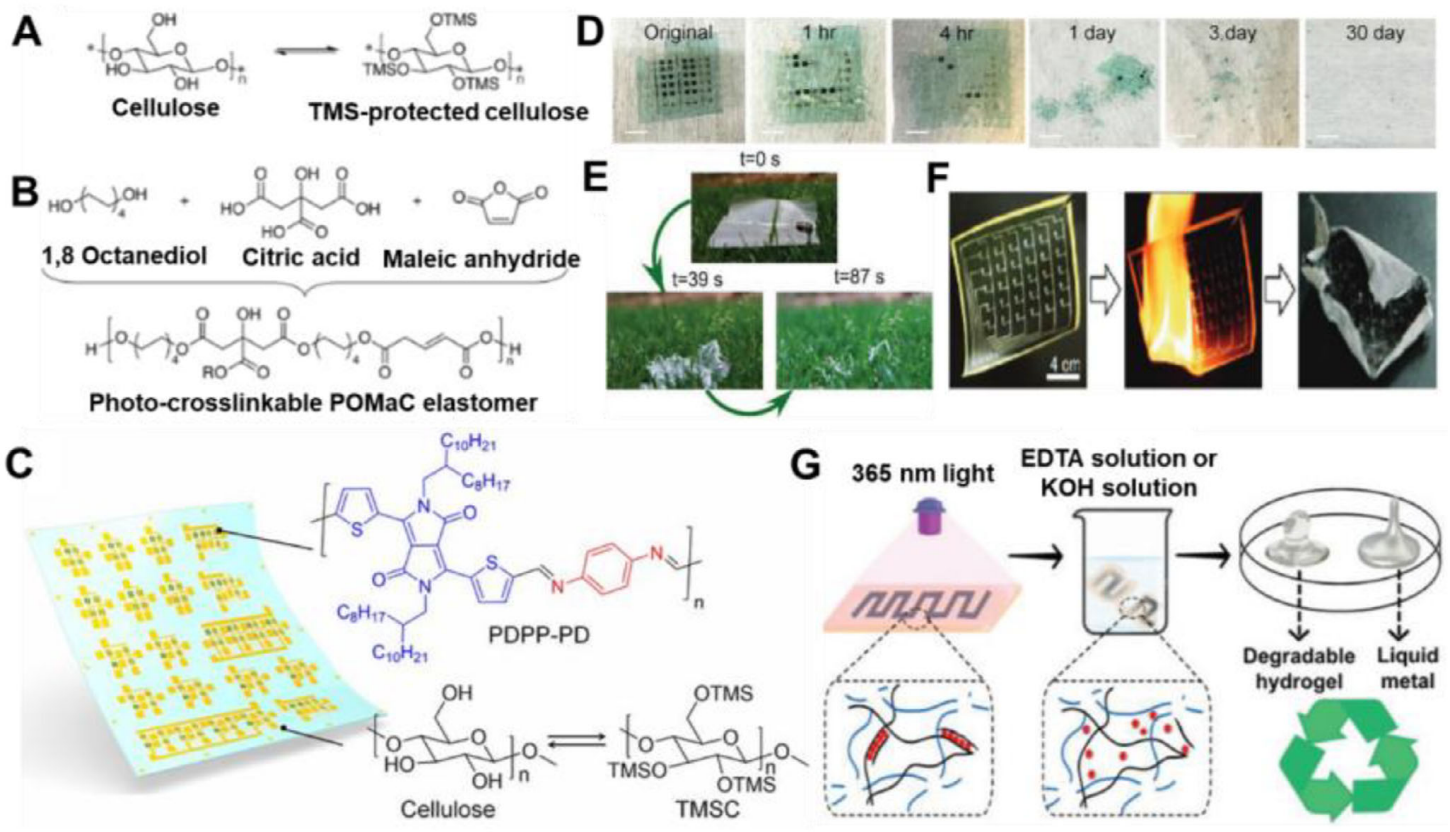
A,B) Chemical structures of widely used biodegradable substrates. A) Cellulose is a naturally sourced, flexible, and thermally stable material. Ultrathin films (≈800 nm) can be fabricated through hydrolyzable trimethylsilyl group modification. Reproduced with permission,^[^ ^287^ ^]^ Copyright 2020, MDPI. B) POMaC is a synthetic elastomer that is both stretchable and biodegradable, serving effectively as a substrate. Reproduced with permission,^[^ ^283^ ^]^ Copyright 2018, ACS Publications. C) Flexible device incorporating a degradable polymer as the active material or substrate. Reproduced with permission,^[^ ^303^ ^]^ Copyright 2017, PNAS. D) Photographic images illustrating the progressive disintegration of the devices over time. Reproduced with permission,^[^ ^303^ ^]^ Copyright 2017, PNAS. E) The electronic device, consisting of an LED connected to Fe‐doped EGaIn droplets printed over a PVA/fructose film, which degrades in nature when exposed to the rain for 87 s. Reproduced with permission,^[^ ^137^ ^]^ Copyright 2019, Wiley. F) The Ni–EGaIn conductive traces deposited on paper substrates are compatible with thermal degradation, offering a biodegradable and eco‐friendly disposal route. Reproduced with permission,^[^ ^307^ ^]^ Copyright 2019, Sci. China Mater. G) Schematic of the hybrid double‐network (DN) degradable hydrogel used as a substrate. The hydrogel can be degraded under 365 nm UV light, enabling recycling, while the LM droplets are recovered using a KOH and sugar solution. Reproduced with permission,^[^ ^19^ ^]^ Copyright 2023, Wiley.

Cellulose, as an example, is a naturally derived polymer with flexibility, thermal stability, and biocompatibility suited to flexible devices. Its chemical structure, as shown in **Figure** **10**A, can be modified by introducing hydrolyzable trimethylsilyl groups, enhancing its degradability and enabling the production of ultrathin films (≈800 nm) that retain the material's desired properties.^[^ ^283^, ^287^ ^]^ In the 4R context, cellulose substrates combine renewability (bio‐origin) with resilience (mechanical/thermal stability) during operation and recyclability at end‐of‐life; solvent or enzymatic pathways can also facilitate LM reclamation from cellulose‐supported circuits. In addition to cellulose, other plant‐based alternatives, such as bamboo fibers, hemp fibers, and soy‐based polymers, also offer biodegradable and compostable options to replace petroleum plastics.^[^ ^288^, ^289^ ^]^ In one study, cellulose‐based paper was used as a low‐cost, flexible substrate to create stretchable LM conductors with a kirigami structure, achieving over 800% stretchability while maintaining stable conductivity under large deformations.^[^ ^290^ ^]^ The substrate could be dissolved in water, enabling recovery of the liquid metal.

On the other hand, synthetic polymers provide unique advantages such as tunable mechanical properties and controlled degradation rates, allowing for the precise customization of material performance for specific applications.^[^ ^291^, ^292^ ^]^ Polyester‐based elastomers, such as poly(diol citrates) and poly(glycerol sebacate) (PGS), have also shown promise due to their stretchability and biocompatibility.^[^ ^293^, ^294^ ^]^ For example, stretchable Si‐based sensors have been fabricated using poly(1,8‐octanediol‐co‐citrate) (POC), which dissolves completely in PBS after 12 h.^[^ ^295^ ^]^ These elastomers can also be photo‐cross‐linked by introducing maleic anhydride to form poly(octamethylene maleate citrate) (POMaC),^[^ ^292^ ^]^ expanding the range of materials for stretchable, degradable substrates.^[^ ^296^, ^297^ ^]^ poly(octamethylene maleate citrate) (POMaC), a synthetic biodegradable elastomer, offers stretchability while maintaining thermal stability, making it suitable for flexible electronics (Figure 10B).^[^ ^283^, ^292^ ^]^ EGaIn LM has been successfully patterned on a biodegradable poly(octamethylene maleate citrate) (POMaC) substrate, highlighting the compatibility of LM with transient polymer platforms. Using a low‐harshness fabrication route—combining stencil lithography with centrifugal force–assisted deposition—EGaIn was deposited without compromising the structural integrity of the polymer. The use of POMaC as a substrate not only provided the necessary flexibility and stretchability for soft electronic applications but also introduced biodegradability, enabling the development of transient devices designed to degrade or dissolve after use. This integration underscores the potential of biodegradable synthetic polymers as functional substrates for liquid‐metal‐based flexible and eco‐friendly electronics.^[^ ^298^ ^]^

Other synthetic materials, such as poly(1,8‐octanediol‐co‐citrate) (POC), PEG sebacate diacrylate (PEGSDA), poly(propylene fumarate) (PPF), poly(glycerol‐co‐sebacate) acrylate (PGSA) and poly(glycerol sebacate) (PGS), have been explored for their ability to dissolve rapidly in physiological conditions, offering additional flexibility in the design of transient devices.^[^ ^297^, ^299^, ^300^, ^301^ ^]^ Despite the potential of these materials, one of the challenges in utilizing biodegradable polymers as substrates is their relatively low thermal stability, which limits their use in high‐temperature fabrication processes required for commercial flexible electronics.^[^ ^286^ ^]^ Therefore, there is a pressing need to develop polymer substrates that not only biodegrade but also exhibit thermal robustness, recyclability, and the ability to disintegrate in environmentally friendly solvents. This is a crucial step towards reducing the environmental impact of electronics and promoting sustainability in the industry. One strategy to achieve a substrate with high thermal stability is the direct fabrication of devices on cellulose nanofiber (CNF) substrates, which offer flexibility, transparency, and thermal stability.^[^ ^302^, ^303^ ^]^ CNF‐based papers, derived from wood, are well‐suited for the roll‐to‐roll fabrication of flexible circuits, including conductive lines/traces.^[^ ^304^ ^]^ These substrates degrade slowly over several months in the presence of fungi, reducing the environmental impact of consumer electronics.

Several studies have demonstrated LM usage with cellulose nanofiber (CNF) substrates. For example, flexible LM/CNF composite films were fabricated where CNF provided structural stability and flexibility, enabling highly efficient and broadband EMI shielding.^[^ ^305^ ^]^ In another study, a symmetric gradient structure was introduced in CNF/FeCo/LM composite films, in which CNF functioned as both a mechanical scaffold and an electromagnetic wave absorption layer, resulting in robust performance with ≈39 dB EMI shielding, electrical insulation, and mechanical strength.^[^ ^306^ ^]^ However, the micrometer‐scale thickness of CNF papers limits their use in ultrathin devices. To overcome this, trimethylsilyl‐functionalized cellulose (Figure 10C) has been developed, enabling ≈800 nm substrates with robust thermal and solvent stability that are suitable for direct device fabrication. Circuits on these substrates degrade within a month (Figure 10D)^[^ ^303^ ^]^ and within 15 days in soil.^[^ ^212^ ^]^ Building on such advances, the transfer printing approach further enhances flexibility by enabling devices to be integrated onto a wide range of biodegradable materials—including PVA, PVA–fructose composites, silk, paper, and cellulose—each exhibiting distinct degradation rates (Figure 10E–F).^[^ ^112^, ^303^, ^304^, ^307^, ^308^ ^]^ A study demonstrated that EGaIn nanoparticles deposited on electrospun PCL films formed conductive, biocompatible, and partially biodegradable composites. The films maintained stable resistance under bending and twisting and were applied as proof‐of‐concept sensors for weight, sound, and breath monitoring. These properties highlight their strong potential for wearable and implantable bioelectronics.^[^ ^309^ ^]^ In another study, a simple approach for patterning EGaIn was reported by spray‐coating copper nanoparticles (Cu NPs) onto polymethyl methacrylate (PMMA) substrates using PMMA‐dissolving solvents to form rough, embedded metallic layers.^[^ ^310^ ^]^ Following vapor treatment to remove the native oxide on EGaIn, imbibition‐driven reactive wetting was triggered, enabling the liquid metal to selectively spread along the CuNP patterns. This method produced conductive features with dimensions down to ≈1 mm lines and ≈500 µm tips, providing a low‐cost, lithography‐free strategy for flexible and stretchable electronics. In another study, an LED device using Fe‐doped EGaIn droplets printed on a PVA/fructose film rapidly degrades in water. The film dissolves in 205 seconds, turning off the LED, aided by the oxide layer's weak passivation.^[^ ^137^ ^]^ In natural conditions, dissolution occurs even faster—within 85 seconds—highlighting the device's eco‐friendly degradability. The LED and EGaIn droplets can be recovered and reused.^[^ ^137^ ^]^ Moreover, the demand for wearable and implantable electronics requires materials that are not only biodegradable and recyclable but also stretchable to conform to dynamic surfaces such as the skin, heart, or brain.

For biocompatible applications, substrates must be selected based on their compatibility with living organisms and ecosystems to ensure suitability for biomedical use while minimizing environmental impact. Materials such as poly(lactic‐co‐glycolic acid) (PLGA), silk, and chitosan have been widely utilized for this purpose.^[^ ^311^, ^312^, ^313^ ^]^ In another approach, a hybrid double‐network (DN) degradable hydrogel, as a substrate, was developed for bioelectronics, combining mechanical strength, non‐drying behavior, and photodegradability.^[^ ^19^ ^]^ The substrate consisted of ionically cross‐linked alginate (via Ca^2^⁺) and polyacrylamide (PAAm) covalently cross‐linked using a photodegradable o‐nitrobenzyl (ONB)‐based crosslinker. The incorporation of poly(ethylene glycol) (PEG) into the crosslinker enhanced water solubility, mechanical properties,^[^ ^22^ ^]^ and degradation control.^[^ ^19^ ^]^ The resulting DN hydrogel enabled direct digital printing of an LM–Ag flake–SIS composite ink.^[^ ^62^ ^]^ As a proof‐of‐concept, wearable motion sensors were fabricated, demonstrating reliable performance. The hydrogel substrate was fully degradable using UV light and chelating solutions (EDTA or KOH), allowing for efficient recovery of embedded metals and supporting the development of recyclable soft electronics (Figure 10G).^[^ ^19^ ^]^ Additionally, novel bio‐based copolymers, such as the combination of α‐thioctic acid (TA) and butyl acrylate (BA), have been introduced as substrates for flexible electronics. These materials exhibit full recyclability, rapid self‐healing (within 5 min at 60 °C), and enhanced adhesion to LMs, making them ideal for biomedical and wearable applications. The strong hydrogen and disulfide bonds in these materials contribute to their robustness and ability to interact effectively with liquid metal and substrates, further enhancing their potential for soft electronics.^[^ ^314^ ^]^

Polyimide (PI) is another example of a thermally stable polymer, derived from diamine and dianhydride monomers, and is widely used in aerospace, microelectronics, and optoelectronics. Its excellent thermal and mechanical properties make it an essential material in various electronic applications, including antennas, transistors, capacitors, batteries, RFID systems, solar cells, and flexible OLED displays.^[^ ^315^, ^316^ ^]^ In one study, EGaIn nanoparticle inks were spray‐coated onto flexible PI substrates and subsequently activated by intense pulsed light (IPL) photonic sintering, which fractured the oxide shells and enabled coalescence into conductive networks with conductivities up to 6650 S cm^−1^.^[^ ^317^ ^]^ In another study, serpentine‐shaped EGaIn/Au/Ti interconnects were patterned on polyimide‐coated glass substrates and encapsulated with PDMS, yielding flexible and stretchable liquid‐metal‐based interconnects suitable for soft electronic applications.^[^ ^298^ ^]^ However, traditional PI synthesis typically involves toxic organic solvents such as N‐methyl‐2‐pyrrolidone (NMP) and dimethylacetamide (DMAc), which pose significant environmental and health risks.^[^ ^318^, ^319^, ^320^, ^321^ ^]^ A hydrothermal water‐based method enables greener PI synthesis at ≈200 °C, yielding fluorinated PIs with high solubility, and >500 °C thermal stability, while improving humidity resistance;^[^ ^322^ ^]^ despite slightly lower molecular weights, they retain good fusibility and narrow distribution (PDI < 2).^[^ ^318^ ^]^ Structural tuning—rigid imide/aromatic rings,^[^ ^323^ ^]^ and polar/bulky groups like ─OH, ─CF_3_^[^ ^324^ ^]^—further enhances stability, dissolvability, and recyclability. A BAHPFP/6FDA‐based PI substrate combines excellent multi‐functional properties with alcohol‐enabled recyclability for flexible electronics.^[^ ^325^ ^]^ Recent advances also demonstrate the potential of PI–LM hybrids, such as transparent LM/CPI films with light programmability,^[^ ^326^ ^]^ PI/LM composites for improved tribological coatings,^[^ ^327^ ^]^ and LM–PI/PTFE actuators mimicking tendril motion for soft robotics.^[^ ^328^ ^]^ Yet, fluoropolymers remain environmentally persistent, with bioaccumulative byproducts and health risks,^[^ ^329^ ^]^ and although industry views them as “low concern”,^[^ ^330^ ^]^ degradation products are problematic, necessitating degradable analogues or improved end‐of‐life strategies.

#### 6.1.2 Role of Thermoset Polymer Substrates in Soft Electronics

Thermoset polymers—such as epoxy, phenolic, polyurethane, polyester, vinyl ester, melamine‐formaldehyde, urea‐formaldehyde, silicone, and polyimines—form permanent cross‐linked networks upon curing, imparting high thermal stability, mechanical strength, and chemical resistance.^[^ ^347^, ^348^ ^]^ However, their infusible and insoluble nature hinders recycling, contributing to e‐waste. To address this, degradable and dynamic bond chemistries are being explored.^[^ ^349^, ^350^ ^]^ In soft electronics, thermosets are widely used as substrates for printing and patterning LM composites and interconnects, with their chemistry directly influencing 4R goals.

**Figure 11:**
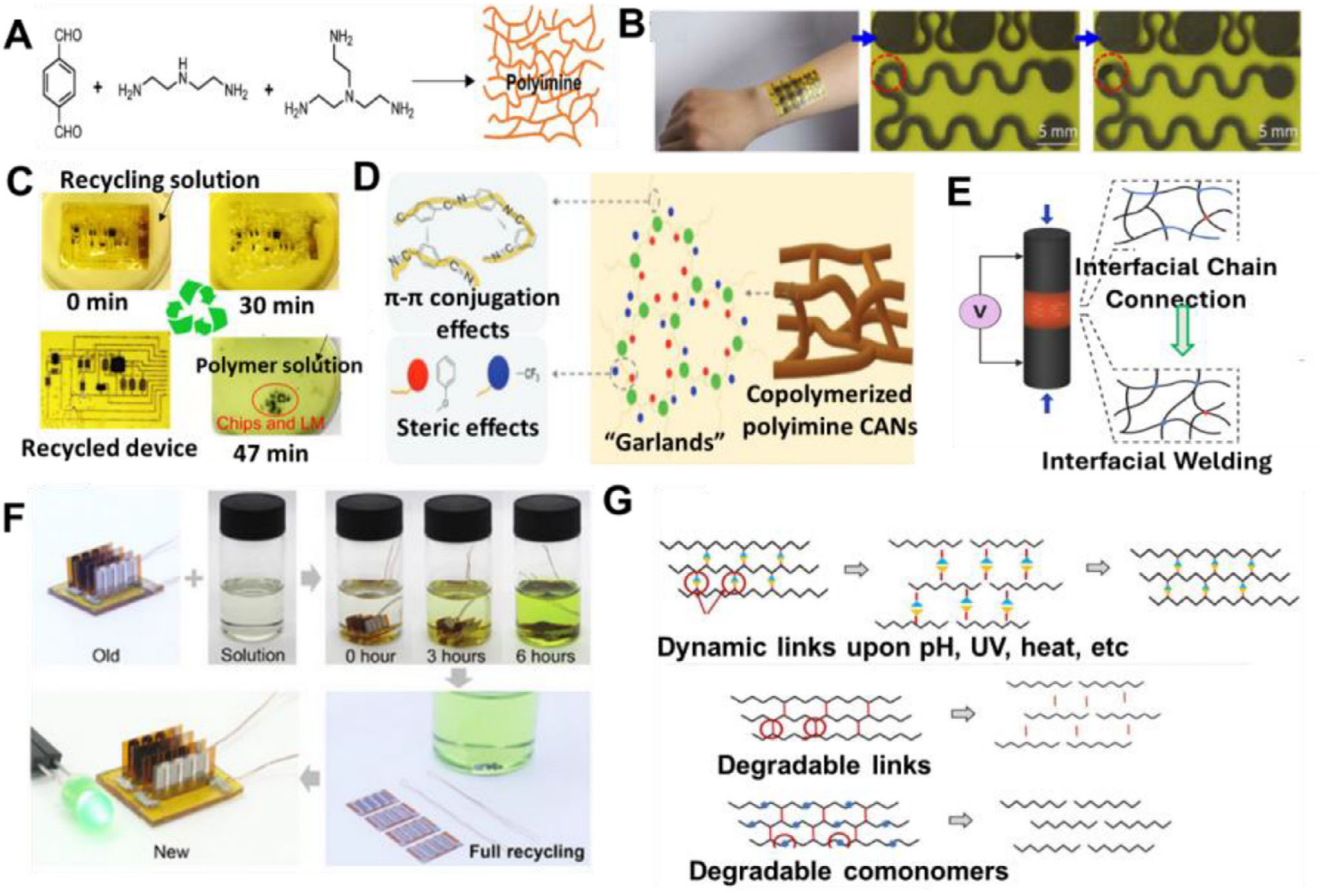
A) Preparation of polyimine films based on dynamic covalent bond exchange. Reproduced with permission. Reproduced with permission,^[^ ^351^ ^]^ Copyright 2022, Elsevier. B) The malleable e‐skin conforms to a human arm (left) and can be rehealed after mechanical damage by applying a rehealing agent and heat pressing (middle, right). Reproduced with permission. Reproduced with permission,^[^ ^355^ ^]^ Copyright 2018, Science. C) The recycling process of the multifunctional device captured in optical images. Reproduced with permission,^[^ ^205^ ^]^ Copyright 2020 Science. D) Schematic of the microstructure and architecture of CO‐PIMs films. Reproduced with permission,^[^ ^352^ ^]^ Copyright 2023, Wiley. E) Schematic of the welding process of polyimine composites via Joule heating. Reproduced with permission,^[^ ^357^ ^]^ Copyright 2021, IOP. F) The recycling process of the TEG, with optical images at different steps. The new TEG is connected in series with an LED and a 4 V DC source (bottom left). Reproduced with permission,^[^ ^358^ ^]^ Copyright 2021, Science. G) Schematic illustrating the concept of designing thermosets with inherent recyclability. Reproduced with permission,^[^ ^360^ ^]^ Copyright 2022, MDPI.

For example, polyimines have emerged as a highly promising LM‐compatible substrate platform for next‐generation sustainable electronics. These polymers are defined by dynamic imine (─C═N─) linkages (**Figure** **11**A),^[^ ^351^ ^]^ which are typically formed through the condensation of amines with aldehydes or ketones.^[^ ^352^ ^]^ The reversible nature of these bonds allows polyimines to undergo bond exchange reactions, imparting self‐healing, recyclability, and reconfigurability (for “renewable/reusable” device layouts) and thus resilience in service.^[^ ^351^ ^]^ Unlike traditional thermosets,^[^ ^353^ ^]^ which are permanently cross‐linked and non‐reprocessable, polyimines belong to a class of dynamic covalent thermosets that can be depolymerized and reconfigured under mild conditions—such as exposure to heat, specific catalysts, or reactive amines.^[^ ^354^, ^355^ ^]^ Initial efforts to incorporate polyimine chemistry into functional devices have focused on applications like electronic skins, where malleability, self‐healability (Figure 11B), and recyclability (Figure 11C) were demonstrated.^[^ ^355^ ^]^ Early systems were limited in stretchability and relied on inconvenient thermal pressing, motivating substrates that preserve 4R while carrying printed LM composites.

To address these shortcomings, recent advances have focused on a modified polyimine matrix that delivers significantly improved mechanical and electrical performance, while retaining full recyclability and dynamic functionality.^[^ ^205^ ^]^ This advancement has led to the creation of a multifunctional wearable electronic system by integrating rigid chips, eutectic LM interconnects deposited/patterned onto the polyimine substrate, and a dynamic polyimine matrix that provides stretchability, self‐healing, reconfigurability (renewable layouts), and closed‐loop recyclability in one platform (Figure 11C).^[^ ^355^ ^]^ The polyimine substrate enables bond exchange reactions, supporting autonomous healing and mechanical reconfiguration, while the LM circuitry ensures high conductivity and flexibility after cuts or deformation. A key feature is closed‐loop recycling: the polyimine matrix can be fully depolymerized in a methanol solution containing specific diamines (e.g., 3,3′‐diamino‐N‐methyldipropylamine, tris(2‐aminoethyl)amine),^[^ ^356^ ^]^ with complete dissolution in ≈30–47 min, allowing clean recovery of LM and chip parts for reuse, directly mitigating e‐waste. Recovered monomers/chips/LM can be repolymerized and reassembled into new devices with comparable performance across multiple cycles, reinforcing the 4R pathway.

In another study,^[^ ^352^ ^]^ a dynamic polyimine network with bulky pendant groups exhibited rapid bond‐exchange, room‐temperature self‐healing, and acid‐triggered full depolymerization to monomers, offering high mechanical robustness (resilience) and closed‐loop recyclability suitable for supporting printed LM conductors. The enhanced stability is attributed to aromatic conjugation (Figure 11D), providing resistance to hydrolysis and solvents while preserving the ability to recover monomers and relaminate devices for renewable/reconfigurable architectures. Such chemistries maintain strength without sacrificing recyclability, advancing sustainable LM‐on‐substrate systems.

Self‐healing mechanisms of polyimine in soft electronics arise from the dynamic covalent network, which enables bond‐exchange reactions (BERs) upon activation. In filler‐free systems, external heating reconnects damaged interfaces (Figure 11B), supporting structural resilience. For electrically active LM composites patterned on the substrate, polyimine can be combined with conductive fillers (e.g., MWCNTs) to enable Joule‐heat‐induced self‐healing: an applied current locally heats the cut region, activates BERs, and restores both polymer integrity and percolation (Figure 11E). As shown, a polyimine/MWCNT composite cut and rejoined under light pressure restored conductivity within minutes at ≈63 °C under 0.15 A,^[^ ^357^ ^]^ providing fast field‐repair (self‐healing) of LM circuits and extending device lifetime (resilience) without replacement. Polyimine‐based LM wearable electronics can be chemically recycled by immersion in amine/methanol (Figure 11F). The amines cleave imine bonds, depolymerizing the matrix into soluble oligomers/monomers in ≈30–47 min, after which chip components and LM (for direct redeposition/reprinting) are separated and recovered by simple filtration, reducing material loss and e‐waste.^[^ ^358^ ^]^ More broadly, introducing dynamic or cleavable linkers into thermosets (Figure 11G) enables reversible de‐/re‐crosslinking under heat, light, or acid, thereby facilitating component recovery and reuse in both polymer and fiber‐reinforced systems.^[^ ^359^, ^360^, ^361^ ^]^ These strategies establish a circular workflow for LM‐on‐substrate devices—supporting resilience in use, renewable/reconfigurable layouts, self‐healing after damage, and true recyclability—thereby advancing sustainability and measurably cutting e‐waste.

#### 6.1.3 Self‐Healing Polymers as Substrates in Stretchable Circuits

With the same importance as self‐healing of electrodes and LM‐based circuit traces, self‐healing of the substrate itself can also materially reduce e‐waste. Although reports that use self‐healing polymers specifically as LM substrates remain limited, the available studies establish both feasibility and clear mechanisms (**Table** **4**). Polymer self‐healing follows two modes. In extrinsic systems, microcapsules or vascular networks release healing agents when cracks form; in LM devices, these agents can re‐bond the substrate surface and help restore local LM adhesion.^[^ ^362^, ^363^ ^]^ In intrinsic systems, reversible bonds and chain mobility—such as imine exchange (transimination), disulfide metathesis, Diels–Alder adducts, and hydrogen‐bond networks—enable autonomous repair.^[^ ^364^, ^365^ ^]^ These intrinsic networks are well suited to LM circuits because mild external heating—or Joule heat from the LM trace—activates bond exchange, knitting the substrate while capillarity and LM fluidity re‐establish a continuous conductive path. For example, in a dynamic covalent polyimine platform, EGaIn interconnects are printed directly onto a polyimine film substrate and encapsulated with the same matrix; transimination heals cuts in service, and amine/methanol immersion (≈30–47 min) depolymerizes the substrate at end‐of‐life to release and reuse LM and chips, followed by repolymerization into new devices of comparable performance (Figure 11C).^[^ ^205^ ^]^ In a second implementation, a self‐healing polyurethane (PU) substrate carries screen‐printed LM/Ag FDs/SIS conductors; hydrogen‐bond networks and segmental mobility close fissures in PU while LM microdroplet coalescence and percolation restore conductivity (≈1.6 × 10⁵ S m^−1^; ≈99.84% healing efficiency), with sensor response maintained across cut–heal cycles.^[^ ^366^ ^]^ In a third example, a bio‐based α‐thioctic acid (TA) elastomer serves as the LM patterning bed; disulfide metathesis (assisted by mild or Joule heating) drives rapid mechanical self‐healing, while mask/plasma treatments raise local surface energy to selectively anchor LM, and a closed‐loop route then recovers substrate, LM, and chips, directly addressing e‐waste.^[^ ^314^ ^]^ In sum, despite the still‐limited number of reports, self‐healing polymers used explicitly as LM substrates already demonstrate mechanism‐grounded pathways to longer‐lived, repairable, and circular LM electronics that operationalize all four R's and measurably reduce e‐waste.

**Table 4:** Summary of self‐healing mechanisms in soft electronics, showing how polymer chemistry enables restoration of function based on requirements like flexibility, conductivity, and responsiveness.

Type of polymer	Healing mechanism	Details	Refs.
Supramolecular polymers	Non‐covalent interactions (e.g., hydrogen bonding, π–π stacking, metal‐ligand coordination)	Healing occurs through the reversible formation of non‐covalent interactions, allowing the polymer chains to reassemble and restore the structure.	[367, 368, 369]
Covalent adaptable networks (CANs)	Dynamic covalent bonding (e.g., Diels‐Alder reaction, transesterification)	These polymers use reversible covalent bonds that break and reform under external stimuli such as heat, light, or pressure, allowing the polymer to “heal” after damage.	[370, 371]
Elastomers (e.g., polyurethanes, polyesters)	Reversible cross‐linking	Cross‐links in the polymer network can break and reform upon the application of heat, light, or solvents, allowing for self‐healing after mechanical stress or damage.	[11, 20, 372, 373]
Ionic polymers	Ionic interactions	Ionic bonds within the polymer network can rearrange upon external stimuli (e.g., applied electric fields), leading to healing of cracks or damage.	[374, 375]
Thermoplastic polymers	Thermal healing	Heating the polymer above its glass transition temperature allows the polymer chains to flow and “heal” the damage.	[11, 367, 376]
Dynamically cross‐linked polymers	Thermally or chemically reversible networks	Cross‐links in these polymers can be made reversible by external stimuli such as temperature changes or exposure to specific chemicals. After damage, the cross‐links can re‐form to restore the polymer's mechanical integrity.	[377, 378, 379]
Hybrid polymers	Dual mechanism: supramolecular and covalent bonding	These polymers combine both supramolecular interactions and covalent cross‐links, offering self‐healing properties through the combination of ionic, hydrogen, and covalent interactions.	[22, 75, 380]
Conductive polymers	Reversible conductivity recovery	These polymers can heal their conductive pathways through reversible ionic or electronic interactions, restoring the electronic properties after physical damage.	[381, 382]
Photo‐responsive polymers	Photoinduced bond exchange	These polymers can heal using light as a stimulus, where light triggers the exchange of bonds (e.g., through photochemical reactions) to restore the polymer's structure.	[383, 384]
Shape memory polymers (SMPs)	Shape memory effect	These materials can “remember” a programmed shape and, when heated, revert to that shape, healing cracks or damage by restoring the polymer to its original form.	[385, 386, 387]
Thermo‐reversible polymers	Thermal‐induced healing via chain mobility	Certain polymers can self‐heal by allowing the polymer chains to reflow and interlock at elevated temperatures, facilitating healing of physical damage.	[388, 389]
Solvent‐assisted healing polymers	Solvent‐induced healing	Polymers like poly(methyl methacrylate) (PMMA) can undergo self‐healing when exposed to specific solvents, which break down damaged polymer regions and allow the damaged area to reassemble.	[11, 20, 390]
Autonomous healing via microcapsules	Microcapsule release (intrinsic healing)	Self‐healing materials can be embedded with microcapsules containing healing agents (e.g., epoxy resins). When damage occurs, the capsules rupture and release the healing agents to restore the polymer.	[391, 392, 393, 394]
Conductive hydrogels	Hydrogel‐based healing	These materials rely on the physical swelling and deswelling properties of hydrogels to heal cracks. Water absorption can restore mechanical integrity and even electrical conductivity in some cases.	[22, 75, 291]
Dual‐phase healing (thermo‐chemical)	Thermal and chemical dual healing	Combining both heat activation and chemical agents (such as catalysts) to initiate healing, this approach can be applied for both structural and electrical recovery.	[375, 395]
Magnetically actuated polymers	Magnetic field‐induced healing	Some polymers with magnetic properties can undergo self‐healing by manipulating magnetic fields, which cause the rearrangement of polymer chains or healing of microstructural defects.	[396, 397, 398, 399]

#### 6.1.4 Elastomers as Substrates in Soft Electronics

The rise of stretchable electronics has unlocked new possibilities for next‐generation technologies, including flexible circuitries, displays, energy devices, smart skins, electronic eye‐type imagers, and human‐interactive systems.^[^ ^18^, ^75^, ^400^, ^401^ ^]^ These systems must withstand bending, stretching, and conforming to complex surfaces while maintaining reliable performance. Among their required properties, stretchability remains the most challenging and technologically demanding—especially for wearable devices that integrate into fabrics or directly interface with skin. To address this, two key strategies have emerged: 1) engineering structural designs from conventional materials, and 2) developing intrinsically stretchable materials.^[^ ^18^ ^]^ Within the second category, elastomers such as PDMS, Ecoflex, SEBS, SIS, and TPU‐based materials are indispensable. As intrinsically stretchable polymers, they provide the mechanical compliance, durability, and recoverability needed for soft systems, making them the substrate of choice for LM‐based soft electronics. Critically, their compatibility with LM inks governs not only electrical performance but also recyclability, repairability, and reconfigurability under the 4R principle.

**Figure 12:**
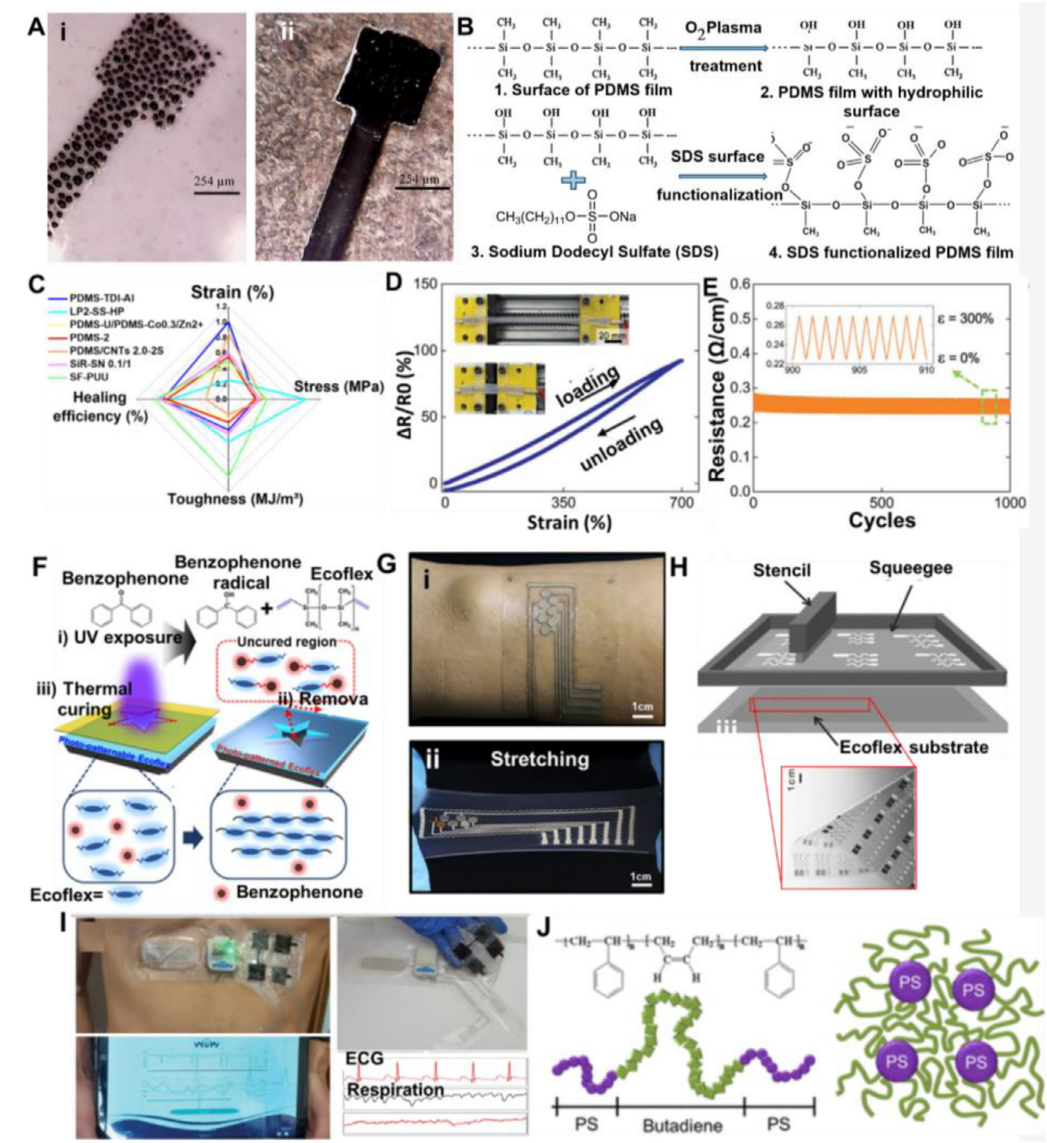
A) Silver nanoparticle (NP) ink printed on i) untreated PDMS and ii) plasma‐treated PDMS, demonstrating enhanced line clarity and adhesion due to surface treatment. Reproduced with permission,^[^ ^405^ ^]^ Copyright 2018, MDPI. B) Illustration of the synergistic effect of O_2_ plasma and SDS treatment on HT‐PDMS. Reproduced with permission,^[^ ^406^ ^]^ Copyright 2022, Elsevier. C) Comparative analysis of the self‐healing capability and mechanical properties of PDMS elastomers. D) Diagram showing conformational changes in the soft segment and cyclohexane ring under tensile stress. Reproduced with permission,^[^ ^425^ ^]^ Copyright 2020, Elsevier. E) Stress‐strain behavior of IBPU. Adapted with permission from the referenced source. Reproduced with permission,^[^ ^425^ ^]^ Copyright 2020, Elsevier. F) SEM images of graphene deposited on Ecoflex. Reproduced with permission,^[^ ^426^ ^]^ Copyright 2022, Nature. G, i) Fabrication of photo‐patternable Ecoflex (PPE), ii) its use as a wearable sensor, and iii) demonstration of stretchability up to 200% strain. Reproduced with permission,^[^ ^419^ ^]^ Copyright 2023, Nature. H) Schematic illustration of screen printing used to prepare the Ecoflex substrate and to pattern circuits on its surface. Reproduced with permission,^[^ ^420^ ^]^ Copyright 2015, Wiley. I) Chemical structure and bonding configuration of block copolymers such as styrene–isoprene–styrene (SIS). Reproduced with permission,^[^ ^423^ ^]^ Copyright 2018, ACS Publications.

PDMS is the most widely used elastomeric substrate due to its tunable mechanics (Young's modulus 0.5–3 MPa, elastic limit ≈200%), chemical stability, thermal resistance, and biocompatibility. Mature fabrication methods enable circuits with elongation up to 180% and thermal stability to 150 °C, ensuring reliable operation under repeated strain and heating.^[^ ^402^ ^]^ Importantly, PDMS has been successfully paired with LMs patterned on its surface. High‐resolution EGaIn circuits have been achieved on PDMS using spraying‐and‐wiping techniques, yielding feature sizes down to 50 µm.^[^ ^403^ ^]^ Similarly, micro‐transfer deposition enabled micron‐scale wiring,^[^ ^182^ ^]^ and layered PDMS–LM structures demonstrated in conformal antennas.^[^ ^404^ ^]^ These examples highlight PDMS as a robust platform for LM composites, enabling durable, high‐performance soft electronics. A persistent challenge, however, is PDMS's low surface energy, which hinders adhesion of LM inks. Surface treatments such as plasma etching significantly improve wettability and pattern fidelity (**Figure** **12**A).^[^ ^405^ ^]^ Beyond adhesion, mechanical mismatch also limits durability. Under stretching or cyclic strain, PDMS–LM devices often suffer delamination, cracking, or buckling from property differences between LM traces and the elastomer. For instance, LM‐embedded PDMS absorbers showed flexibility but degraded under repeated bending due to fatigue and interface debonding. To address this, interfacial functionalization has been pursued: polymeric interlayers, silane coatings, oxygen plasma, and surfactant treatments (e.g., SDS) improve surface energy and compatibility (Figure 12B).^[^ ^406^ ^]^ Complementary to surface treatments, self‐healing PDMS matrices provide an alternative route. While conventional PDMS lacks recyclability and toughness, a high‐performance variant using 2,4‐pentanedione with Al^3^⁺ coordination achieves superior toughness (48.73 MJ m^−3^) and >95% healing.^[^ ^407^ ^]^ Such matrices are attractive for LM systems, where both elastomer and LM traces can autonomously recover; for instance, LM–PDMS circuits restored >90% conductivity after repeated cuts, enabling durable wearable devices (ECG, motion, voice sensing).^[^ ^408^ ^]^ Figure 12C summarizes the healing and mechanical performance of PDMS elastomers.^[^ ^409^, ^410^, ^411^, ^412^, ^413^, ^414^, ^415^ ^]^ Finally, scalability and reproducibility remain hurdles: while microchannel molding and vacuum filling are effective, they are difficult to scale and prone to variable LM filling, weak PDMS bonding, and trapped bubbles. Additive manufacturing and stencil patterning offer more direct, scalable alternatives. Overall, PDMS remains a reliable platform for durable soft electronics, and advances in surface modification, self‐healing, and hybrid patterning are transforming adhesion, interfacial, and scalability challenges into opportunities, positioning PDMS–LM systems as key enablers for reducing e‐waste and advancing flexible technologies.

Similar to PDMS, Ecoflex serves as a widely adopted substrate for LM composites in soft electronics. Its ultralow modulus (≈70–200 kPa), >300% stretchability, and biocompatibility make it attractive for wearables, e‐skins, and soft robotics,^[^ ^416^ ^]^ where conformability and durability help reduce premature replacement and e‐waste. Diverse strategies have been developed to integrate LM: Au‐seeded wetting enables micron‐scale EGaIn patterning with stable conductivity under large strains,^[^ ^416^ ^]^ and PVP‐functionalized LM droplets form reconfigurable interface‐modified LM elastomer composites with a high stretchability and reliable performance over 5000 cycles (Figure 12D,E).^[^ ^417^ ^]^ Hybrid Ecoflex–LM systems with magnetic particles extend functionality into soft robotics,^[^ ^418^ ^]^ while photo‐patternable Ecoflex has been used to fabricate intrinsically stretchable multi‐sensors for sweat analysis (Figure 12F,G),^[^ ^419^ ^]^ and screen‐printed PEDOT:PSS inks on Ecoflex yield stretchable electrochemical devices (Figure 12H).^[^ ^420^ ^]^ Despite adhesion and recyclability challenges due to LM's high surface tension and encapsulation requirements, advances in surface modification and patterning continue to improve robustness and reusability, positioning Ecoflex–LM systems as sustainable, repairable, and multifunctional platforms for long‐lasting soft electronics.

Thermoplastic elastomers—TPU, SIS, and SEBS—are increasingly used as substrates for LM composites in soft electronics, pairing skin‐like mechanics with melt/solvent reprocessability that supports circular, lower‐waste designs. On TPU, multilayer EGaIn screen printing and stencil printing on electrospun membranes enable reliable stretchable NFC and breathable devices, with durability gains extending service life.^[^ ^421^, ^422^ ^]^ In another study, an LM–Ag–SIS composite ink printed on a TPU substrate produced a stretchable battery (Figure 12I), used for ECG monitoring; damaged electrodes were easily detached by immersing the device in acetone, enabling LM recovery.^[^ ^20^, ^21^ ^]^ Both SIS and SEBS are block copolymers (Figure 12J) that provide strong adhesion to LM inks,^[^ ^423^ ^]^ accommodate >1000% strain, and support EGaIn coatings that endure hundreds of bending/twisting cycles without measurable conductivity loss.^[^ ^212^ ^]^ Although ultrahigh elongation at break (>1000%) is often reported for soft electronic substrates, such extreme strain tolerance is unnecessary for most wearable applications. Human skin typically undergoes ≤30% strain, with localized peaks up to ≈60% during extreme motion.^[^ ^424^ ^]^ Substrates exceeding these values already provide a generous safety margin, while >1000% elongation should be regarded as a design buffer that enhances robustness and broadens application space. Their intrinsic self‐healing is also beneficial^[^ ^21^, ^40^ ^]^: an LM‐based battery on an SIS substrate showed electrode self‐healing because SIS served as both substrate and binder in the LM–polymer composite used for the current collector and active layers.^[^ ^21^ ^]^ At the end‐of‐life, thermoplastic/solvent processability enables substrate removal and LM recovery,^[^ ^21^ ^]^ directly reducing e‐waste. Despite these advantages, adoption remains largely at the laboratory scale; scaling manufacturing, identifying greener solvent systems to replace toluene and DMF for SIS/SEBS processing, and standardizing recovery protocols are key next steps.

#### 6.1.5 Hydrogels as Substrates in Soft Electronics

Hydrogels, with their soft, water‐rich composition, are promising substrates for diverse applications in soft electronics and bioelectronics.^[^ ^75^ ^]^ These 3D networks, chemically or physically cross‐linked, retain water without dissolving and offer key properties such as biocompatibility, biodegradability, elasticity, and hydrophilicity. Their exceptional flexibility and stretchability make them ideal for overcoming challenges in traditional elastomer‐based approaches, particularly by reducing mechanical mismatch and stress concentration between rigid conductors and soft substrates.^[^ ^427^ ^]^ They possess unique properties suitable for human interaction, such as softness resembling biological tissues, but they often lack the necessary mechanical robustness.^[^ ^75^, ^291^ ^]^

Most hydrogels contain irreversible covalent cross‐links that prevent self‐repair, posing challenges in maintaining mechanical properties over time.^[^ ^428^ ^]^ Self‐healing hydrogels offer a solution to this limitation.^[^ ^429^ ^]^ Self‐repairing hydrogels possess the remarkable ability to regenerate after damage, primarily through dynamic covalent cross‐links and a combination of covalent and non‐covalent interactions. Drawing inspiration from natural organisms, these materials initiate self‐repair in response to external stimuli or specific interactions, thereby preserving their structural and functional integrity.^[^ ^430^ ^]^ This self‐healing capacity allows them to withstand external mechanical stresses, making them particularly suited for demanding biomedical applications such as tissue engineering, wound healing, drug delivery, and regenerative medicine.^[^ ^431^ ^]^

In addition to their regenerative ability, these hydrogels often demonstrate multifunctional properties, including electrical conductivity, rapid adhesion, responsiveness to environmental stimuli, and adequate mechanical strength, all of which are essential for advanced biomedical technologies.^[^ ^432^ ^]^ The repair process closely mimics biological systems, involving steps such as surface rearrangement, interfacial joining, wetting, and molecular‐level interactions. These mechanisms collectively enable the regeneration of broken bonds through reversible physical and chemical interactions.^[^ ^433^ ^]^ Furthermore, the incorporation of non‐covalent bonds—such as ionic and hydrogen bonds—significantly enhances the efficiency and speed of the self‐healing process. This synergistic use of reversible bonding strategies underpins the robust, adaptive performance of self‐repairing hydrogels in complex physiological environments.^[^ ^434^ ^]^

**Figure 13:**
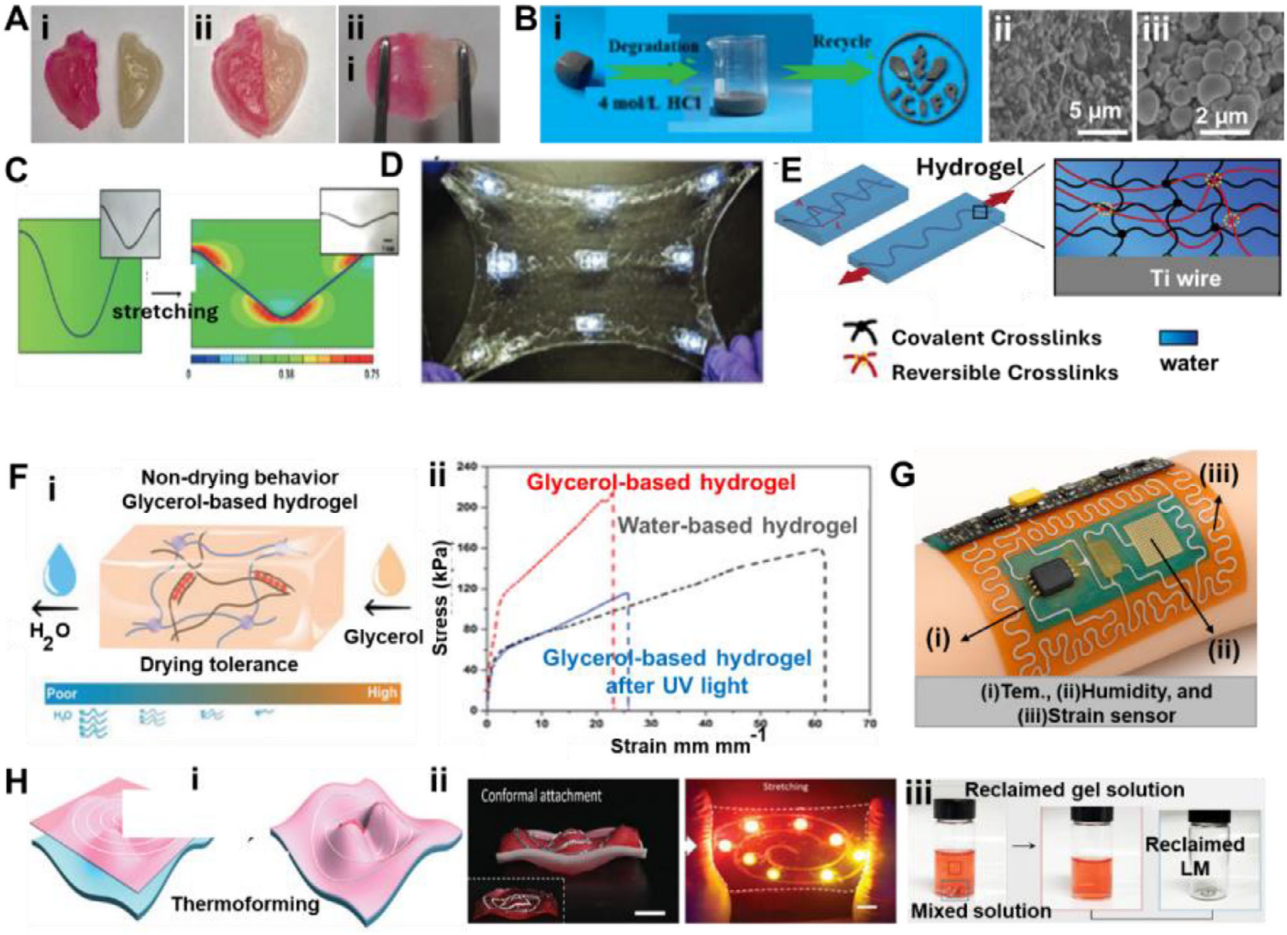
A) Macroscopic observation of the self‐healing process of the borax cross‐linked hydrogel. Reproduced with permission,^[^ ^435^ ^]^ Copyright 2023, Elsevier. B) Recycling process of the LMCNF‐2 hydrogel placed in 4 mol L^−1^ HCl solution and deionized water (i). i, ii) Representative SEM images of particles obtained from the gray slurry of the LMCNF‐2 hydrogel and the recycled LMCNF‐2 hydrogel, respectively. Reproduced with permission,^[^ ^438^ ^]^ Copyright 2023, Elsevier. C) Finite‐element analysis depicting the maximum principal strain within the hydrogel matrix during the stretching of hydrogel‐based soft electronics (HSE). Reproduced with permission,^[^ ^440^ ^]^ Copyright 2015, PMC. D) Hydrogel‐based soft electronics integrated with distributed functional components for temperature monitoring and controlled drug release. Adapted with permission from. Reproduced with permission,^[^ ^440^ ^]^ Copyright 2015, PMC. E) Stretchable HSE incorporating wavy metal interconnects to maintain conductivity under deformation. Reproduced with permission,^[^ ^440^ ^]^ Copyright 2015, PMC. F, i) Structural representation of alginate/PAAm double‐network hydrogels, featuring covalent crosslinking of PAAm via a photocleavable ONB‐PEG 600 crosslinker and ionic crosslinking of alginate through Ca^2^⁺ in aqueous media. ii) Mechanical characterization through stress‐strain analysis of the DN hydrogel. Reproduced with permission,^[^ ^19^ ^]^ Copyright 2023, Wiley. G) Multifunctional hydrogel‐based soft electronics capable of detecting temperature, humidity, and mechanical strain, with color mapping representing variations in hydrogel modulus. Adapted with permission from. Reproduced with permission,^[^ ^441^ ^]^ Copyright 2020, Nature. H, i) Schematic and photographic demonstration of the thermoforming process used to shape hydrogel‐based soft electronics for conformal contact on curved surfaces. ii) Printing LM over hydrogel substrate, and iii) Demonstration of recyclability by disassembling a hydrogel–LM antenna at 60 °C for 30 min, enabling separation and reuse of hydrogel and LM components. Reproduced with permission,^[^ ^447^ ^]^ Copyright 2022, Wiley.

These initial studies suggest that the choice of initial materials is critical for the fabrication and recyclability of self‐repairing hydrogels. For example, Borax, which forms dynamic boronic ester bonds with diols, is widely used due to its rapid conjugation and dissociation properties (**Figure** **13**A).^[^ ^435^, ^436^ ^]^ A recent example of a material system that incorporates borax is a self‐healing, electrically conductive organogel composed of PVA, borax, and ethylene glycol.^[^ ^437^ ^]^ The organogel is embedded with a percolating network of LM droplets and silver flakes and exhibits both electrical and mechanical self‐healing. When torn or cut, the gel self‐adheres under light external pressure and regains most of its electrical conductivity and mechanical integrity.

Dynamic boronic ester bonds play a key role in hydrogel recyclability by disrupting the 3D network structure in acidic environments. For example, in sulfuric or hydrochloric acid, boronic ester bonds undergo hydrolysis, enabling the recovery of LM.^[^ ^438^, ^439^ ^]^ Under these conditions, the hydrogel dissolves as the acidic medium breaks the diol–boronic acid complexes (Figure 13B). SEM and EDS elemental mapping confirmed the recovery of spherical LM particles with a uniform distribution of Ga, In, O, and C. Additionally, HRTEM images verified the disruption of the borax crosslink network, facilitating effective LM particle recovery.^[^ ^438^ ^]^

Another challenge in soft substrates like hydrogels is the large mismatch in Young's modulus when combined with metal conductors, leading to stress concentration at the interface during deformation. This can cause interface failure or fragmentation of the hydrogel matrix (Figure 13C). To address this, several strategies have been employed:

1. Tough hydrogels as a substrate: Using tough hydrogels with excellent mechanical properties can significantly enhance the reliability and stretchability of the substrate. For example, polyacrylamide‐alginate (PAAm‐alginate) hydrogels have been utilized as substrates for stretchable multifunctional soft electronics, enabling reliable bonding between the hydrogel matrix and surface‐modified metal interconnects (Figure 13D). This improves the overall stretchability and stability of the system, making it suitable for applications like drug delivery and temperature sensing. These systems provide a simple approach to designing stretchable and multifunctional soft electronics using tough hydrogel substrates and surface‐modified metal conductors.^[^ ^440^ ^]^
2. Forming robust interfaces with rigid components: Enhancing the interface interactions between the rigid conductor (metal wires) and the hydrogel substrate (Figure 13E) is essential for improving the performance of soft electronics. Surface modification of metal wires, such as silanization, allows for a more robust bond with the hydrogel matrix, thereby enabling high stretchability and stable functionality. The integration of such surface‐modified titanium wires with a polyacrylamide‐alginate (PAAm‐alginate) hydrogel substrate shows the importance of improving interface bonding for high‐stretchable devices.^[^ ^440^ ^]^
3. Improving interfacial adhesion to patterned soft circuits: Printing biphasic LM‐Ag flake‐SIS composites onto hydrogel substrates poses challenges due to poor adhesion and delamination. To address these issues, surface treatment of the hydrogel substrate is necessary to enhance interfacial bonding and ensure stable ink deposition. In one study, photodegradable hydrogels (Figure 13F‐i) were developed as advanced substrates for sustainable electronics, exhibiting key properties such as robustness, ink adhesion, and controlled degradation. These hydrogels utilize a dual crosslinking strategy: reversible ionic interactions between sodium alginate and Ca^2^⁺ provide structural stability, while light‐responsive crosslinking of poly(acrylamide) (PAAm) using ortho‐nitrobenzyl (ONB)‐based crosslinkers enables degradability. To mitigate drying and printability challenges, water within the hydrogel is replaced with glycerol, maintaining flexibility and improving ink adhesion. This modification enables the successful digital printing of LM‐based stretchable ink, facilitating the fabrication of body‐worn sensors. Moreover, these hydrogels allow for the controlled degradation and recycling of both LM particles and the hydrogel component, establishing them as a promising platform for next‐generation eco‐friendly electronics. Furthermore, the hydrogel shows higher mechanical properties copared to the other composites (Figure 13F,ii).
4. Modulation of Substrate Stiffness: Another innovative approach to reduce stress concentration is spatially modulating the mechanical properties of the substrate. For instance, strain can be concentrated in soft areas of the substrate, while harder areas exhibit minimal deformation (Figure 13G). This strategy has been applied in multimodal soft electronics, where a hydrogel matrix with varying stiffness is used. Different regions of the hydrogel substrate are designed to be soft (for strain sensing) or hard (for temperature and humidity sensing), thus preventing damage and improving the overall functionality of the device. This design increases the sensitivity of the strain sensor while preserving the accuracy of temperature and humidity sensors in the soft electronics.^[^ ^441^ ^]^
5. Localized Stiffening: The stiffness of the hydrogel matrix can also be modulated locally by post‐treatment processes, such as crosslinking with multivalent ions in specific regions where interconnects or IC chips are integrated. This technique has shown promise in enhancing the stretchability of the device without compromising functionality. For example, a PAAm‐alginate hydrogel substrate with locally stiffened regions can be stretched up to 150% strain without component detachment or functional degradation.^[^ ^442^ ^]^

Another use of hydrogels is in conforming 2D planar soft electronics to 3D curvilinear surfaces with non‐Gaussian curvatures, which remains challenging due to mechanical mismatches. Existing strategies, such as kirigami patterning or ultrathin substrates improve conformability but often compromise structural integrity.^[^ ^443^, ^444^, ^445^ ^]^ This limitation is addressed using sustainable hydrogel‐based soft electronics (HSE), integrating sensing elements and patterned LM within a gelatin–alginate hybrid hydrogel. The hydrogel is transparent, robust, resilient, and recyclable. The HSE is multifunctional, capable of sensing strain, temperature, pH, and heart rate (ECG), with strain sensitivity sufficient to detect a human pulse. Additionally, it enables iontophoretic drug delivery and noncontact sensing via electrostatic‐field‐induced voltage. Both LM and hydrogel are healable, degradable, and recyclable, supporting sustainable device reuse and reconfiguration. To ensure conformal integration with 3D surfaces, the HSE is endowed with thermoforming capability via the sol‐gel transition of the hydrogel and the deformability of LM.^[^ ^446^ ^]^ As shown in Figure 13H(i), the planar HSE initially adheres only to convex regions. After heating at 60 °C for 5 min, it softens and conforms seamlessly to the surface. The printed LM composite also functions as an antenna. Upon further heating at 60 °C for 30 min, the hydrogel liquefies and the LM aggregates (Figure 13H,ii), enabling full material recovery (Figure 13H,iii) and reuse.^[^ ^447^ ^]^

These strategies highlight the significant role hydrogels play as substrates in soft electronics, providing a balance between mechanical flexibility, electrical conductivity, and structural integrity. Future developments in this field should focus on further enhancing the adhesion between soft and hard interfaces, addressing issues related to corrosion in aqueous environments, and exploring scalable fabrication techniques for integrating rigid IC chips into hydrogel matrices.

By combining these advanced approaches and design strategies, it is possible to fabricate highly flexible substrates that support LM‐based circuits. These substrates can support soft and stretchable circuits with metal‐like conductivity, easy patternability, and robust interfacing with surface‐mounted microelectronics. The development of such substrates is crucial for the future of soft electronics, enabling the creation of devices that are not only highly functional but also capable of withstanding significant mechanical deformations. This progress represents a significant step forward in the integration of commercial electronic components into flexible and stretchable devices, paving the way for a new generation of advanced electronic applications.

## 7 Sustainable Recycling of Composite Inks and Stretchable/Flexible Circuits for Liquid Metal Recovery

As the demand for sustainable electronics grows, efficient recycling of LM, such as gallium and indium, from soft electronic devices is becoming increasingly vital. These metals are finite and valuable; recovering them reduces environmental impact, conserves natural resources, and aligns with circular economy principles by keeping materials in use and minimizing waste. Recycling LM offers both ecological and economic benefits. In principle, it can lower greenhouse gas emissions and be more energy‐efficient than mining and refining new metals, since primary Ga and In production is highly energy‐intensive due to their low natural abundance and reliance on byproduct extraction.^[^ ^448^, ^449^, ^450^ ^]^ Lab‐scale studies have demonstrated high LM recovery yields (≈95–98%) and reclaimed alloys with compositions and functional performance comparable to pristine LM, enabling reuse in circuits and sensors.^[^ ^20^, ^21^, ^83^ ^]^ However, current LM recycling approaches—especially for soft, multi‐material systems—remain chemically and energy intensive, and comprehensive life‐cycle advantages over primary mining and refining have yet to be demonstrated at scale. Moreover, reclaiming LM lowers production costs and supports the long‐term viability of electronic manufacturing.

In general, existing LM‐based stretchable and flexible circuits—often composed of LM, metal powders, and polymer matrices—pose significant recycling challenges due to their complex material interactions. Traditional recovery methods, such as acid or solvent‐based extractions, can be inefficient and environmentally harmful.^[^ ^451^ ^]^ Recent research focuses on eco‐friendly alternatives. Aqueous systems, including Epsom salt, sugar‐based solutions, and deep eutectic solvents, have demonstrated high efficacy in separating LM droplets and metal particles while minimizing environmental risk.^[^ ^19^, ^20^, ^21^ ^]^ Additionally, solvent‐vapor‐assisted decomposition techniques are being explored for the selective recovery of components from e‐textiles and hybrid electronic patches. Overall, developing effective, environmentally responsible recycling strategies for LM‐based soft electronics is crucial. It enables material reuse, reduces e‐waste, and contributes to a more sustainable future in electronic device manufacturing.

### 7.1 Solvent‐Assisted Vapor Decomposition and Recovery Method

**Figure 14:**
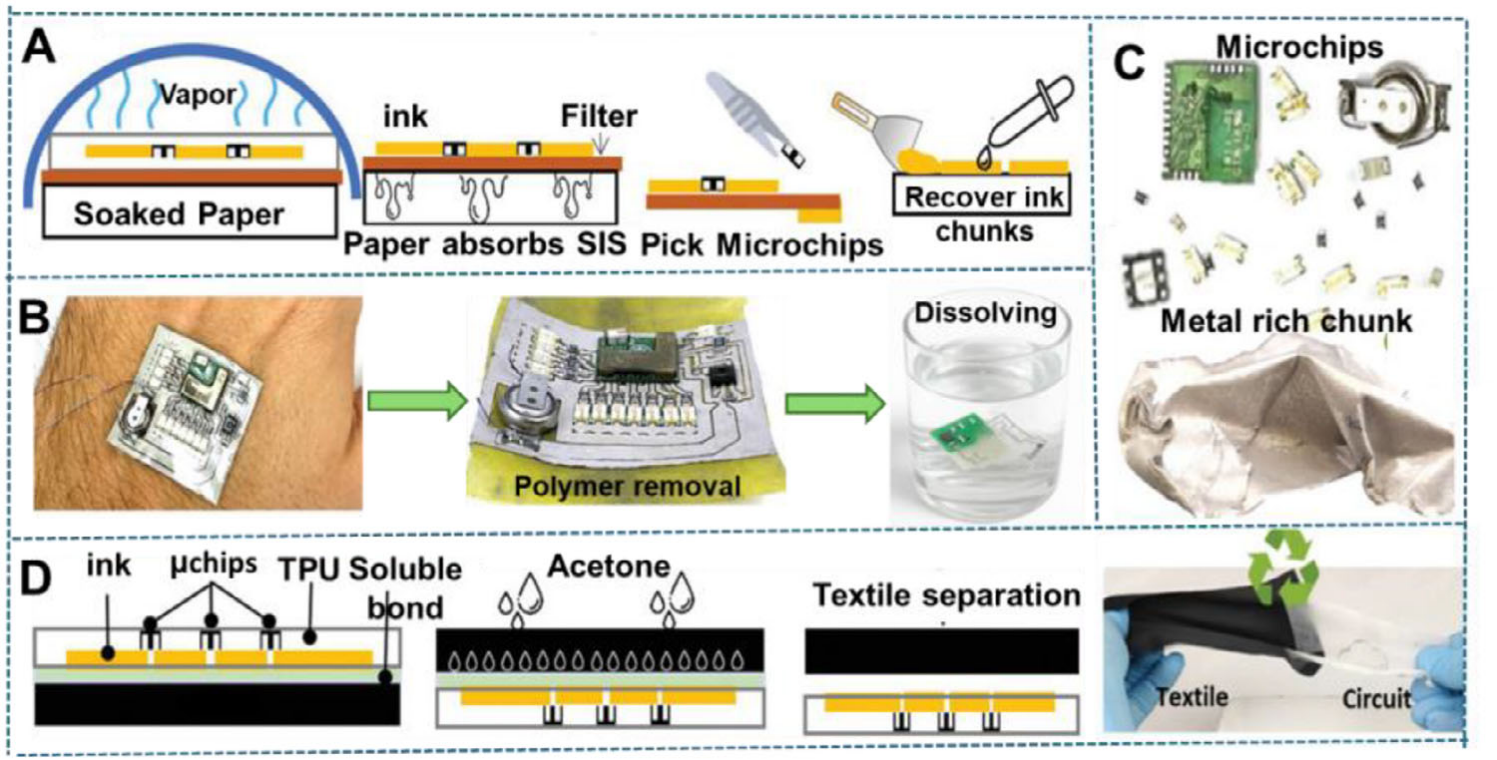
A) Schematic illustration of the Solvent‐Assisted Vapor Decomposition and Recovery Method for recycling liquid metal (LM) and other components from electronic circuits. B) Separation of circuit components by dissolution in a solvent. C) Recovered and separated components. D) E‐textile composition and reconstruction of the circuit. Reproduced with permission,^[^ ^11^ ^]^ Copyright 2022, Wiley.

Solvent‐assisted vapor decomposition facilitates the efficient separation and recovery of components from e‐patches and e‐textiles by leveraging a solvent vapor environment. A solvent‐soaked paper placed within a sealed chamber generates vapors that dissolve the substrate, enabling material recovery through a combination of vapor‐assisted removal and direct dissolution.^[^ ^11^ ^]^ As shown in **Figure** **14**, both e‐textiles and hybrid patches can be effectively recycled using this approach. The process involves placing the electronic circuit on a filter above a toluene‐soaked paper inside a closed chamber. The solvent vapor dissolves the SIS substrate, allowing the ink and electronic components to separate. This technique enables the recovery of high‐value microchips and produces metal‐rich fragments containing EGaIn, metallic microfillers (e.g., Ag or Fe), and residual SIS polymer.^[^ ^19^, ^62^ ^]^ For e‐textiles, the decomposition process involves dissolving adhesive polymers, such as SIS or TPU, which are commonly used to fuse patches onto fabric through heat or solvent‐vapor‐assisted fusion. Recycling is achieved by soaking the e‐textile in a solvent to detach the patch from the fabric. Further treatment with NaOH or HCl solutions may be necessary to fully recover LM particles, which will be discussed in the following.

### 7.2 Chemical Dissolution Methods for LM Extraction

Chemical dissolution methods use selective solvents to extract LM from electronic devices while preserving other materials. Acids, bases, and complexing agents are commonly employed, each targeting specific components. For instance, hydrochloric acid (HCl) and sulfuric acid (H_2_SO_4_) can dissolve metal substrates, releasing LM for recovery. However, precise control of reaction conditions—such as temperature and concentration—is essential to prevent damage to sensitive components and optimize recovery efficiency. Additionally, proper waste management is crucial to mitigate the environmental impact of chemical byproducts.

#### 7.2.1 Water‐Triggered Recovery of Liquid Metal from LM Composites for Recycling and Reuse

The water‐degradable stretchable electronics system represents a cutting‐edge innovation designed to not only enhance the eco‐friendliness of electronic devices but also provide recyclability and resource recovery. This system strives to combine biodegradability for effortless disposal, recyclability for maximum material recovery, and stretchability to withstand mechanical deformation. The latter is particularly crucial in wearable electronics, where the device must endure the dynamic movements and strains of human skin, which can stretch up to 100% during daily use.^[^ ^452^ ^]^ The escalating problem of electronic waste has driven significant interest in the development of transient circuits and recyclable electronics, as these technologies offer promising solutions to mitigate environmental damage. Among the most innovative approaches is the use of water‐soluble electronics, which not only create transient circuits but also provide an effective mechanism for LM recovery and recycling. These transient electronics are designed to disintegrate upon exposure to specific triggers, such as water or heat, promoting a more sustainable lifecycle for electronic devices. By utilizing water‐soluble substrates, the recovery of LMs becomes much more efficient, as the dissolution process allows for the easy separation and reuse of valuable components. Such technologies play a crucial role in reducing waste and promoting the sustainable use of LMs in various applications.^[^ ^137^, ^204^, ^453^ ^]^

**Figure 15:**
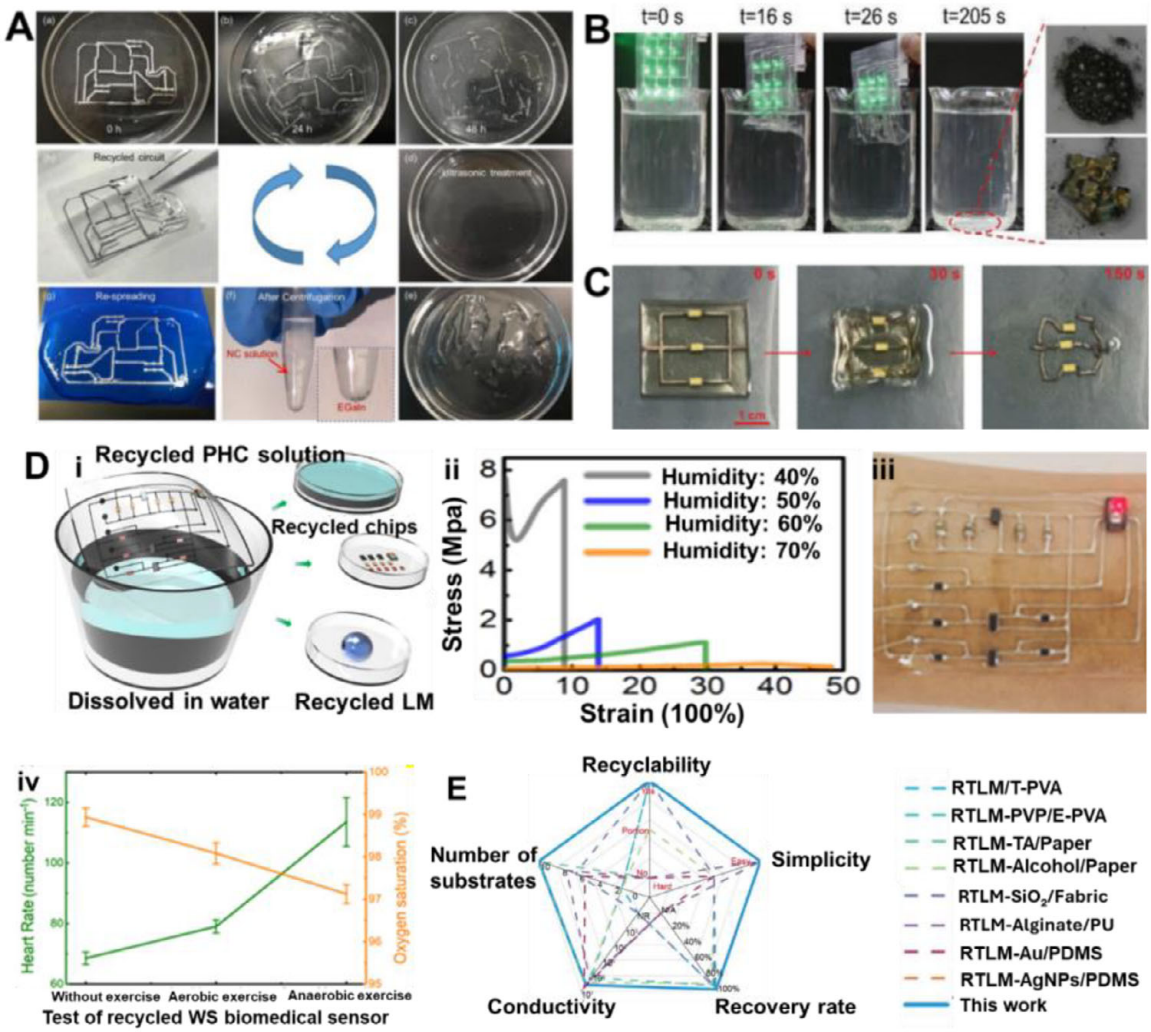
A) Dissolution and reuse of the NC‐LM circuit: i) immersion in water; ii) fragmentation after 24 h; iii) blurred LM pattern after 48 h; iv) ultrasonic‐assisted degradation; v) uniform NC‐LM mixture; vi) LM and NC separated by centrifugation; vii) NC re‐applied; viii) recycled circuit with original morphology. Reproduced with permission,^[^ ^455^ ^]^ Copyright 2021, Elsevier. B) Dissolution and recycling process of the double‐layer LED array on PVA/fructose film. Reproduced with permission,^[^ ^137^ ^]^ Copyright 2019, Wiley. C) Digital photos of the degradation of a gelatin hydrogel‐encapsulated LM circuit in hot water (65 °C). Reproduced with permission,^[^ ^456^ ^]^ Copyright 2022, ACS. D) Degradation and recovery of LMs from a water‐degradable PVP‐honey composite stretchable biomedical sensor (i). Comparison of the stress‐strain behavior of native, self‐healing, and recycled PHC V films. Reproduced with permission (ii). Rebuild sensor (iii). Sensor for monitoring heart rate and blood oxygen saturation (iv). Reproduced with permission,^[^ ^457^ ^]^ Copyright 2024, Wiley. E) Radar plot comparing the flexible lignin‐encapsulated EGaIn particle electronic device with other reported RTLM‐based systems. Reproduced with permission,^[^ ^231^ ^]^ Copyright 2024, Wiley.

A particularly noteworthy advancement in recyclable electronics comes from a British startup (Jiva Materials) that has developed the only commercially available fully recyclable material for PCB substrates. This new material serves as an alternative to traditional PCBs made from fiberglass and epoxy. Composed of natural fibers and a halogen‐free polymer, the material undergoes delamination when immersed in hot water, breaking down into natural fibers that can either be composted or reused. This process allows for the recovery of ≈90% of the electronic components for recycling.^[^ ^286^, ^454^ ^]^ While this material may not be suitable for all applications—particularly those requiring the rigid capabilities of traditional PCBs—it holds immense potential for short‐lived products. Its natural degradation once exposed to water also makes it ideal for environmentally sensitive applications. For instance, PCBs could be used in environmental sensors placed in nature reserves, where they would naturally dissolve once their operational life concludes, leaving behind no lasting environmental impact.^[^ ^286^ ^]^ One promising solution is the nanocellulose (NC)‐LM circuit—a game‐changing approach combining water‐degradable NC film and LM. As the circuit is immersed in water, the NC film begins to dissolve within 48 h, with ultrasonic shaking speeding up the process. As the film breaks apart, LM droplets separate and coalesce, creating the perfect opportunity for centrifugation to recover both the LM and NC. These components can be reprocessed and reused in new circuits, with their morphology and functionality preserved (**Figure** **15**A).^[^ ^455^ ^]^ The process showcases the recyclability of this system, creating a new paradigm for sustainable electronics.

In line with this, the use of PVA, a water‐soluble polymer, is emerging as a key component in the development of flexible, biodegradable circuits. PVA is known for its biocompatibility, film‐forming properties, and ability to dissolve in water, making it an excellent substrate for transient electronics. A recent study demonstrated the potential of PVA‐based circuits in flexible applications by printing the word “LM” using PVA‐LM ink and immersing it in a 1 m NaOH solution. After agitation, the LM agglomerated, and the material retained its fluidity and conductivity. This agglomerated LM was then reconstituted into PVA‐LM ink and reprinted onto a polyurethane (PU) film, ultimately serving as an electronic tattoo, showcasing both its flexibility and biodegradability.^[^ ^126^ ^]^ In another study, the PVA/fructose film, known for its rapid dissolution in water (25 °C), breaks apart the Fe‐EGaIn circuits, turning off LEDs in less than 205 s. Following dissolution, the LM droplets are recovered and purified using an HCl solution to remove oxides and restore their shape. Remarkably, the system mimics real‐life conditions by rapidly degrading under simulated rain, confirming that the dissolution rate can be controlled for precise degradation in transient electronics (Figure 15B).^[^ ^137^ ^]^ In an exciting twist, an LED circuit on a 15 wt% gelatin hydrogel film undergoes deformation within 30 s, completely dissolving in hot water at 65 °C within just 150 s. The LED chips are easily retrieved, and the LM interconnects are efficiently recycled using either acidic or alkaline solutions (Figure 15C).^[^ ^456^ ^]^ This demonstrates not only the quick dissolution of transient circuits but also the reusability of essential components, laying the foundation for a new era of sustainable electronics.

Another potentially groundbreaking development comes from the PVP‐honey composite stretchable biomedical sensor, where water‐triggered dissolution allows for the recovery of LM after the sensor degrades. The PVP‐honey composite dissolves in water at a controlled rate, enabling the separation and purification of the LM traces for reuse in new sensor fabrication (Figure 15D,i).^[^ ^457^ ^]^ This recycling process has the potential to revolutionize flexible, stretchable sensor applications, providing sustainability without compromising performance. In terms of mechanical resilience, the stress‐strain behavior of pristine, self‐healing, and recycled PHCV films was compared (Figure 15D,ii).^[^ ^457^ ^]^ The results revealed that the self‐healing and recycled films exhibited mechanical properties that were nearly identical to the original films. This demonstrates the self‐repairing nature of the material, even after multiple cycles of degradation and recycling, reinforcing the durability and longevity of the sensors. Moreover, the recycled LM was successfully used to rebuild a sensor (Figure 15D,iii).^[^ ^457^ ^]^ The rebuilt sensor performed just as effectively as the original, exhibiting comparable sensitivity and functionality (Figure 15D,iv). This success underscores the fact that the recycling process did not compromise the device's performance, proving the feasibility of using recycled LMs in the fabrication of stretchable biomedical sensors. Not only is this process sustainable, but it also maintains high functionality, paving the way for more eco‐conscious innovations in electronics.

Figure 15E features a radar plot comparing features of a flexible electronic device composed of lignin‐encapsulated EGaIn particles with other LM‐based systems.^[^ ^231^ ^]^ The recycling process for the lignin‐encapsulated composite demonstrates unprecedented efficiency, recovering 96.9% of the LM, with minimal losses (only 3.1% during the washing and transfer stages). The lignin encapsulation serves as a protective barrier, providing oxidation resistance and UV protection, which significantly boosts recycling performance. This system's ability to form various circuit designs adds versatility, while the high recovery rate ensures that the LM can be reused in new applications. In sum, this water‐triggered recovery system represents a major leap forward in the sustainable development of electronics. By enabling recycling and reusing valuable materials like LMs, this innovation reduces environmental impact and offers a practical solution for managing the lifecycle of transient electronic devices. The integration of biodegradability, recyclability, and high performance in these systems shows immense promise, ushering in a new era of environmentally friendly and economically viable electronics.

#### 7.2.2 Alcohol‐Assisted Recovery of LM Components from Soft Electronics

**Figure 16:**
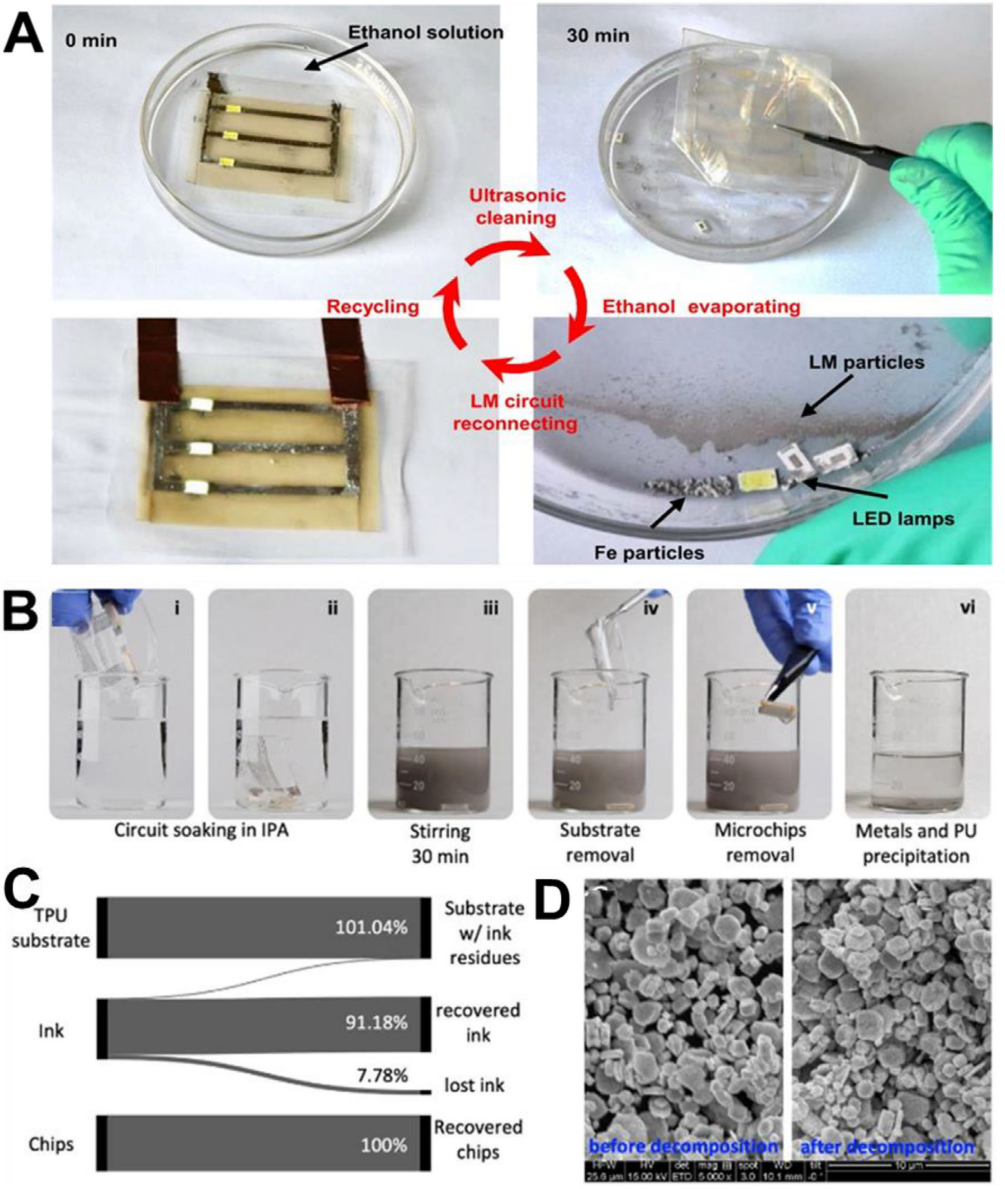
A) Schematic illustration of the process of LM recovery from a Fe‐LM composite patterned on a silicon substrate. Upon ultrasonic treatment in ethanol, the LM disperses into fine particles. After ethanol evaporation, the residual LM particles and electronic components are collected and reused, demonstrating the recyclability of the composite system. Reproduced with permission,^[^ ^458^ ^]^ Copyright 2021, IOP. B) Circuit degradation and separation process. The circuit is soaked in IPA (i, ii), and stirred for 30 min (iii), and the clean TPU substrate (iv) and rigid components (v) are removed. Ag flakes and PU residues precipitate (vi), are decanted, and the remaining IPA is evaporated. Reproduced with permission,^[^ ^459^ ^]^ Copyright 2023, Wiley. C) Separation efficiency. Rigid components and TPU are fully recovered with minimal ink residue, and ink (Ag flakes and PU) is separated with ≈90% efficiency, considering losses during decanting. D) SEM image of Ag flakes showing no morphological changes before and after separation (scale bar = 10 µm). Reproduced with permission,^[^ ^459^ ^]^ Copyright 2023, Wiley.

Alcohol‐based solvents, particularly ethanol and isopropyl alcohol (IPA), have shown great potential in the recovery of LM from electronic waste, specifically from ink traces and composite materials in soft electronics. These solvents provide a selective disassembly method that enables the efficient recovery of valuable materials, including LM inks, conductive substrates, and rigid components, without the need for harsh chemicals or energy‐intensive processes. This approach is not only eco‐friendly but also aligns with the growing demand for recyclable and sustainable electronic materials. In one work, a stretchable electronic circuit was fabricated by printing Fe‐EGaIn on a laser‐patterned silicone substrate. The device exhibited rapid disintegration, breaking apart within 30 minutes under ultrasonic treatment in an ethanol solution.^[^ ^458^ ^]^ This behavior is attributed to the ability of EGaIn to disperse into fine particles when subjected to ultrasonication in ethanol. Following ethanol evaporation, the remaining EGaIn particles and LED components were successfully recovered and reused, demonstrating the recyclability of the system (**Figure** **16**A).^[^ ^458^ ^]^ Moreover, IPA has proven to be an effective, low‐toxicity solvent for recycling LM‐based soft electronic circuits. In particular, circuits made with silver–waterborne polyurethane (Ag‐WPU) inks on TPU substrates can be efficiently deconstructed through a solvent‐assisted dissolution process.^[^ ^459^ ^]^ As shown in Figure 16B(i–vi), the circuit is immersed in IPA and agitated at 500 RPM for 30 minutes. During this process, IPA selectively dissolves the Ag‐WPU layer—dispersing silver flakes and LM particles—while leaving the TPU substrate and rigid surface‐mounted components (SMDs) intact. This selectivity arises from the structural differences between TPU and WPU: TPU's high crosslinking density resists dissolution and only swells in IPA, whereas WPU, with its polar groups and lower crosslinking, dissolves readily. After agitation, solid particles settle within an hour, allowing the supernatant IPA to be decanted and evaporated, leaving behind recoverable LM ink. This method achieves a high recovery rate—about 91.18% of the original conductive ink—with minimal residue on the components. The separated TPU and SMDs can be reused after simple rinsing, and SEM analysis (Figure 16D) confirms the preserved morphology of the silver flakes, supporting their reuse. Overall, this IPA‐based approach enables efficient, selective, and eco‐friendly recycling of LM‐based soft electronics without harsh chemicals or high‐energy input, aligning with sustainable material design and circular electronics principles.

#### 7.2.3 Toluene‐Mediated Disassembly and Recycling of LM‐Based E‐Waste

**Figure 17:**
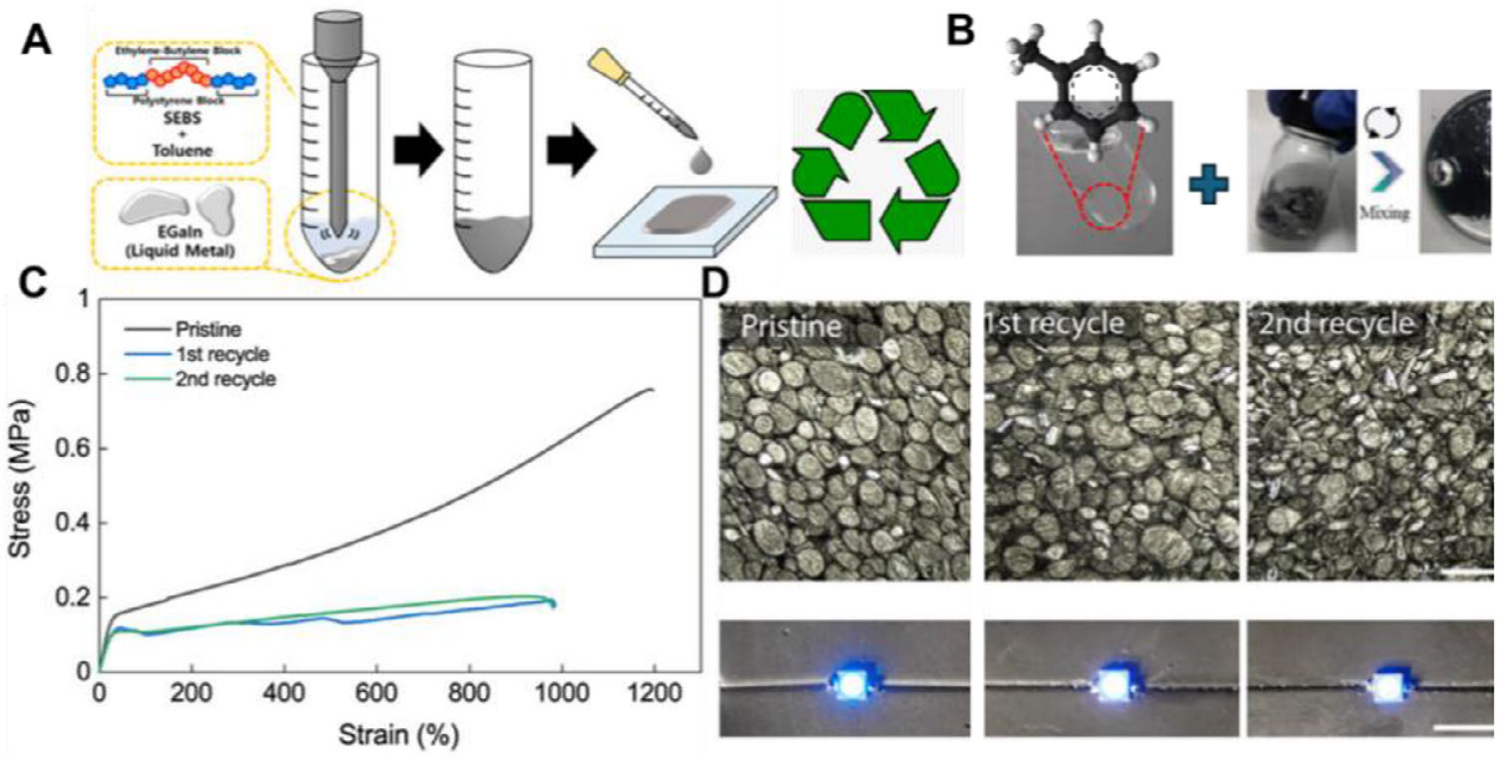
A) Schematic illustration of the tip sonication process for fabricating the EGaIn–SEBS composite by dissolving the SEBS polymer in toluene. Reproduced with permission,^[^ ^460^ ^]^ Copyright 2022, MDPI. B) Sonication of LM‐based composite waste in toluene followed by recovery of LM droplets. C) Stress–strain curves of the pristine and recycled samples indicate higher stress levels for the pristine material. D) Optical micrographs reveal changes in the liquid metal droplet microstructure after recycling, while the LEDs demonstrate retained electrical functionality in the composite with ϕ  =  60% and δ  =  10%. Scale bars: 100 µm for micrographs, 10 mm for LED images. Reproduced with permission,^[^ ^40^ ^]^ Copyright 2021, Nature.

Toluene is a widely used organic solvent that plays a key role in both the fabrication and recycling of LM‐based polymer composites (**Figure** **17**A,B).^[^ ^460^ ^]^ During fabrication, thermoplastic polymers such as SIS, SEBS, and TPU are dissolved in toluene to disperse LM droplets and filler particles uniformly, forming functional LM‐polymer or LM‐filler‐polymer composites.

Beyond its role in fabrication, toluene is critical in the disassembly and recycling of LM‐based composites. When used composites are mixed with toluene, the polymer matrix dissolves, releasing the embedded LM droplets. This process yields a polymer solution similar to the original mixture, allowing the recovered LM to be reused in new composite films. The LM droplets retain their morphology after recycling, and the resulting films maintain strong mechanical properties, including high stretchability up to 1000% strain. Although there may be a slight reduction in strain‐stiffening behavior, the recycled composites remain functionally durable (Figure 17C).^[^ ^40^ ^]^ Toluene also enables circuit reconfiguration by selectively dissolving regions of the polymer matrix. This allows conductive LM traces to be erased and new paths to be created without damaging the substrate or components. After treatment, the erased regions become electrically insulating, confirming that the original circuit is fully deactivated. New circuits can then be patterned on the recycled composite, and their electrical performance remains reliable under repeated operation (Figure 17D). The microstructure of the LM droplets also remains largely unchanged, retaining their spherical or ellipsoidal shapes even after being recycled twice. In summary, toluene supports a closed‐loop approach to LM‐based electronics by facilitating both material fabrication and end‐of‐life recovery. Its ability to dissolve thermoplastic matrices without affecting LM droplets allows for efficient separation, recovery, and reuse of materials. This process enables the development of recyclable, reconfigurable, and sustainable soft electronic systems while reducing electronic waste.

#### 7.2.4 Sodium Hydroxide (NaOH) in Liquid Metal Extraction and Recycling

Sodium hydroxide (NaOH) is widely used in the recycling of gallium‐based LM from electronic applications.^[^ ^175^, ^461^, ^462^, ^463^ ^]^ It plays a crucial role in enhancing LM recovery by increasing interfacial tension and effectively removing oxide layers.

##### Enhancing LM Recovery Through Interfacial Tension Control

**Figure 18:**
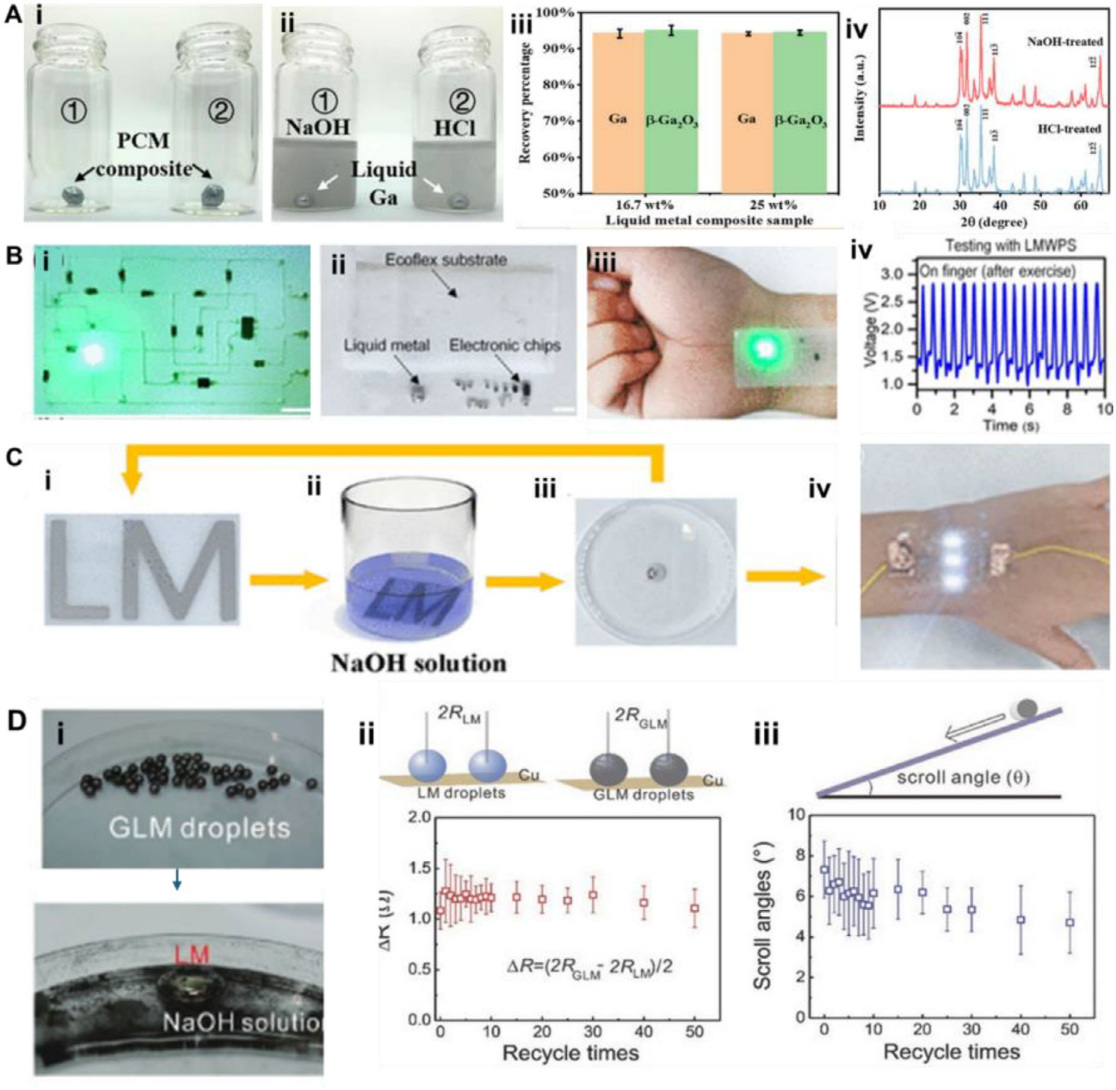
A) i) Waste (used) of the pristine PCM composites. ii) Recovering of LM droplets by putting them in NaOH and HCl solutions, iii) Recovery efficiency of Ga and Ga_2_O_3_ particles from PCM composites after HCl treatment. iv) XRD patterns of the recovered Ga_2_O_3_ particles. Reproduced with permission,^[^ ^175^ ^]^ Copyright 2022, Elsevier. B) i) LM‐based circuit, ii) immersed in 1 mol L^−1^ NaOH solution to recover the LM droplets and microchips, iii) the composite and circuit were reassembled, and the rebuilt system was tested, and the corresponding results were obtained. Reproduced with permission,^[^ ^462^ ^]^ Copyright 2021, ACS. C) The “LM” pattern printed using LM–PVA composite ink (i), followed by recycling and recovery of LM particles (ii), and reconstruction of an electronic tattoo from the extracted LM (iii). Reproduced with permission,^[^ ^126^ ^]^ Copyright 2021, ACS Publications. D) i) Recycling process of the as‐prepared GLM droplets by sequential rinsing with an aqueous NaOH solution. ii) Increase in resistance (Δ*R*) of the GLM droplets as a function of the number of recycling cycles, where *R*_LM_ and *R*_GLM_ represent the measured resistance of the LM and GLM droplets, respectively. iii) Change in scroll angles of the GLM droplets concerning the number of recycling cycles. Reproduced with permission,^[^ ^461^ ^]^ 2018, Wiley.

The addition of NaOH solution increases the interfacial tension of LM droplets,^[^ ^464^ ^]^ promoting their transformation into spherical shapes. This shape change facilitates droplet coalescence, enabling smaller LM droplets to merge into larger ones, thereby enhancing recovery efficiency.^[^ ^465^ ^]^ Additionally, sonication aids in the recovery process by removing oxide layers from Ga and breaking OH group interactions within the composite matrix.^[^ ^466^ ^]^ The LM droplets are treated with an aqueous NaOH solution, typically at a concentration of 10 wt%.^[^ ^461^ ^]^ Immersing e‐waste in a 1 m NaOH solution and subjecting it to sonication for 30 minutes leads to the dispersion of Ga_2_O_3_ particles in the solution, while Ga LM separates into distinct spheres due to its high surface tension (**Figure** **18**A,i,ii).^[^ ^175^ ^]^ Following vacuum filtration and drying, both Ga_2_O_3_ particles and Ga LM can be recovered with an efficiency of ≈95% (Figure 18A,iii). The chemical reactions between NaOH and gallium oxides, as well as NaOH and gallium, are as follows:

(3) $$Ga_{2}O_{3}+2\mathrm{NaOH}\to 2\mathrm{NaGa}O_{2}+H_{2}O$$

(4) $$2\mathrm{Ga}+2\mathrm{NaOH}+2H_{2}O\to 3H_{2}+2\mathrm{NaGa}O_{2}$$

(5) $$2\mathrm{Ga}+6\mathrm{NaOH}+xH_{2}O\to 3H_{2}+2Na_{3}\mathrm{Ga}O_{3}+xH_{2}O$$

(6) $$2\mathrm{Ga}+2\mathrm{NaOH}+6H_{2}O\to 3H_{2}+2\mathrm{NaGa}{\left(\mathrm{OH}\right)}_{4}$$

The recovered Ga_2_O_3_ particles retain their crystalline structure (Figure 18A,iv), ensuring their stability and potential for reuse. The observed mass loss during recovery is primarily attributed to physical processes occurring during treatment.

##### Efficient Oxide Layer Removal

NaOH is preferred over hydrochloric acid (HCl) for removing the oxide layer from LM surfaces due to its higher dissolution rate and reduced risk of dissolving gallium‐based LMs.^[^ ^175^, ^463^, ^465^, ^467^ ^]^ In one study, a stretchable multilayer LMWPS was utilized for personal health monitoring applications (Figure 18C,i).^[^ ^462^ ^]^ When immersed in a 1 mol L^−1^ NaOH solution, the oxidized LM reacted with the solution and reverted to its liquid state. With slight pressure, the LM could be easily expelled from the substrate and collected, as illustrated in Figure 18C(ii). Owing to the large difference in Young's modulus between the soft Ecoflex substrate and the rigid chip components, the chips were easily detached using tweezers. The recovered materials were reused to reconstruct the pulse sensor, which was reattached to human skin (Figure 18C,iii), and the corresponding sensing performance is shown in Figure 18C(iv). In another study, the pattern containing the word “LM”, printed with PVA‐LM ink, was immersed in a 1 M NaOH solution (Figure 18C,i,ii).^[^ ^126^ ^]^ After stirring for a period of time, the LM was recovered in an agglomerated form, retaining its original fluidity and electrical conductivity. The recycled LM was then used to regenerate the PVA‐LM ink, which was reprinted onto a PU film and applied as an electronic tattoo. As illustrated in Figure 18C(iii,iv), the tattoo adhered well to the wrist, and the circuit remained conductive during wrist movement, with the LED brightness unaffected by bending. These multifunctional characteristics demonstrate the promising potential of LM for flexible and wearable electronic skin applications.

In a similar approach, graphene‐coated LM (GLM) droplets were immersed in a NaOH solution, enabling the recovery of the LM droplets (Figure 18D,i).^[^ ^461^ ^]^ The change in resistance (ΔR) of the GLM droplets across multiple recycling cycles is shown in Figure 18D(ii), where *R*_LM_ and *R*_GLM_ denote the measured resistances of pristine LM and recycled GLM droplets, respectively. The corresponding variation in scroll angle during successive recycling steps is presented in Figure 18D‐iii. Notably, while ΔR remains nearly constant throughout the recycling process, the scroll angle decreases from 7.3 ± 1.4° to 4.7 ± 1.5° after 50 cycles. These findings indicate that the recycling strategy not only preserves the functional performance of GLM droplets but also offers a cost‐effective and scalable route for reuse. Moreover, this straightforward and robust method is likely applicable to a broad range of LM‐based electronic systems. These results highlight NaOH's efficiency in LM recycling, offering a sustainable approach to reclaiming valuable materials from electronic waste while minimizing environmental impact.

#### 7.2.5 Hydrochloric Acid (HCl) in Liquid Metal Recycling

HCl plays a role in LM recycling by removing the oxide layer from LM droplets.^[^ ^203^, ^468^, ^469^, ^470^ ^]^ The chemical equations for the reactions between HCl and gallium oxides, as well as HCl and gallium, are as follows:

(7) $$Ga_{2}O_{3}+6\mathrm{HCl}\to 2\mathrm{GaC}l_{3}+3H_{2}O$$

(8) $$2\mathrm{Ga}+6\mathrm{HCl}\to 2\mathrm{GaC}l_{3}+3H_{2}$$

**Figure 19:**
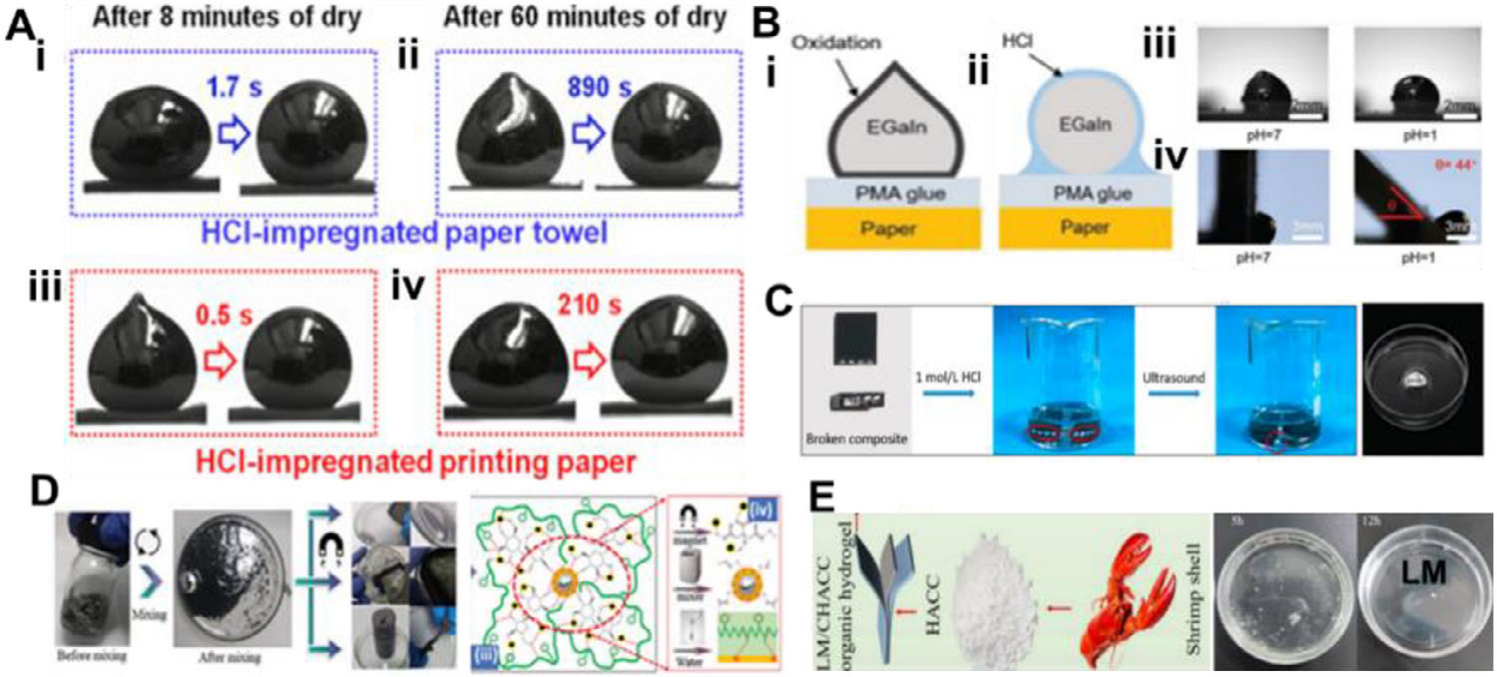
A) Optical images of liquid metal droplet shape change after 8 and 60 min on HCl‐impregnated i, ii) paper towel and iii, iv) printing paper. Time (s) indicates the duration for shape change via chemical reaction. Reproduced with permission,^[^ ^471^ ^]^ Copyright 2015, Elsevier. B) i) Schematic of oxidized LM on a substrate. ii) Oxide layer removal using HCl solution. iii) Contact angle images of LM droplets on PMA glue in neutral and acidic conditions. iv) Tilt‐angle photos showing EGaIn behavior on PMA glue with different HCl concentrations. Reproduced with permission,^[^ ^469^ ^]^ Copyright 2018, Wiley. C) Recycling of LM–CIPs/PDMS composite via HCl treatment and ultrasonication, yielding ≈98% recovery of reusable liquid metal. Reproduced with permission,^[^ ^203^ ^]^ Copyright 2022, ACS Publications. D) Ni particles were extracted using a magnet, LMs were removed by mechanical force, and the SIS polymer was separated with the aid of mechanical force and water washing. Reproduced with permission,^[^ ^19^ ^]^ Copyright 2023, Wiley. E) Fabrication of the LM/CHACC hydrogel composite and recovery of liquid metals using HCl. Reproduced with permission,^[^ ^473^ ^]^ Copyright 2022, Wiley.

However, compared to NaOH, HCl dissolves Ga‐based LMs at a slower rate and requires higher concentrations for effective oxide removal.^[^ ^175^, ^463^ ^]^ Due to these limitations, HCl is generally considered less efficient than NaOH for LM recycling. Despite this, HCl has demonstrated unique advantages in other applications involving Ga‐based LMs. One study introduced a simple HCl impregnation method to significantly enhance the lyophobicity of paper against Ga‐based LM. This approach enabled the fabrication of cost‐effective and efficient microfluidic channels for Galinstan. Given its affordability, ease of fabrication, and flexibility, the paper has gained attention as a microfluidic platform for various applications. The study found that HCl impregnation effectively modified certain types of paper, making them highly lyophobic to Ga‐based LMs, with stability lasting over 30 days. To validate its effectiveness, the researchers successfully manipulated a Galinstan droplet along microfluidic channels formed on the treated paper, demonstrating its potential for LM‐based microfluidic systems (**Figure** **19**A).^[^ ^471^ ^]^ Another study demonstrated HCl's effectiveness in removing EGaIn adhered to PMA glue, reducing fabrication costs and environmental impact. The oxide layer on EGaIn dissolves in acidic or alkaline solutions, breaking its chemical interaction with PMA glue (Figure 19B,i,ii). The reaction governing Ga_2_O_3_ dissolution in HCl is:

(9) $$Ga_{2}O^{3}+6H^{+}\to 2Ga^{3+}+3H_{2}O$$

Contact angle measurements showed that decreasing HCl concentration increased EGaIn's contact angle, reducing adhesion to PMA glue (Figure 19B). Lift‐up and obliquity tests further evaluated EGaIn's adhesive force under varying HCl concentrations (Figure 19B,iii,iv). Notably, a pH = 1 HCl solution effectively removed EGaIn from paper substrates, significantly lowering the cost of LM‐based paper electronics. After treatment, only ≈0.2 mg of EGaIn remained, compared to an initial printed weight of ≈0.1 g.^[^ ^469^ ^]^ EGaIn wire on paper and PMA glue can be effectively recycled using HCl, which dissolves the surface oxide layer and breaks adhesion. This enables EGaIn recovery, reducing material waste and the cost of LM‐based paper electronics. Similar to NaOH, HCl increases LM surface tension in an acidic environment,^[^ ^464^, ^468^ ^]^ facilitating its detachment from elastomer matrices.

The separation process can be further enhanced by stirring and ultrasonic treatment, which helps dissolve the oxide skin around LM droplets. Once freed, the LM droplets coalesce into larger, reusable droplets. In one study, an LM‐carbonyl iron particle (CIP)‐PDMS composite was immersed in a 1 mol L^−1^ HCl solution (Figure 19C). The treatment increased the LM surface tension, aiding its separation from the elastomer. Stirring and ultrasound further improved oxide skin dissolution, enabling the LM droplets to merge into macroscopic, reusable forms, achieving a recycling efficiency of nearly 98%.^[^ ^203^ ^]^ For LM‐nanoclay composites, the hydrophilic nature of nanoclay and its island‐like distribution within LM facilitate recovery using a 2 m HCl solution. Upon HCl addition, bubbles form, and fresh LM droplets accumulate at the container's bottom. This process occurs in two steps: i) Dissolution of oxide films surrounding nanoclay clusters, dispersing them into the aqueous solution. ii) Dissolution of oxide films maintaining the LM shape, allowing the LM within conductive nanoclay to merge into the bulk LM. This approach enables effective recovery of conductive LM from nanoclay structures.^[^ ^470^ ^]^ Moreover, EGaIn was extracted from the composite wire using a mixture of toluene and HCl solution.^[^ ^472^ ^]^

A separate study explored acid‐based methods for recovering LM particles from LM‐Ni‐SIS composite e‐waste, assuming that acids would dissolve the oxide layer and coalesce LM particles (Figure 19D).^[^ ^19^ ^]^ However, HCl unexpectedly increased adhesion between SIS and other components, hindering separation. The Ni particles are also trapped in the SIS matrix, making magnetic separation ineffective; alternative approaches are discussed in Section 8.1.

In another work, an LM/CHACC hydrogel composite with exceptional stretchability, rapid responsiveness, recyclability, and low‐temperature stability (GF = 8.29 at −20 °C) is fabricated by evaporating an ethanol‐water co‐solvent (Figure 19E). LM acts as a conductive filler, recyclable under acidic conditions.^[^ ^473^ ^]^ The hydrogel consists of CHACC layers chemically cross‐linked with epichlorohydrin, preventing fusion upon heating. When treated with acid, the hydrogel dissolves, enabling LM recovery. Adding 2M HCl to the waste LM/CHACC hydrogel generates bubbles and fresh metal droplets as the 3D network breaks down. This degradation occurs through the dissociation of Ga^3^⁺–OH complexes and the corrosion of the gallium–indium oxide shell, releasing fresh LM. The byproducts are environmentally safe, with Ga^3^⁺ ions from Ga oxide being applicable in FDA‐approved pharmaceuticals.^[^ ^474^ ^]^

#### 7.2.6 Dimethylformamide (DMF) in LM Recovery

**Figure 20:**
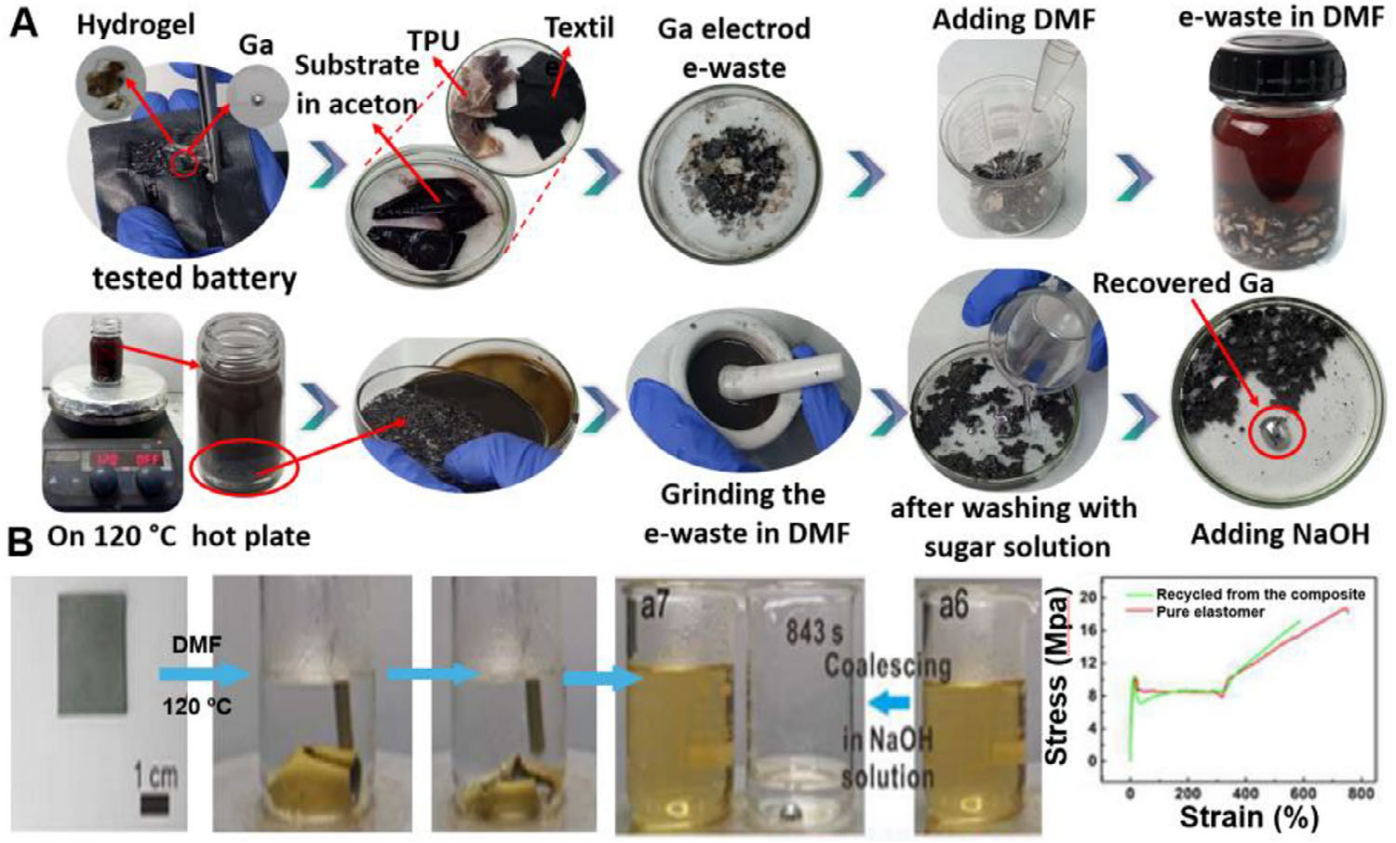
A) Schematic representation of the recycling process of the damaged LM‐based battery with the assistance of DMF. Reproduced with permission,^[^ ^20^ ^]^ Copyright 2024, Wiley. B) Recycling process of the LM–PU composite using DMF, followed by LM recovery with NaOH, accompanied by representative stress‐strain curves of the original and reprocessed composite. Reproduced with permission,^[^ ^121^ ^]^ Copyright 2022, MDPI.

Dimethylformamide (DMF) serves as a solvent in LM recovery through solvent‐based extraction methods. In this process, the LM‐elastomer composite is dissolved in DMF at 120 °C, allowing the separation of LM droplets from the elastomer matrix (**Figure** **20**A).^[^ ^20^ ^]^ Centrifugation is then used to isolate the LM droplets from the solvent, enabling their recovery for subsequent recycling. After centrifugation, the effluent containing micro/nanodroplets of LM is treated with a base solution, such as 0.6 M NaOH, which disrupts the interactions between the LM droplets and the polymer matrix. This treatment promotes the aggregation of LM droplets into macroscopic forms. The recovered LM droplets are then further pulverized to enhance their coalescence, resulting in larger droplets suitable for reuse. This recycling method has shown a high recovery efficiency of ≈98%,^[^ ^20^ ^]^ and 88% ± 3%,^[^ ^475^ ^]^ with the composition of the recycled LM closely resembling the raw material, consisting of 68.5% Ga, 21.5% In, and 10% Sn.

In a similar approach, heating the Ga–PU composite in DMF at 120 °C enables the collection of Ga droplets. A subsequent base treatment removes the oxide layer from the Ga microdroplets, allowing them to coalesce into larger LM droplets. This process achieves a high recycling efficiency of 96.7%, attributed to the dissociation of DA bonds in the composite, which facilitates the complete dissolution of the polymer matrix at high temperatures without the need for catalysts.^[^ ^121^ ^]^ The recovered PU elastomer, obtained after DMF removal and heating, retains mechanical properties similar to the pristine elastomer. In short, DMF alone breaks down the constituents of the composite, and subsequent treatment with NaOH enables the recovery of LM droplets. However, due to the high toxicity of DMF, a lower‐toxicity solvent mixture—comprising methyl acetate, cyclohexane, and 4‐chlorobenzotrifluoride (referred to as MCCCBF)^[^ ^19^ ^]^—was employed. The composite waste was immersed in MCCCBF for 24 hours and mechanically mixed at 2000 RPM for 3 min, resulting in the complete dissolution of all components. Ga LM particles were then recovered by introducing a sugar solution to the mixture. Although this method is more convenient than DMF‐based extraction, it is not entirely environmentally benign and may still pose certain side effects. **Table** **5** summarizes the advantages, challenges, and application domains of NaOH, HCl, and DMF in LM extraction and recycling.

**Table 5:** Comparison of NaOH, HCl, and DMF for LM extraction and recycling: advantages, challenges, and applications.^[^ ^10^, ^19^, ^20^, ^121^, ^126^, ^175^, ^203^, ^314^, ^463^, ^464^, ^468^, ^469^, ^470^, ^471^, ^475^, ^476^, ^477^, ^478^ ^]^

Aspect	Sodium hydroxide (NaOH)	Hydrochloric acid (HCl)	Dimethylformamide (DMF)
Advantages	The higher dissolution rate of the oxide layer	Widely available and relatively inexpensive	Efficient solvent for dissolving elastomer matrix
	Lower risk of dissolving gallium‐based liquid metals	Facilitates detachment of LM from elastomer matrix	Enables isolation of LM droplets from the solvent
	Increases interfacial tension, facilitating droplet coalescence	A high recovery rate of approximately 98%	Allows for the recovery of macroscopic LM droplets
Discovery of LM Extraction	Historically used in various industrial processes due to its reactivity	Well‐established chemical with known properties and applications	Widely employed as a solvent in various fields
	Specific role in LM extraction and recycling demonstrated in recent studies	Commonly used in LM extraction and recycling processes	Recognized as a solvent for LM extraction in recent research
Challenges	Requires careful handling due to its caustic nature	Slower dissolution rate of oxide layer compared to NaOH	The heating process may require specialized equipment
	Disposal of chemical waste generated during the recycling process	Higher concentrations may be needed for effective oxide layer removal	The extraction process may require optimization for different LM
	Potential safety hazards if mishandled or improperly used	Adverse effect on gallium‐based liquid metals	Requires additional treatment to recover macroscopic LM
Recycling Efficiency	Achieves a high retrieval rate (e.g., about 96% of LM)	A high recovery rate of approximately 98%	Demonstrates high recycling efficiency of approximately 88%
Environmental Impact	Minimizes environmental impact through efficient oxide layer removal	Potentially generates chemical waste during the recycling process	Solvent‐based extraction may pose environmental concerns
	Contributes to resource conservation by facilitating LM recovery	An acidic environment may require careful handling and disposal	The recycling process may require careful solvent management
Applications	Widely used in LM recycling processes for various electronic applications	Commonly employed in industrial settings for chemical processes	Utilized in solvent‐based extraction methods for LM recovery
	Plays a crucial role in enhancing the sustainability of electronic devices	Facilitates efficient LM reclamation from elastomer composites	Enables the recycling of LM embedded in elastomer matrices
Cost‐effectiveness	The cost of procurement and disposal may be relatively higher due to the caustic nature	Lower cost of procurement compared to NaOH	Cost of solvent and disposal may vary depending on application and scale
	Potential cost savings through recycling compared to sourcing new materials	Efficiency in usage may offset higher concentrations needed for LM recovery	The cost of heating equipment and solvent may influence overall cost‐effectiveness

## 8 Sustainable Methods for LM (Liquid Metal) Extraction

### 8.1 Biocompatible Aqueous Recycling Approaches

As mentioned in Section 6.2.4, HCl can sometimes hinder rather than enable LM recovery. For example, in LM–Ni–SIS composites, HCl unexpectedly increased adhesion between SIS and the other components, preventing effective LM separation, while Ni particles remained trapped in the SIS matrix, making magnetic recovery ineffective. These limitations highlight the need for safer and more efficient alternatives to strong mineral acids. To address this, two biocompatible aqueous approaches have been reported:^[^ ^19^ ^]^

- Epsom salt (MgSO_4_) solution: Sulfate anions interact with the LM oxide layer, weakening interfacial adhesion and promoting coalescence of LM droplets and Ni particle release.
- Sugar solution: A simple, low‐cost, and eco‐friendly process that achieves full LM droplet separation without the use of harsh chemicals.

In both cases, planetary mixing of SIS–Ni composites with the aqueous solution was followed by magnetic separation and water washing, achieving LM recovery efficiencies of ≈99.5%. Compared with aggressive chemical routes such as NaOH, DMF, or HNO_3_–HCl mixtures,^[^ ^10^, ^462^, ^475^ ^]^ these aqueous strategies offer a safer, low‐toxicity, and environmentally sustainable pathway for recycling LM composites.

Beyond these simple aqueous treatments, organic acids provide another promising class of mild lixiviants. Oxalic acid is particularly effective in dissolving gallium oxides and forming gallium oxalate complexes, while simultaneously precipitating interfering ions such as Fe^2^⁺ as ferrous oxalate. Under optimized conditions (e.g., ≈0.7 m oxalic acid, controlled pH, and temperature),^[^ ^479^ ^]^ gallium recovery efficiencies above 90% can be achieved within one hour. Although greener than mineral acids, oxalic acid is not fully biocompatible: its calcium‐chelating ability makes it potentially harmful at high concentrations, necessitating careful handling.^[^ ^480^ ^]^ In contrast, citric acid is biodegradable, food‐grade, and widely regarded as biocompatible. Its multiple carboxyl groups enable effective complexation with gallium, though the leaching kinetics are generally slower than oxalic acid.^[^ ^19^ ^]^ This makes citric acid particularly appealing in contexts where environmental friendliness and low toxicity outweigh the need for rapid leaching. Overall, approaches based on Epsom salt, sugar solutions, oxalic acid, and citric acid demonstrate that LM recycling can be achieved through safe, efficient, and environmentally conscious methods. Each offers distinct advantages—aqueous salts and sugars enable near‐complete LM recovery without chemical hazards, while organic acids provide selective dissolution of gallium oxides. By tailoring these strategies to specific LM systems and process goals, high recovery efficiency can be achieved while adhering to the principles of sustainability and the 4R framework for circular use of LM‐based materials.

### 8.2 Deep Eutectic Solvents (DESs) in LM Extraction

**Figure 21:**
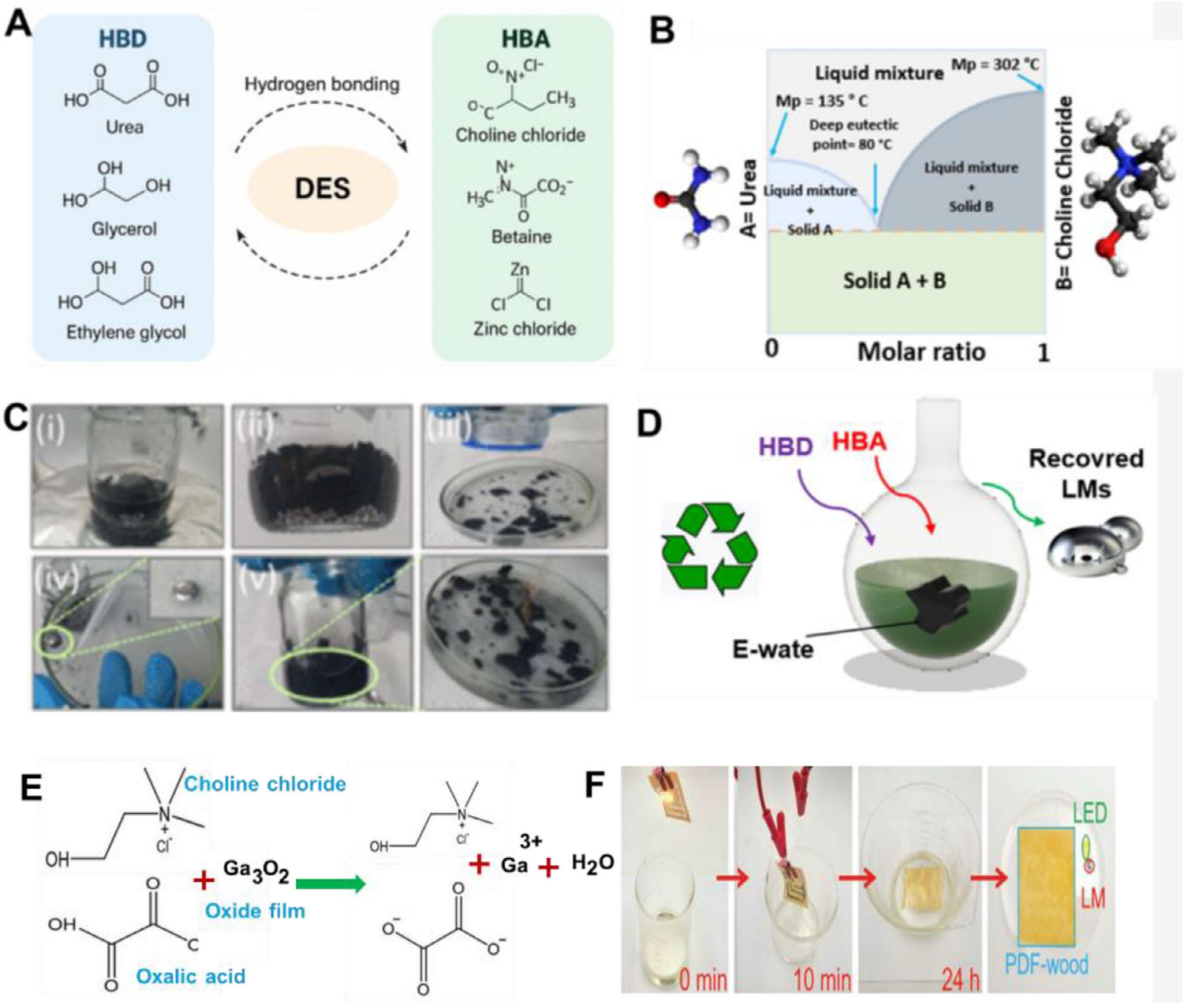
A) Examples of DESs, along with their hydrogen bond donors (HBDs) and hydrogen bond acceptors (HBAs). B) Schematic of DES preparation using oxalic acid and choline chloride (2:1 molar ratio). C) Recycling of Ga–CB–SIS composite ink e‐waste using the DES method. (B and C)Reproduced with permission,^[^ ^20^ ^]^ Copyright 2024, ACS Publications. D) Extraction of LM from LM‐Ag@Ga‐SIS Composite Using DESs. Reproduced with permission,^[^ ^21^ ^]^ Copyright 2024, ACS Publications. E) Chemical reactions responsible for the dissolution of the oxide film of LM via DES; and F) PDF‐wood was detached from the device, evidenced by LED shutdown upon exposing the LMWA conductor to DES. Reproduced with permission,^[^ ^487^ ^]^ Copyright 2022, Wiley.

DESs are environmentally friendly solvents known for their low toxicity and eco‐friendly nature.^[^ ^481^, ^482^ ^]^ They are created by combining two or more components, typically a quaternary ammonium salt (e.g., choline chloride) and a hydrogen bond donor (e.g., urea or glycerol) (**Figure** **21**A,B).^[^ ^483^, ^484^ ^]^ DESs are characterized by their low melting points, often below room temperature, and their ability to dissolve a wide range of organic and inorganic compounds, making them an attractive option for various applications, including LM extraction (Figure 21C,D).^[^ ^21^, ^485^ ^]^ When used to extract LM from matrices such as elastomer composites or electronic devices, the mechanisms of extraction can vary depending on the specific DES composition and the properties of the LM.^[^ ^21^, ^486^ ^]^ Several potential mechanisms may contribute to the extraction process:

1. Solubilization: Due to their unique composition and polarity, DES can interact with both the LM and the matrix material. This interaction may lead to the solubilization of the LM within the DES phase, effectively separating it from the matrix.
2. Surface oxide removal: Some DES formulations can dissolve or react with the oxide layer typically found on the surface of liquid metals, such as Ga‐based alloys. By removing this oxide layer, DES can facilitate the release of the LM from the matrix.
3. Complexation: Components of DES may form coordination compounds or complexes with certain elements in the LM or matrix material. This complexation could alter the solubility or chemical properties of the LM, aiding its extraction.
4. Liquid–liquid extraction: DES may act as a medium for liquid‐liquid extraction, where the LM preferentially partitions into the DES phase, separating it from the solid matrix. This mechanism is based on differences in solubility and affinity between the LM, the DES, and the matrix material.

These mechanisms, whether occurring individually or in combination, could make DES an effective solvent system for extracting LMs from various matrices. The specific DES formulation and experimental conditions would need to be optimized for efficient and selective LM extraction. Further research is needed to explore the potential of DES as a sustainable and versatile solvent for LM recovery and recycling.

The recovery of liquid metals (LMs) using deep eutectic solvents (DESs) relies on several complementary mechanisms. DESs provide a tunable hydrogen‐bonding network and strong complexation capacity that can i) disrupt the Ga‐oxide/oxyhydroxide skin stabilizing LM droplets, ii) weakly solvate or partition Ga‐alloy species, and iii) promote droplet coalescence under mild, low‐volatility conditions. Together, these effects enable selective LM liberation from soft matrices without harsh acids or bases.^[^ ^20^, ^83^, ^485^, ^488^, ^489^ ^]^ Practically, acidic DESs such as choline chloride: oxalic acid (1:2, HBA: HBD) are highly effective for oxide removal and complexation, whereas neutral systems like choline chloride: urea (1:2) or choline chloride: glycerol are gentler when polymer compatibility is critical. Typical conditions involve 40–80 °C with agitation or ultrasound to offset viscosity and accelerate mass transfer, after which LM droplets detach, coalesce, and sediment. The LM is then separated, and the DES phase can be rinsed, filtered, and reused with modest replenishment.^[^ ^485^, ^490^, ^491^ ^]^ Despite these advantages, DESs can be viscous and hygroscopic; since water content alters speciation and kinetics, temperature control or slight dilution is often applied to manage viscosity and water uptake, and multi‐cycle reuse must be validated.^[^ ^492^, ^493^ ^]^ This has been validated in device‐level studies. In one example, gallium was recovered from printed Ga–CB–SIS circuits by immersing the e‐waste in a choline chloride: oxalic acid DES (1:2 molar ratio) heated to 80 °C. After homogenization, the printed scraps were stirred in the warm DES; most Ga droplets detached and coalesced under magnetic stirring, with the remainder released by brief mechanical mixing. The process achieved ≈98% recovery efficiency. Mechanistically, the strongly acidic DES (pH ≈0.5) disrupts the Ga‐oxide skin and interfacial bonding to the elastomer, increases the apparent surface tension at the LM/matrix interface, and complexes surface species via the oxalic acid–choline network, enabling droplet coalescence and separation under mild, low‐volatility conditions.^[^ ^20^ ^]^ Similarly, another study recovered LM from delignified‐wood circuits using the same acidic DES (ChCl: OxA, 1:2). The solvent was prepared by heating to ≈80 °C until clear, and immersion at room temperature sufficed to dissolve the Ga_2_O_3_ oxide skin, reduce LM–substrate adhesion, and trigger droplet detachment and coalescence. Droplets sedimented under gravity, with XPS (Ga 3d) confirming the reduction of Ga^3^⁺ consistent with oxide removal. Device‐level recycling was further demonstrated as LED circuits sequentially switched off during the process. After ≈24 h at room temperature, ≈96% LM recovery was achieved, with higher temperatures proportionally shortening recovery times. This approach preserved the wood substrate and components, avoided strong mineral acids or alkali, and was compatible with solvent reuse, highlighting a mild, low‐volatility recycling route for Ga‐based LM electronics (Figures 21 E and F).^[^ ^487^ ^]^

Beyond LM devices, DESs have been widely applied for the recovery of LM‐relevant metals, underscoring their transferability to Ga‐In‐Sn alloys. Choline chloride–organic acid systems (e.g., ChCl: oxalic, glycolic, or other carboxylates) leach and separate indium and tin from oxide matrices and zinc flue dust under mild conditions—chemistries directly applicable to Ga‐oxide skins and LM/polymer interfaces.^[^ ^494^, ^495^ ^]^ Foundational studies have also shown broad oxide dissolution in ChCl‐based DESs,^[^ ^489^ ^]^ while recent thermophysical datasets provide practical operating windows for ChCl–organic acid systems (40–80 °C viscosity curves).^[^ ^490^ ^]^ Reviews on multi‐cycle regeneration and solvent quality control further outline strategies for scaling LM recycling with DESs.^[^ ^496^, ^497^ ^]^ Compared to harsh NaOH or HCl routes, acidic DESs such as ChCl: OxA (1:2) operate under lower volatility and are less aggressive toward elastomers and adhesives. These features make them attractive when substrate preservation and solvent reuse are priorities, highlighting their promise as a sustainable platform for LM recovery and recycling.^[^ ^490^, ^491^ ^]^

### 8.3 Ionic Liquids (ILs) in LM Extraction

ILs are a class of organic salts that are liquid at or near room temperature. They are composed entirely of ions and typically consist of large organic cations paired with small inorganic or organic anions.^[^ ^498^, ^499^, ^500^, ^501^ ^]^ Due to their unique properties, such as low volatility, non‐flammability, and high thermal stability, ILs have garnered significant interest as green solvents in various chemical processes.^[^ ^502^, ^503^, ^504^ ^]^ Furthermore, ILs are often considered green solvents due to their biodegradability, low toxicity, and potential for recycling. They can be synthesized from renewable resources and designed to be recyclable, minimizing environmental impact and resource consumption.^[^ ^505^ ^]^ Additionally, ILs can enable more sustainable chemical processes by reducing energy requirements and waste generation.^[^ ^506^, ^507^ ^]^

In the context of LM extraction, ILs offer several advantages. Firstly, their tunable physicochemical properties allow for the design of ILs with high solubility for specific metals, including LMs like Ga, and Ga‐based alloys. This selectivity enables efficient extraction while minimizing the need for harsh conditions or toxic reagents.^[^ ^508^, ^509^ ^]^ Additionally, ILs can form stable complexes with metal ions,^[^ ^510^ ^]^ facilitating their separation from other components in a mixture. The mechanisms of LM extraction using ILs can vary depending on the specific IL and metal involved. However, some common mechanisms include coordination complexation, where ILs form coordination bonds with metal ions, and ion exchange, where metal ions are exchanged between the IL and the metal‐containing phase.^[^ ^511^, ^512^ ^]^ In both cases, the goal is to selectively extract the desired metal ions into the IL phase for subsequent separation and recovery. For instance, selectivity of Ga and In against other elements like Al, Fe, etc., depends on IL family (phosphonium vs. quaternary ammonium), acidity, and chloride activity; extraction generally increases with [Cl^−^], and counter‐transport of H⁺/Cl^−^ rationalizes equilibrium trends^[^ ^509^ ^]^ IL phases can also be immobilized as supported liquid membranes or polymer inclusion membranes to reduce solvent inventory while enabling continuous Ga transport from chloride feeds.^[^ ^513^ ^]^ Use an organic‐to‐aqueous phase (O/A) ratio of ≈1:1 at RT–40 °C, with vigorous but non‐emulsifying agitation. Mild heating lowers viscosity and aids separation; NaCl and diluent choice further control emulsions and phase disengagement. ILs are recyclable with negligible vapor pressure, but selection should favor low‐toxicity families and demonstrate multi‐cycle reuse.^[^ ^509^ ^]^

Studies show how ionic liquid (IL) design maps onto gallium recovery mechanisms. Kelex 100 in triazolium ILs extracts Ga from Bayer liquors either directly with hydrophobic [Tf_2_N]^−^ ILs or via thermomorphic aqueous biphasic systems after carbonation, offering faster kinetics and cleaner separations than kerosene.^[^ ^508^ ^]^ Hydrophobic tribromide ILs ([P_44410_][Br_3_]) leach GaAs/InAs under mild conditions while suppressing arsine, retaining Ga as [GaBr_4_]^−^, and enabling sequential aqueous separation of As, Ga (≈96%), and In with IL regeneration.^[^ ^514^ ^]^ MTOA iodide serves as a dual‐purpose IL, reducing Fe^3^⁺ to Fe^2^⁺ in situ and selectively extracting Ga(III) as [MTOA][GaCl_4_]^−^, with easy stripping and regeneration.^[^ ^515^ ^]^ Cyphos IL‐104 provides fast Ga(III) extraction from HCl with quantitative recovery from photodiodes and red mud.^[^ ^516^ ^]^ Task‐specific Aliquat‐336 ILs paired with organophosphorus acids favor In(III) over Ga(III) at weak/moderate HCl, enabling counter‐current separations.^[^ ^517^ ^]^ Collectively, these examples illustrate ILs as both designer extractants for [GaCl_4_]^−^‐type species and reactive media for feed pre‐conditioning. Unlike DESs, which excel in mild oxide removal, ILs offer superior selectivity and closed‐loop operation, making them complementary in LM recovery flowsheets.^[^ ^509^, ^516^ ^]^

Overall, the use of ILs for LM extraction represents a promising approach with significant potential for green and sustainable chemistry practices. By leveraging the unique properties of ILs, researchers can develop efficient and environmentally friendly processes for extracting and recovering valuable metals from various sources. **Table** **6** provides a comparative overview of DES and ILs for LM extraction, considering various aspects such as their definition, green solvent properties, selectivity, mechanisms of extraction, efficiency, applications, and environmental impact. Both DES and ILs offer unique advantages for LM extraction, with DES being characterized by its eutectic mixture properties and ILs by their ionic nature. Depending on the specific requirements of a given extraction process, either DES or ILs may be preferred due to their distinct properties and capabilities.

**Table 6:** Comparison of DESs and ILs for LM extraction.

Aspect	Deep eutectic solvents (DES)	Ionic liquids (ILs)
Definition	A mixture of a hydrogen bond acceptor (HBA) and a hydrogen bond donor (HBD) forms a eutectic mixture with a melting point lower than that of each individual component.	Organic salts are composed entirely of ions, typically large organic cations paired with small inorganic or organic anions, liquid at or near room temperature.
Green Solvent Properties	Biodegradable, low toxicity, derived from renewable resources.	Biodegradable, low toxicity, potentially recyclable.
Selectivity	Can be tailored for specific metal ions, including liquid metals.	Selective extraction capabilities based on IL composition and structure.
Mechanisms of Extraction	Hydrogen bonding interactions, complexation, ion exchange.	Coordination complexation, ion exchange, solvation.
Efficiency	Effective extraction under mild conditions, and high selectivity.	Efficient extraction with tunable properties, and high selectivity.
Applications	Widely used in various extraction processes, including metal recovery from ores and waste streams.	Applied in metal extraction, catalysis, and other chemical processes.
Environmental Impact	Generally considered green solvents, potentially recyclable.	Green solvent option can contribute to sustainable chemistry practices.

### 8.4 Extraction of LMs via the Gel–Sol Transition Method

The gel‐sol transition method is an innovative and efficient approach for extracting LMs from various solutions by leveraging the reversible transformation between gel and sol states.^[^ ^75^, ^131^ ^]^ This process begins with gel formation, where a sol‐gel transition is induced through the hydrolysis and condensation of precursors such as trialkoxysilanes. This results in a three‐dimensional gel network capable of encapsulating metal ions. Once the gel is formed, it selectively captures metal ions from the surrounding solution. Certain materials, such as peptide copolymer gels, demonstrate a strong affinity for specific metal ions—for example, gallium ions (Ga^3+^) and gold ions (Au^3^⁺)—due to the presence of amino functional groups at the polymer chain ends.^[^ ^518^, ^519^ ^]^ The captured metal ions remain trapped within the gel network until external conditions are modified. To recover the metal ions, the gel transitions back to a sol state in response to changes in environmental factors such as temperature, pH, or the addition of specific reagents. This transition releases the encapsulated metal ions, enabling their efficient extraction and purification. The gel‐sol transition method offers several advantages over conventional metal extraction techniques. Its high selectivity allows for precise targeting of specific metal ions, while its reversibility enables multiple reuse cycles, reducing material waste.^[^ ^520^ ^]^

**Figure 22:**
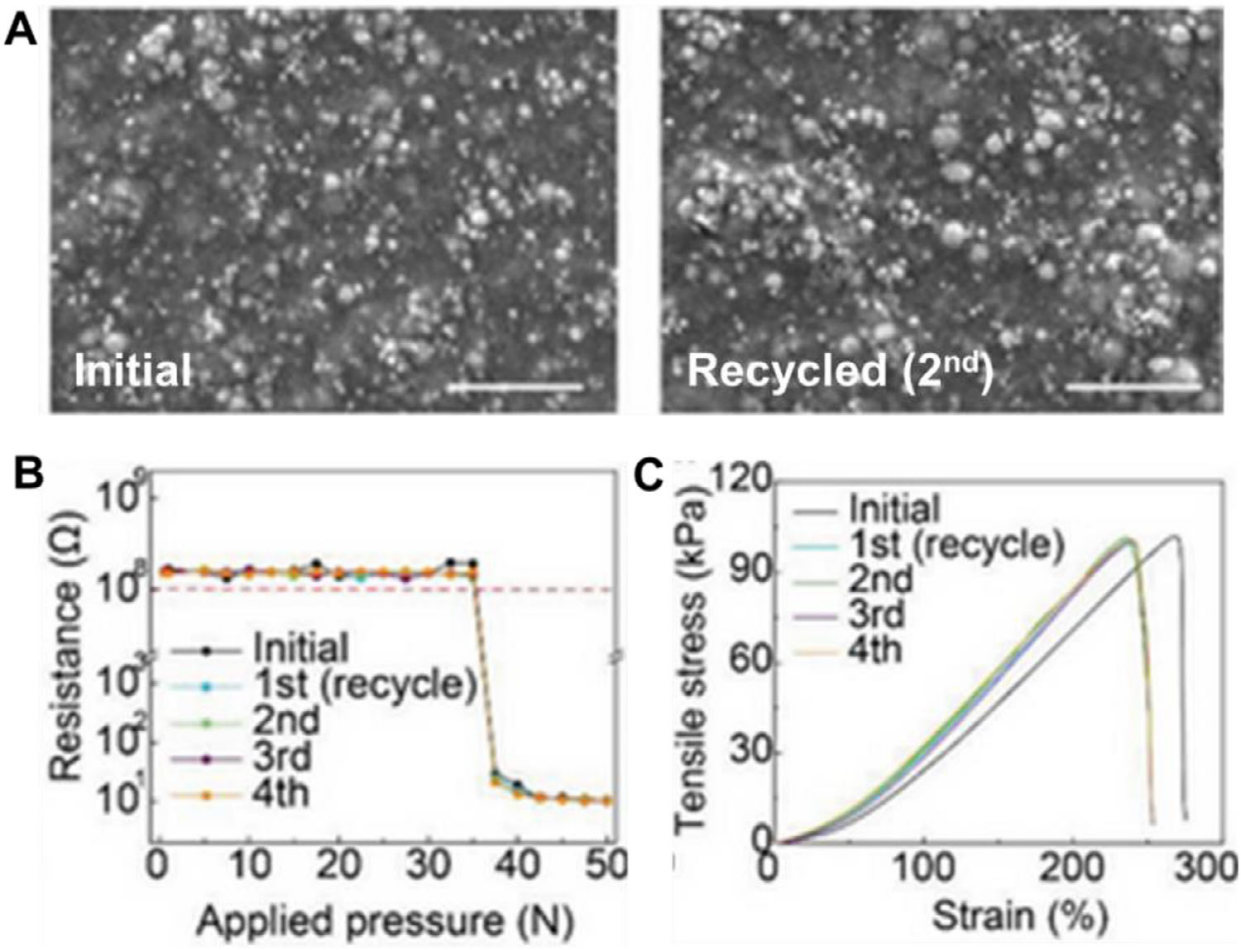
A) SEM images of LM‐gelatin ACFs in pristine (left) and second‐recycled (right) states. Scale bars: 5 µm. B) Resistance vs. applied pressure for LM‐gelatin ACFs before and after four recycling cycles. Red dotted line: max instrument limit. C) Tensile stress‐strain curve of LM‐gelatin ACFs before and after four recycling cycles. Reproduced with permission,^[^ ^521^ ^]^ Copyright 2022, Wiley.

Another feature of gel‐sol transition methods is that they can be eco‐friendly. They often employ biodegradable and non‐toxic materials, making it a sustainable alternative for wastewater treatment, industrial resource recovery, and metal extraction from electronic waste. In one work, the recyclability of LM‐gelatin anisotropic conductive films (ACFs) is enabled by the reversible sol‐gel transition of the gelatin hydrogel, in conjunction with the surface chemistry and interfacial tension of the LMs.^[^ ^521^ ^]^ SEM images of both as‐fabricated and recycled LM‐gelatin ACFs reveal minimal structural changes, highlighting the material's ability to be effectively recycled (**Figure** **22**A). The recycling process involves heating the LM‐gelatin ACFs to 90 °C, inducing the gel‐sol transition of the gelatin hydrogel, and converting the solid film into a liquid mixture. This mixture is then spin‐coated onto a newly prepared substrate, reforming the LM‐gelatin ACF with no significant alterations in morphology. The recycled ACFs maintain the original shapes and sizes of LM capsules, and their electrical conductivity remains unchanged at 49 S cm^−1^. The compression‐induced activation of anisotropic conduction in the recycled LM‐gelatin ACFs demonstrates performance consistency with the original ACF (Figure 22B). The activation force remains comparable, even after multiple recycling cycles (ACF thickness: 1 µm), and electrical conductivity is preserved across successive uses. The mechanical properties—including elastic modulus, tensile strength, and fracture stress—of the recycled ACFs remain similar to those of pristine LM‐gelatin ACFs, even after four recycling cycles (Figure 22C). In tensile stress‐strain tests, the LM‐gelatin ACFs measured 5.0 cm in length, 2.54 cm in width, and 0.1 cm in thickness, with only slight variations in elastic modulus attributed to minor water evaporation during the recycling process. This demonstrates that the gel‐sol transition method is not only effective for metal extraction but also plays a crucial role in the recyclability and sustainability of LM‐based materials.

## 9 LM‐Composite Printing and Microelectronics Interfacing

### 9.1 Challenges in Printing LM Composite Inks on Soft Substrates

Printing composite inks onto flexible and stretchable substrates presents several challenges that need to be addressed to achieve successful integration with electronic components. One of the main challenges is ensuring effective adhesion of the ink to various substrates to prevent smearing or distortion during the printing process.^[^ ^19^, ^75^ ^]^ Achieving precise deposition of the ink is critical for maintaining the functionality and reliability of the printed circuits. Factors such as ink viscosity, surface tension, substrate roughness, and the printing technique all influence the behavior of the ink. While optimizing ink properties and adjusting printing parameters are valuable, the key to practical circuit performance is preventing ink trace smearing.^[^ ^62^ ^]^ To overcome this challenge, it is essential to carefully select the proper materials for the formulation of a composite ink. LMs, which are often prone to smearing, require a focused approach to the choice of polymer binders and filler particles in the composite to mitigate such behavior. For instance, using SIS polymers as binders can enhance adhesion between the ingredients and provide reversible physicochemical properties, further improving the ink's adhesion and shelf‐healability.^[^ ^11^, ^20^, ^62^ ^]^ In cases where smearing still occurs, encapsulation can be employed as a protective measure. Encapsulation not only protects the circuit from environmental factors such as moisture, dust, and mechanical damage but also ensures the circuit remains flexible to accommodate the bending and stretching of the substrate.^[^ ^522^, ^523^ ^]^ The selection of encapsulation materials is pivotal, as they must offer a robust barrier while allowing for the required flexibility in flexible electronics.^[^ ^522^, ^523^ ^]^ Techniques such as coating or lamination are commonly used to achieve this protective barrier, contributing to the long‐term reliability and durability of printed circuits.^[^ ^524^ ^]^ Additionally, using degradable elastomers for encapsulation can lead to the development of degradable‐stretchable electronics.^[^ ^525^ ^]^ This approach not only protects the circuits but also facilitates full recycling, as microchips can be easily separated, and LMs can be recovered for reuse.^[^ ^453^, ^526^ ^]^ However, encapsulating with elastomers is a more complex process, often involving chemical interventions.

Solvent selection remains a critical and often underestimated challenge in the preparation of LM–polymer composites for soft electronics. The choice of solvent must carefully balance competing factors such as LM dispersion stability, polymer solubility, interfacial compatibility, and process safety. An ideal solvent must enable uniform dispersion of LM droplets or nanoparticles without inducing aggregation or surface oxidation, while simultaneously dissolving or swelling the polymer matrix to facilitate effective mixing and film formation. Additionally, the solvent must not chemically react with the LM, degrade the polymer, or compromise the functional properties of the final composite. Volatility, toxicity, and environmental impact further restrict solvent choices, especially when aiming for scalable and sustainable processing. Despite its importance, solvent selection is often determined empirically, and only limited studies have systematically explored solvent–LM–polymer interactions. This gap presents a significant barrier to reproducibility, performance optimization, and broader adoption of LM‐based composites in soft, recyclable electronic systems. Addressing this challenge requires a deeper investigation into solvent chemistry and LM surface behavior to guide rational solvent design and selection. Indeed, the preparation of LM–polymer composites, particularly those incorporating eutectic EGaIn, requires careful consideration of solvent choice to ensure uniform dispersion, compatibility between the LM and polymer phases, and appropriate rheological properties for processing. Solvents serve multiple purposes in this context: dissolving the polymer matrix, controlling the viscosity of the composite ink or slurry, and facilitating the stabilization and distribution of LM droplets within the polymer network.^[^ ^233^, ^527^, ^528^ ^]^

For elastomer‐based systems—widely used in flexible and stretchable electronics due to their high deformability and resilience—nonpolar or weakly polar solvents are typically employed. Polymers such as SIS and SEBS are readily dissolved in solvents like toluene,^[^ ^528^ ^]^ DMF/ chloroform, or cyclohexane, and moderately polar organic solvents like 1,2‐dichloroethane.^[^ ^529^ ^]^ Among these, toluene is one of the most commonly used due to its strong solvating ability for styrene block copolymers and its compatibility with LM droplets. These solvents promote good miscibility and aid in achieving uniform LM dispersion during solution blending or casting. In some cases, PDMS is used as the polymer matrix without any solvent;^[^ ^279^, ^530^ ^]^ LM is mixed directly with the PDMS prepolymer and curing agent. However, when viscosity reduction is needed, small amounts of some solvents, e.g., ethanol,^[^ ^531^ ^]^ chloroform/HCl,^[^ ^532^ ^]^ hexane, or toluene may be added to enhance the homogeneity and processability of the mixture.

In contrast, thermoplastic polymers such as PU and poly(methyl methacrylate) (PMMA) require more polar solvents due to their chemical structure. PU is commonly dissolved in solvents such as DMF,^[^ ^533^ ^]^ tetrahydrofuran (THF),^[^ ^223^ ^]^ or acetone. These polar solvents enable the fabrication of LM–PU composites through techniques like solution casting or spin‐coating, where the solvent facilitates not only polymer dissolution but also the emulsification and stabilization of LM droplets. Similarly, PMMA is typically processed with chloroform or acetone. These solvents offer good volatility and film‐forming properties, making them suitable for thin‐film applications. Nevertheless, care must be taken to avoid undesired side reactions or excessive oxidation of the LM during solvent evaporation or thermal curing.

Beyond polymer solubility, solvents are also employed to modify the surface characteristics of LMs. Due to the rapid formation of a native gallium oxide skin on the LM surface, pretreatment with mild acids (e.g., acetic acid) or alcohols (e.g., ethanol, isopropanol) is sometimes necessary to reduce the oxide layer, enhance wetting, and improve dispersion. In addition, the inclusion of surfactants in the solvent system can further stabilize LM droplets and prevent aggregation during processing. In summary, solvent selection is crucial for fabricating LM–polymer composites, influencing dispersion, interfacial interactions, morphology, and performance. Nonpolar solvents (e.g., toluene) suit rubbery copolymers, while polar ones (e.g., DMF, THF) fit thermoplastic matrices. The right solvent optimizes mechanical, thermal, and electrical properties.

### 9.2 Integration of Microchips with LM Composite over Soft Substrates

One of the fundamental challenges in engineering soft electronic systems is the integration of rigid microchip components. These systems must maintain electrical reliability and mechanical stability at the interface between the soft circuit and rigid components while undergoing dynamic mechanical deformation such as bending, stretching, or twisting. However, the intrinsic mechanical mismatch between stiff microchips and compliant substrates often leads to stress concentrations, interfacial delamination, and electrical failure.^[^ ^440^, ^534^, ^535^ ^]^ Traditional integration methods—such as wire bonding, flip‐chip bonding, or soldering—are generally designed for rigid circuit boards and prove inadequate for soft platforms.^[^ ^536^ ^]^ These techniques typically involve complex post‐processing, high temperatures, and three‐dimensional structures, all of which are incompatible with soft, stretchable/flexible substrates.^[^ ^537^, ^538^ ^]^ Notably, traditional wire bonding—widely used in IC packaging—forms an inherently 3D structure, making it extremely difficult to stack or integrate microfluidic components on top.^[^ ^539^ ^]^ Alternative methods like flip‐chip bonding can produce a flat device surface, but the active area is often encapsulated within the package, rendering it inaccessible for microfluidic interfacing.

One promising approach involves the use of PDMS substrates embedded with LM‐filled microchannels.^[^ ^540^ ^]^ These channels, filled with LM, form highly stretchable and conformal interconnects that electrically bridge the rigid microchip and soft circuitry. Because the LM remains fluid at room temperature, it accommodates strain without fracture, and the embedded architecture eliminates the need for post‐processing on the chip itself. This technique has shown excellent results in systems integrating CMOS chips with microfluidics, paving the way for compact and stretchable sensor platforms.

Another strategy utilizes porous elastomeric substrates fabricated by introducing pressurized steam into uncured PDMS.^[^ ^541^ ^]^ The resulting sponge‐like architecture can be metalized using electroplated nickel to form anchoring sites for commercial electronic components. Once anchored, LM‐based composite inks can be printed over the structure to form robust, flexible circuits. These systems have demonstrated excellent performance in wearable biosignal monitoring applications, such as ECG and EEG electrodes.^[^ ^541^ ^]^ The porosity of the substrate, combined with the compliance of the LM ink, enables greater stretchability and mechanical endurance, which has been validated through both fatigue testing and finite element analysis.

As an alternative, SIS is promising for interfacing with rigid components since it can be used as both the circuit substrate and dispersion medium for LM‐based conductive inks.^[^ ^20^, ^21^, ^261^ ^]^ SIS is a thermoplastic elastomer with reversible physical and chemical crosslinks, enabling phase‐shifting under solvent exposure. This feature allows for room‐temperature “soldering” of SMD components: when exposed to solvent vapor, the SIS softens and allows the microchip to sink slightly into the LM ink beneath it, forming strong mechanical and electrical contact. This solvent‐assisted bonding approach avoids thermal damage and supports the use of heat‐sensitive substrates. The SIS substrate not only provides elasticity and toughness but also supports circuit recycling through its solubility, aligning well with sustainability goals in electronics (reduce, reuse, recycle). Integrating microchips into SIS films enhances adhesion on all contact surfaces and distributes strain evenly across the interface, significantly improving durability under deformation.^[^ ^11^, ^20^ ^]^

In addition to substrate innovations, a major advancement in soft electronics is the use of anisotropic conductive films (ACFs) to enable vertical electrical interconnections between rigid microchips and soft substrates. Among these, LM–gelatin hybrid ACFs offer a compelling solution by embedding LM droplets within a gelatin matrix, providing both electrical conductivity and mechanical adhesion. A key advantage of this material is its self‐healing capability: under strain or damage, the gelatin allows LM droplets to reflow and restore conductivity, enhancing device reliability in dynamic environments. This property is crucial for applications like wearable devices, soft robotics, and electronic skin, where flexibility and durability are essential. A representative example is the integration of µ‐LEDs into stretchable displays.^[^ ^521^ ^]^ EGaIn traces are printed on a soft substrate, overlaid with a spin‐coated LM–gelatin ACF, and topped with transfer‐printed LEDs. This structure maintains stable performance under repeated deformation and outperforms traditional adhesives prone to delamination. In addition to flexibility and self‐healing, the LM–gelatin ACFs are recyclable, making them a robust and sustainable choice for next‐generation stretchable electronics.

Together, these techniques—spanning diverse material platforms and integration strategies—offer comprehensive solutions for bridging the mechanical and functional divide between rigid microchips and soft electronic systems. By harnessing the unique properties of liquid metals, such as high electrical conductivity, low modulus, and reconfigurability, and combining them with substrates that are porous, recyclable, or self‐healing, robust, high‐performance flexible electronics have been developed to endure real‐world mechanical stresses. These advancements are particularly impactful for applications requiring durability and resilience under deformation, including wearable health monitors, soft robotics, electronic skin, and stretchable displays. As the field evolves, the convergence of soft matter science, materials engineering, and microscale integration is redefining the interaction between rigid and soft components—bringing electronics closer to systems that conform to the human body, adapt to motion, and recover from mechanical damage.

## 10 Summary and Conclusion

As soft electronics technologies advance, there is a growing need to adopt materials that are sustainable, self‐healing, and recyclable composites. This shift is essential for reducing e‐waste and addressing environmental issues that pose significant risks to human health and the planet. Achieving this transition requires careful material selection before considering design or fabrication methods. While incorporating conductive fillers into stretchable polymer matrices has driven significant progress, these composites still struggle with maintaining stable electrical conductivity under strain, limiting their widespread application. To address this, eutectic LM alloys and LM composites have been introduced, offering self‐healing behavior, high stretchability, flexibility, and enhanced conductivity. In sum, gallium‐based LMs (EGaIn, Galinstan) pair metal‐level conductivity with large deformability and practical end‐of‐life routes; the most effective sustainability levers identified here are oxide‐dependent recovery chemistries, intermetallic‐resistant formulations, sinter‐free/low‐temperature fabrication that enables rework, and materials‐first design that anticipates disassembly and reuse.

However, challenges remain in fabricating electronic circuits due to the high surface tension of LM droplets. To overcome this, LM droplets and LM nanoparticles are incorporated into polymer matrices, significantly improving composite performance, though mechanical or thermal sintering is still required to achieve conductivity. In response to these challenges, biphasic LM‐X‐polymer composites have been developed, demonstrating exceptional electromechanical, thermal, and digital printing properties that can be produced at room temperature without the need for sintering. These innovations broaden the potential applications of polymer composites in various fields, including wearable devices, consumer systems, energy storage, EMI shielding, and thermal interface materials (TIMs). However, several challenges remain in advancing this technology. Furthermore, the selection of a stretchable, recyclable, and self‐healable substrate plays a crucial role in determining the overall performance and durability of the fabricated circuits. These substrate properties not only contribute to the mechanical resilience of the device under repeated deformation but also support sustainable design goals by enabling recyclability and extending the device's lifespan. In this context, compatibility between the substrate and the LM‐based composite ink is essential to ensure strong interfacial adhesion, uniform film formation, and reliable electrical performance. A mismatch in mechanical properties, chemical reactivity, or thermal stability between the substrate and the ink can lead to delamination, cracking, or loss of conductivity under operational conditions. Therefore, careful consideration of the substrate's physical and chemical characteristics is necessary to achieve optimal integration and functional performance in stretchable and environmentally conscious electronic systems. Key hurdles include developing cost‐effective and scalable production methods for digital‐printable composites, ensuring long‐term stability of mechanical and electrical properties under diverse environmental conditions, achieving uniform conductivity without sintering, integrating these composites efficiently with existing electronic systems, ensuring their environmental sustainability and economic viability for mass production, improving performance in extreme environments (such as high temperatures or exposure to chemicals), and optimizing composite structures to balance performance, cost, and fabrication ease. Addressing these challenges offers exciting opportunities for future research and development in advanced composite materials. Looking ahead to scale, a practical pathway is staged: pilot lines that prove solvent‐lean, oxide‐aware LM recovery and device reliability with reclaimed LM; early deployment with OEM take‐back and automated printing/inspection to cut scrap; and sector‐wide scale‐up with shared materials/recovery specifications and serialized end‐of‐life tracking—benchmarked by LM recovery yield, solvent recapture rate, energy per mass recovered, and stability across reuse cycles.

An essential yet challenging aspect of 4R (recyclable, repairable, renewable, resilient) soft electronics is the reclamation of LMs for reuse in future device fabrication. Integrating reclaimed LMs back into the production cycle without compromising performance or safety standards adds another layer of complexity. These technical and logistical barriers highlight the importance of designing LM‐based systems not only for functional performance and mechanical flexibility, but also with disassembly and material recovery in mind. Techniques such as distillation, electrolysis, and chemical precipitation can be used to purify extracted LMs, but their application to soft, multi‐material systems remains limited and energy‐intensive. Accordingly, near‐term priorities include oxide‐aware release chemistries, intermetallic‐resistant formulations, solvent‐lean workflows with closed‐loop solvent recovery, and recovery processes that preserve polymer value wherever possible. Addressing these challenges is vital to truly enable circular material flows, reduce dependence on virgin resources, and establish soft electronics as a pillar of sustainable and responsible manufacturing. However, only a few studies have demonstrated the practical reuse of reclaimed materials while evaluating device performance across multiple reuse cycles, indicating that this remains a largely underexplored area requiring significant innovation and validation. From a technical standpoint, the main bottlenecks are fast/selective oxide removal at scale, control of intermetallic formation, robust yet reworkable interfaces, and quality control of reclaimed LM; economically, solvent management/recapture, process energy, gallium price/availability, and collection logistics dominate—suggesting near‐term wins via closed‐loop solvents, sinter‐free processing, and tiered qualification so reclaimed LM is first reused in less‐demanding applications.

In summary, this review underscores that gallium‐based LMs and their composites combine metallic conductivity, stretchability, and reconfigurability with pathways for recycling, repair, and reuse. To translate these advantages into impact, the near‐term reality is hybridization rather than wholesale replacement of rigid PCBs; materials‐first design for recyclability (favoring thermoplastics or vitrimers where feasible), clear design labels/take‐back readiness for small devices, and oxide‐/intermetallic‐aware formulations are key. To achieve large‐scale impact in e‐waste reduction, future work must focus on scalable manufacturing methods such as high‐throughput printing and automated recovery systems, while also lowering the energy and chemical footprint of recycling processes. Major technical barriers include interfacial adhesion control, prevention of intermetallic formation, and maintaining device stability across reuse cycles, while economic challenges relate to the high cost of gallium, solvent management, and infrastructure requirements for collection and recycling. Nevertheless, the emergence of eco‐friendly solvents, bio‐derived elastomers, and electrochemical recovery strategies provides a strong foundation for sustainable scaling. In particular, vitrimer‐based LM composites—combining dynamic covalent reprocessability with robust functionality—represent a promising avenue for reconciling durability with circularity, further extending the 4R framework into practical applications. By embedding sustainability principles at both the material and system levels, LM composites can evolve from laboratory demonstrations to industrially viable, eco‐conscious technologies, contributing meaningfully to the circular electronics economy. By embedding these concrete steps and metrics into both materials development and recovery infrastructure, sustainable LM composites can move from laboratory demonstrations to industrially meaningful, circular solutions that measurably reduce e‐waste.

## Conflict of Interest

The authors declare no conflict of interest.
